# Novel Macrolactams from a Deep-Sea-Derived *Streptomyces* Species

**DOI:** 10.3390/md19010013

**Published:** 2020-12-29

**Authors:** Pei Wang, Dongyang Wang, Rongxin Zhang, Yi Wang, Fandong Kong, Peng Fu, Weiming Zhu

**Affiliations:** 1 Key Laboratory of Marine Drugs, Ministry of Education of China, School of Medicine and Pharmacy, Ocean University of China, Qingdao 266003, China; wangpei@itbb.org.cn (P.W.); wangdongyang@stu.ouc.edu.cn (D.W.); zrx1924@stu.ouc.edu.cn (R.Z.); wangyi0213@ouc.edu.cn (Y.W.); kongfandong@itbb.org.cn (F.K.); 2 Hainan Key Laboratory of Research and Development of Natural Product from Li Folk Medicine, Institute of Tropical Bioscience and Biotechnology, Chinese Academy of Tropical Agricultural Sciences, Haikou 571101, China; 3 Laboratory for Marine Drugs and Bioproducts, Pilot National Laboratory for Marine Science and Technology (Qingdao), Qingdao 266003, China

**Keywords:** macrolactam, Deep-Sea-Derived *Streptomyces*, abiotic formation, natural product, antifungal activity

## Abstract

Four polyene macrolactams including the previously reported niizalactam C (**4**), and three new ones, streptolactams A–C (**1**–**3**) with a 26-membered monocyclic, [4,6,20]-fused tricyclic and 11,23-oxygen bridged [14,16]-bicyclic skeletons, respectively, were isolated from the fermentation broth of the deep-sea sediment-derived *Streptomyces* sp. OUCMDZ-3159. Their structures were determined based on spectroscopic analysis, X-ray diffraction analysis, and chemical methods. The abiotic formation of compounds **2** and **4** from compound **1** were confirmed by a series of chemical reactions under heat and light conditions. Compounds **1** and **3** showed a selective antifungal activity against *Candida albicans* ATCC 10231.

## 1. Introduction

Macrolactams isolated from actinobacteria have become a large family of natural products, which showed lots of different biological activities and received more and more attention [1,2,3,4]. Some molecules with novel macrolactam frameworks and significant activities have been isolated from various actinobacterial strains, such as dracolactams [5], bombyxamycins [6], macrotermycins [7], verticilactam [8], sceliphrolactam [9], tripartilactam [10], and niizalactam C (**4**) [11]. The structure of tripartilactam has been revised to be the same as niizalactam C (**4**) [12]. It possesses a fused [18,6,6]-tricyclic system, which was proposed to be formed from sceliphrolactam via a spontaneous intramolecular [4 + 2] cycloaddition [12].

**Figure 1:**
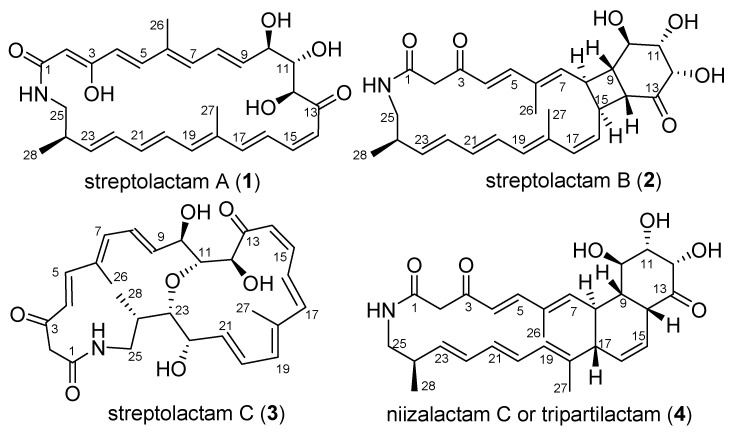
Chemical structures of compounds **1**–**4**.

We have been working on the active metabolites of marine-derived actinobacteria, especially on macrolactams [13,14,15,16]. Some interesting natural products with cytotoxic activity, represented by cyclamenols A−F [15,16], have been identified. To further discover new macrolactams for biological study from marine-derived actinobacteria, the deep-sea sediment-derived *Streptomyces* sp. OUCMDZ-3159 was selected. This strain was found to produce streptolactam A (**1**) as the main secondary metabolite. In addition, some trace analogues could be detected by LC-MS. In order to identify these structures, it was fermented on a 30 L scale. As a result, three novel macrolactams, streptolactams A–C (**1**–**3**), together with the reported niizalactam C (**4**) [11] (Figure 1), were isolated and identified. Structurally, streptolactam A (**1**) is a 26-membered cyclic polyene macrolactam [9], while streptolactam B (**2**) possesses a novel [20,4,6]-fused tricyclic skeleton, and streptolactam C (**3**) has an 11,23-oxgen bridged [14,16] bicyclic system. Herein, we reported the isolation, cytotoxic and antimicrobial activity, as well as the complete structural elucidation of compounds **1**–**4**.

## 2. Results and Discussion

### 2.1. Structural Elucidation

**Figure 2:**
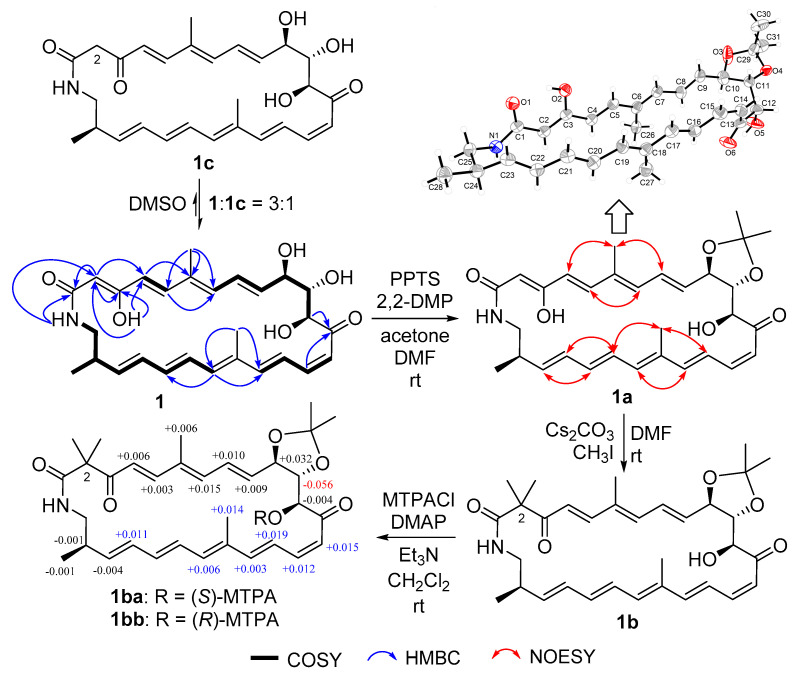
Structural elucidation of compound **1**.

Compound **1** was obtained as a yellow powder. Its molecular formula could be assigned as C_28_H_35_NO_6_ from the HRESIMS peak at *m/z* 482.2529 [M + H]^+^ (calcd 482.2537) (Figure S6 in supporting information). The similarity of ^1^H and ^13^C NMR data (Table 1) between compound **1** and sceliphrolactam [9] indicated that they share the same constitution, planar structure, which was confirmed by COSY of H-4/H-5, H-7/H-8/H-9/H-10/H-11/H-12, H-14/H-15/H-16/H-17, H-19/H-20/H-21/H-22/H-23/H-24/H_2_-25/NH, and H-24/H_3_-28 (Figure 2 and Figure S4), along with the key HMBC of NH to C-1 and C-2, H-2 to C-1, C-3 and C-4, H_3_-26 to C-5, C-6 and C-7, H-12 and H-15 to C-13, H_3_-27 to C-17 and C-19, and H-19 to C-17 (Figure 2 and Figure S5). However, absolute configuration of sceliphrolactam was not determined for its extreme sensitivity under light or heat conditions [9]. In the present study, we tried to solve this issue and first tried to elucidate the configuration of C-12 of compound **1** by preparing its acetonide (**1a**) followed by Mosher’s method [17] (Figure 2). During the process, the dimethylation derivative (**1b**) of **1a** was obtained (Figure 2). The acetonide **1a** was prepared by treating compound **1** with 2,2-dimethoxypropane (2,2-DMP) and pyridinium *p*-toluenesulfonate (PPTS) in acetone/DMF (3:1) (Figure 2). In the preparation of 3-*O*-methyl derivative of **1a**, we virtually obtained the 2,2-dimethyl derivative (**1b**). However, the Δ*δ* values between *S*-(**1ba**) and *R*-(**1bb**) Mosher esters of **1b** were some inconsistent (Figure 2 and Figures S41–S44), indicating that Mosher’s method cannot be used to determine the absolute configuration of this compound. Thus, we tried to elucidate the configuration of compound **1** by X-ray diffraction and luckily obtained the single-crystal of **1a**. A single-crystal X-ray diffraction pattern of **1a** was obtained using the anomalous scattering of Cu Kα radiation, allowing an explicit assignment of its absolute configurations as 10*R*, 11*S*, 12*S*, and 24*R* (Figure 2). Moreover, it should be noted that compound **1** could be isomerized to the corresponding 3-keto tautomer (**1c**) which reached a dynamic equilibrium with its 3-enol tautomer (**1**) in DMSO solution. The ^1^H and ^13^C NMR spectra showed some separate signals for **1** and **1c** with the approximate ratio of 3:1 in DMSO-*d*_6_ (Figures S1–S5). The diagnostic methylene signals (CH_2_-2) in **1c** were observed at *δ*_H/C_ (3.17, 3.44/50.8) (Table S2 and Figures S1–S5).

Careful comparison indicated that ^13^C data of compound **1** (Table 1) were obviously different with those reported for sceliphrolactam [9]. By removal of the calibration of the chemical shifts, we also noted that the most difference is for C-6, C-13 and C-14 which have –1.8, –1.9 and –1.3 ppm difference, respectively. In addition, the value of the specific rotation for compound **1** in the same methanol solution (−392.0 (*c* 0.05, MeOH)) is different from that of sceliphrolactam (−213 (*c* 0.09, MeOH)) [9]. Considering no identification of configuration for sceliphrolactam [9], it is reasonable to identify compound **1** as a stereoisomer of sceliphrolactam and a new compound.

**Figure 3:**
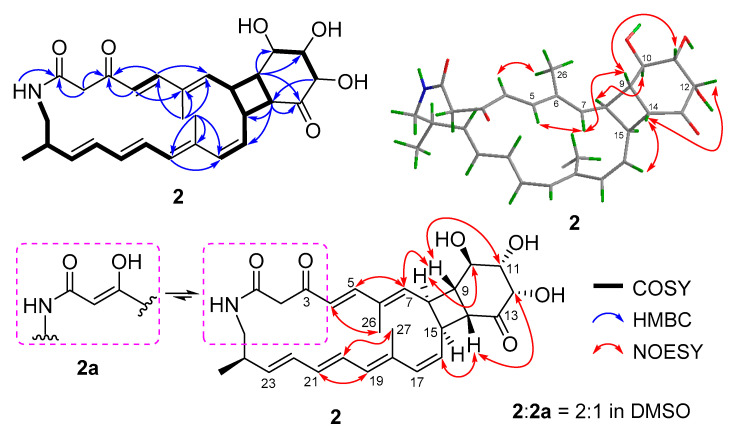
Key 2D NMR correlations of compound **2**.

Compound **2** was very unstable at room temperature (rt). So, we purified it at a relatively low temperature (18 °C) and measured its NMR spectra at 0 °C. The molecular formula of compound **2** was determined to be C_28_H_35_NO_6_ by HRESIMS (Figure S13). Comparison of its ^1^H and ^13^C NMR spectra (Table 1, Figures S7 and S8) with those of compound **4** revealed that they have the similar skeleton. The ^1^H and ^13^C NMR data (Table 1), assigned by HSQC (Figure S9), indicated the presence of twelve olefinic carbons, three carbonyl groups (*δ*_C_ 166.3 for amide carbonyl signal; *δ*_C_ 194.8 and 213.1 for keto carbonyl signals), eight sp^3^-methine groups including three oxygenated carbons, two methylene groups, and three methyl groups. Analysis of its COSY correlations of H-8/H-9/H-14/H-15 revealed the presence of a four-membered ring system that was fused with a six-membered ring, which was confirmed by the COSY correlations of H-10/H-11/H-12 and the key HMBC correlations of H-9 to C-10/C-11/C-13, H-12 to C-14, and H-14 to C-13/C-15/C-16 (Figure 3). The fused [20,4,6] tricyclic framework was determined by the key COSY correlations of H-4/H-5, H-7/H-8, H-15/H-16/H-17, H-24/H_3_-28, and extending from H-19 to H_2_-25, and the key HMBC correlations of NH to C-1, H-2 to C-1/C-3, H-4 to C-3/C-6, H-5 to C-3/C-7, H_3_-26 to C-5/C-6/C-7, and H_3_-27 to C-17/C-18/C-19 (Figure 3). This structure was deduced to form from the intramolecular [2 + 2] cycloaddition of compound **1**.

The geometries of double bonds at Δ^4^, Δ^20^, and Δ^22^ were assigned as *E*- by the ortho coupling constants (^3^*J*) of 15.0, 14.0, and 15.0 Hz, while the ^3^*J* value between H-16 and H-17 (11.4 Hz) indicated the *Z*-geometry of Δ^16^ double bond (Table 1). The *E*- geometries of Δ^6^ and Δ^18^ double bonds were determined by NOESY correlations of H-5/H-7 and H-20/H_3_-27 (Figure 3 and Figure S12). The relative configuration of [4,6]-bicyclic system was determined as (8S*,9S*, 10R*,11S*, 12S*,14R*, 15S*)-by the key NOESY correlations of H-7/H-9, H-14/H-16, H-9/H-11, H-12/H-14, and H-8/H-10 (Figure 3 and Figure S12). The fact that compound **2** could be formed from compound **1** (Figure 2) indicated the same (10*R*,11*S*,12*S*,24*R*)- configurations. Thus, the absolute configuration of compound **2** was determined as shown.

Compound **2**, a 3-keto tautomer, can also be reached a dynamic equilibrium with its 3-enol tautomer (**2a**) in pyridine solution (Figure 3). The ^1^H and ^13^C NMR spectra showed some separate signals for **2** and **2a** with the approximate ratio of 2:1 in pyridine-*d*_5_ (Figures S7–S11). The diagnostic NMR signals of sp^2^ methine at *δ*_H/C_ 5.53/95.3 and enol hydroxyl at *δ*_H_ 14.7 in **2a** could be observed (Table S2 and Figures S7–S11). It is interesting that the tautomerization between 3-enol and 3-keto in DMSO or pyridine solution was only observed for compounds **1** and **2**, but not for compounds **3** and **4**. This may be largely due to the size of ring. The 3-enol form could increase the ring tension, so that 3-keto form only exists in the small rings while 3-enol and 3-keto forms can coexist in the larger rings. 

**Figure 4:**
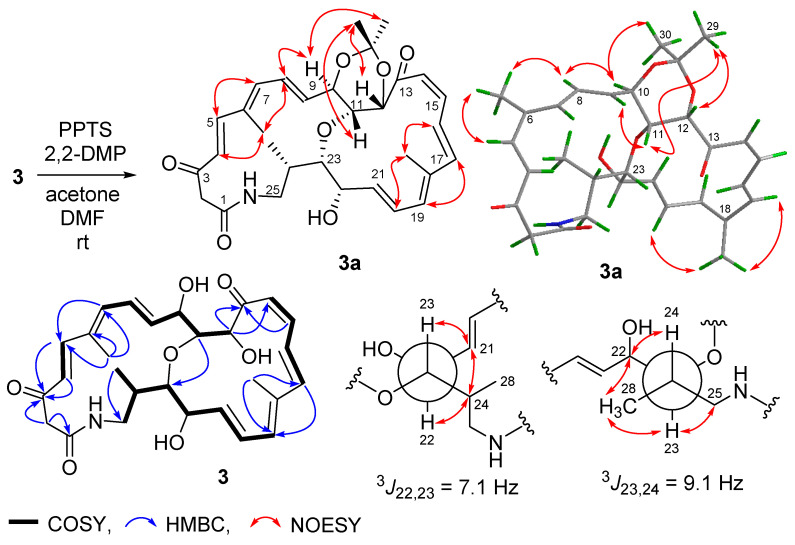
Preparation and determination of relative configuration of **3a**.

Streptolactam B (**3**) was obtained as a yellow powder. Its molecular formula was determined to be C_28_H_35_NO_7_ by HRESIMS (Figure S20). Comparison of its 1D NMR (Table 1, Figures S14 and S15) with those of compound **1** showed that the signals (*δ*_C/H_ 131.4/5.81 and *δ*_C/H_ 137.4/5.36) of a double bond in compound **1** were replaced by two oxygenated methine signals (*δ*_C/H_ 67.9/3.93 and 82.4/3.46) in compound **3**. The HMBC correlation of H-11 to C-23 (Figure 3, Figures S18 and S19) indicated that the carbons C-11 and C-23 were connected through an oxygen bridge. The COSY correlations extending from H-19 to H_2_-25 and the key HMBC correlations of H_2_-2 to C-1/C-3 (Figure 4 and Figure S18) further supported the structural difference between **1** and **3**.

**Figure 5:**
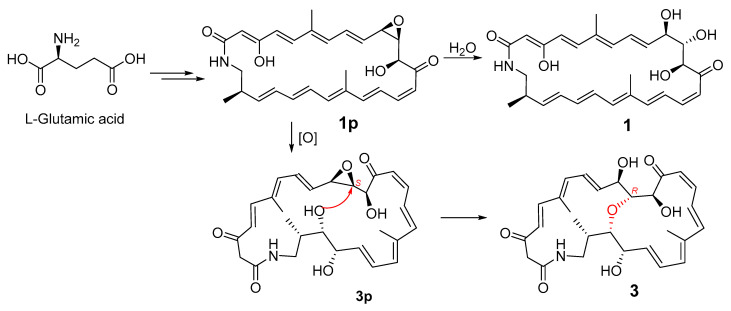
Postulated biosynthesis of compounds **1** and **3**.

The geometries of the Δ^4^, Δ^8^, Δ^16^, and Δ^20^ double bonds were determined as *E*- by the coupling constants of 15.4, 14.5, 14.7, and 14.4 Hz, respectively, while the Δ^14^ double bond was assigned as *Z*-geometry by the coupling constant of 11.5 Hz (Table 1). In order to verify the relative configuration of compound **3**, the acetonide derivative **3a** was prepared (Figure 4). The correlations of H-8/H_3_-26 and H-20/H_3_-27 could be observed in the NOESY spectrum of compound **3a** (Figure 4 and Figure S47), which indicated the *E*-geometries for the Δ^6^ and Δ^18^ double bonds. The (10R*, 11R*, 12S*)- relative configuration was determined by the NOESY correlations of H-9/H_3_-29, H-9/H-11, H-11/H_3_-29, H-10/H_3_-30, and H-12/H_3_-29 (Figure 4 and Figure S47). *J*-based configuration analysis (JBCA) method [18] was used to determine the relative configuration of C-22/C-23/C-24. The ^1^H NMR data of **3a** revealed the large coupling constants of H-22/H-23 (*J* = 7.1 Hz) and H-23/H-24 (*J* = 9.1 Hz) (Table 1). The NOESY correlations of H-21/H-23, H-21/H-24, and H-22/H-24 (Figure 4 and Figure S47) indicated *threo*-configuration between C-22 and C-23. The *threo*-configuration between C-23 and C-24 was concluded from the NOESY correlations of H-22/H-24, H-22/H_3_-28, H-23/H_3_-28, and H-23/H-25 (Figure 4 and Figure S47). In addition, compounds **1** and **3** might be biosynthetically formed from the same epoxide precursor, **1p**, which subjected to a dihydroxylation of Δ^22^ double bond followed by an etherification between HO-11 and 9,10-epoxide via an intramolecular nucleophilic ring opening reaction (Figure 5). Furthermore, compounds **1** and **3** showed the similar ECD Cotton effects from long wavelength (420 nm) to short wavelength (250 nm), that is negative first and then two positive effects, indicating they shared the same configurations at C-10 and C-12 which were nearest to the two conjugated enone chromophores, C-3–C-9 and C-13–C-21, and thus contributed most to the ECD Cotton effect. The absolute configuration of C-11 could be determined by comparing its relative configuration with C-10 and C-12 in compound **3a**, which is opposite to that of **1**. Thus, the absolute configurations of compound **3** were determined as shown. 

Compound **4** was further identical as niizalactam C or tripartilactam by spectroscopic and specific rotation data [10,11,12]. It is reasonable to strongly suggest that **1** and **4** shared a similar absolute configuration based on the fact that compound **4** could be formed from compound **1**.

**Figure 6:**
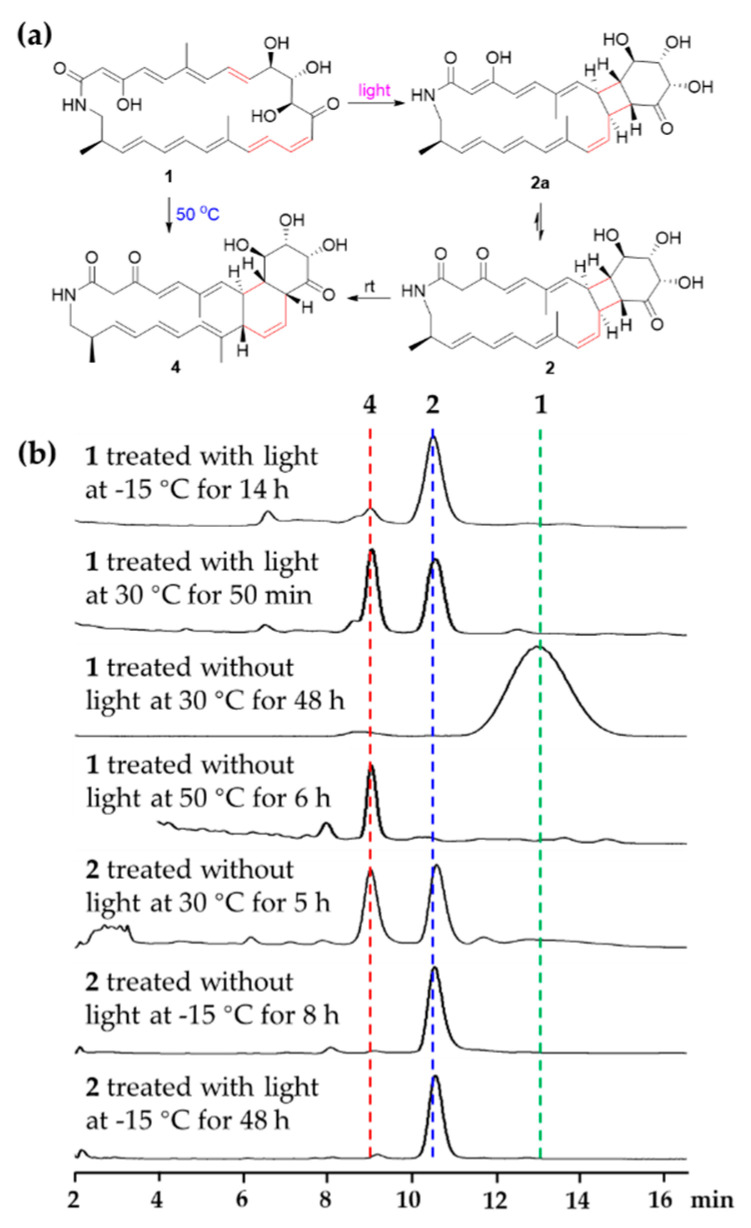
(**a**) Abiotic formation of compounds **2** and **4** from compound **1**. (**b**) HPLC profiles (320 nm) of different chemical transformations in MeOH.

During the isolation and structural elucidation of compound **1**, we noticed that compound **1** becomes unstable under light and heat and easy to form compounds **2** and **4**. In order to further understand the transformations among compounds **1**, **2** and **4**, a series of reactions were carried out under different conditions (Figure 6). Compound **1** could exist as a stable structure without light at the temperatures below 30 °C but exhaust and was transformed into compound **4** by the heat Diels-Alder reaction at 50 °C for 6 h, while compound **1** could be transformed into compound **2** via [2 + 2] cycloaddition by light (LED) at low temperature (−15 °C). In the latter reaction, only a little compound **4** was yielded. During the formation of compound **2**, only one product with a specific fusing mode, that is a [20,4,6]-fused tricyclic system, was generated, which might be caused by the geometries of double bonds and their relative position in the macrocycle. When compound **2** was placed at 30 °C, it could be transformed into compound **4**. At the low temperature (−15 °C) with or without light (LED), compound **2** was stable. These results demonstrated that compound **2** might be an important intermediate during the formation of **4** from **1** stored at rt without protection from light. So, light was the key factor causing compound **1** to change at rt. Avoiding light operation is an effective means to keep the polyene macrolactams stable. 

### 2.2. The Bioactivities of Compounds ***1***–***4*** from Streptomyces sp. OUCMDZ-3159

Compounds **1**, **3**, and **4** were evaluated for cytotoxicity against MCF-7, A549, K562, and HL-60 cell lines. No prominent cytotoxic activity against these cell lines was observed (IC_50_ > 50 μM). Their antimicrobial activity against pathogenic bacteria, *Escherichia coli* ATCC 11775, *Staphylococcus aureus* ATCC 6538, *Pseudomonas aeruginosa* ATCC10145, *Clostridium perfringens* CGMCC 1.0876 and *Bacillus subtilis* CGMCC 1.3376, as well as the pathogenic fungus, *Candida albicans* ATCC 10231 were also tested without light. It is interesting that only compounds **1** and **3** showed a selective antifungal activity against *C. albicans* with the MIC values of 10.4 and 16.1 μM, respectively. The result of compound **1** further corroborated the reported antifungal activity of the stereoisomer, sceliphrolactam [9]. The biological activity of compound **2** was not tested, because it is very fragile at rt. 

## 3. Materials and Methods

### 3.1. General Experimental Procedures 

Optical rotations were recorded with a JASCO P-1020 digital polarimeter (JASCO Corporation, Tokyo, Japan). UV spectra were recorded on a Beckman DU 640 spectrophotometer (Global Medical Instrumentation, Inc., Ramsey, MN, USA). IR spectra were obtained on a Nicolet Nexus 470 spectrophotometer in KBr discs (Thermo Fisher Scientific, Madison, WI, USA). NMR spectra were recorded on a Bruker Avance 600 spectrometer (Bruker BioSpin AG, Fällanden, Switzerland). ECD spectra were measured on JASCO J-815 spectrometer (JASCO Corporation, Tokyo, Japan). HRESIMS were measured on a Q-TOF Ultima Global GAA076 LC mass spectrometer (Waters Corporation, Milford, MA, USA). Semipreparative HPLC was performed using an ODS column (YMC-pack ODS-A, 10 × 250 mm, 5 μm, 4.0 mL/min). TLC and column chromatography (CC) were performed on plates pre-coated with silica gel GF254 (10–40 μm) and over silica gel (200–300 mesh, Qingdao Marine Chemical Factory, Qingdao, China), and Sephadex LH-20 (Amersham Biosciences, Uppsala, Sweden), respectively. Vacuum-liquid chromatography (VLC) was carried out over silica gel H (Qingdao Marine Chemical Factory).

### 3.2. Collection and Phylogenetic Analysis

The actinobacterial strain, *Streptomyces* sp. OUCMDZ-3159, was isolated from a deep-sea sediment collected at depth of 2782 m from the South Mid-Atlantic Ridge (15°9.972′ S, 13°21.348′ W) on October 31, 2012. The sample (2 g) was dried over 24 h in an incubator at 35 °C. The dried sample was diluted to 10–3 g/mL, 100 μL of which was dispersed across a solid-phase agar plate (10 g raffinose, 1 g L-histide, 0.5 g MgSO_4_.7H_2_O, 0.01 g FeSO_4_.7H_2_O, 0.1 g K_2_HPO_4_, 1.2 g bacto-agar, in 1 L seawater, pH 7.0) and incubated at 28 °C for 10 days. A single colony was transferred to Gause’s synthetic agar media. Analysis of the 16S rRNA gene sequence of OUCMDZ-3159 revealed 100% identity to *Streptomyces pratensis*. The sequence is deposited in GenBank under accession no. MT703834.

### 3.3. Cultivation and Extraction 

The spores of *Streptomyces* sp. OUCMDZ-3159 were directly transferred to 150 mL of a liquid medium (20 g glucose, 4 g yeast extract, 2 g peptone, 2 g CaCO_3_, 0.5 g MgSO_4_, 0.5 g K_2_HPO_4_, 0.5 g NH_4_SO_4_, in 1 L seawater) in Erlenmeyer flasks (500 mL) and shaken for 14 days (28 ± 0.5 °C, 180 rpm). The whole culture (30 L) was extracted with an equal volume of ethyl acetate (EtOAc) for three times and concentrated in vacuo to yield 20.5 g of EtOAc extract.

### 3.4. Purification 

The EtOAc extract (20.5 g) was separated into nine fractions (Fr.1–Fr.9) on a silica gel VLC column using step gradient elution with CH_2_Cl_2_−petroleum ether (0–50%) and then MeOH−CH_2_Cl_2_ (0–50%). Fraction 6 was separated into six fractions (Fr.6.1–Fr.6.6) by Sephadex LH-20 eluting with MeOH−CH_2_Cl_2_ (1:1) without light. Fr.6.3 was purified by semipreparative HPLC on an ODS column using the solvent system of 40% MeOH aqueous solution to yield compound **3** (16.0 mg, *t*_R_ 12.5 min). Fr.6.4 was purified by semipreparative HPLC on an ODS column using the solvent system of 65% MeOH aqueous solution to give compounds **4** (5.5 mg, *t*_R_ 9.1 min), **2** (1.2 mg, *t*_R_ 10.2 min), and **1 (**10.1 mg, *t*_R_ 12.3 min). Fraction 7 was fractionated into five subfractions (Fr.7.1−Fr.7.5) on a reversed-phase silica gel column, eluting with a step gradient of MeOH−H_2_O (10–100%) without light. Fr.7.3 and Fr.7.4 were purified by semipreparative HPLC on an ODS column using the solvent system of 65% MeOH and 40% MeOH aqueous solutions to yield compounds **1** (63 mg, *t*_R_ 12.3 min) and **3** (3.0 mg, *t*_R_ 12.5 min) without light, respectively. 

### 3.5. Preparation of Compounds ***1a*** and ***3a***

Compound **1** (25.0 mg) was dissolved in the mixture of DMF (6 mL) and acetone (2 mL), and then pyridinium *p*-toluenesulfonate (PPTS, 3.0 mg) and 2,2-dimethoxypropane (DMP, 200 μL) were added at 0 °C. The reaction mixture was stirred for 10 h at rt. Then 5 mL of H_2_O was added, and the solution was extracted three times with EtOAc (5 mL for each). The organic layer was combined and evaporated under reduced pressure to give a yellow gum that was subjected to HPLC purification eluting with 75% MeOH aqueous solution to give compound **1a** (15.3 mg, *t*_R_ 10.4 min, 56% yield). The same reaction of compound **3** (5.0 mg) was carried out and the product **3a** (2.0 mg, *t*_R_ 12.5 min, 37% yield) was purified by HPLC on an ODS column using 55% MeOH aqueous solution.

### 3.6. Preparation of Compound ***1b***

Compound **1a** (5.0 mg) was dissolved in 1 mL of dimethylformamide (DMF), then Cs_2_CO_3_ (2.0 mg) and CH_3_I (1 μL) were added at 0 °C. The reaction mixture was stirred for 2 h at 28 °C. Then 2 mL of H_2_O was added, and the solution was extracted three times with ethyl acetate (5 mL for each). The organic layer was combined and evaporated under reduced pressure to give a yellow gum that was purified by HPLC eluting with 80% MeOH aqueous solution to yield compound **1b** (2.0 mg, *t*_R_ 7.9 min, 38% yield).

### 3.7. Preparation of S-MTPA Ester (***1ba***) and R-MTPA Ester (***1bb***) of Compound ***1b***

Compound **1b** (1.0 mg) was dissolved in CH_2_Cl_2_ (1 mL), and then triethylamine (10 μL), dimethylaminopyridine (DMAP, 3.0 mg) and (*R*)-MTPACl (10 μL) were added. The reaction mixture was stirred for 6 h at rt. Then 1 mL of H_2_O was added, and the solution was extracted three times with CH_2_Cl_2_ (5 mL for each). The residue after removal of CH_2_Cl_2_ under reduced pressure was purified by semipreparative HPLC (90% MeOH) to yield (*S*)-MTPA ester **1ba** (1.0 mg, *t*_R_ 9.02 min). With the same method, (*R*)-MTPA ester **1bb** (1.0 mg, *t*_R_ 8.54 min) was obtained from the reaction of **1b** (1.0 mg) with (*S*)-MTPACl.

### 3.8. Characterization of the Compounds

Streptolactam A (**1**): yellow powder; ${\left[\alpha \right]}_{D}^{25}\;$ −392.0 (*c* 0.05, MeOH); ECD (1.00 *m*M, MeOH) λ_max_ (Δε) 216 (−8.8), 281 (+12.6), 330 (+4.0), 418 (−6.7) nm; UV (MeOH) λ_max_ (log ε) 279 (3.20), 332 (3.39), 421 (2.77) nm; IR (KBr) ν_max_ 3549, 3474, 3415, 3239, 2925, 2853, 1637, 1618, 1571, 1427, 1385, 1058, 619, 477 cm^−1^; ^1^H and ^13^C NMR, see Table S1; HRESIMS *m/z* 482.2529 [M + H]^+^ (calcd for C_28_H_36_NO_6_, 482.2537).

Streptolactam B (**2**): yellowed powder; ^1^H and ^13^C NMR at 0 °C, see Table 1; HRESIMS *m/z* 482.2548 [M + H]^+^ (calcd for C_28_H_36_NO_6_, 482.2537).

Streptolactam C (**3**): yellow solid; ${\left[\alpha \right]}_{D}^{15}\;$ −792.9 (*c* 0.05, MeOH); ECD (0.10 *m*M, MeOH) λ_max_ (Δε) 256 (+18.8), 319 (+4.1), 394 (−12.3) nm; UV (MeOH) λ_max_ (log ε) 401 (4.92), 319 (4.92), 296 (4.90) nm; IR (KBr) ν_max_ 3357, 2961, 2922, 1680, 1618, 1589, 1455, 1384, 1329, 1263, 1089, 1056, 976 cm^−1^; ^1^H and ^13^C NMR, see Table 1; HRESIMS *m/z* 498.2491 [M + H]^+^ (calcd for C_28_H_36_NO_7_, 498.2486).

Niizalactam C or Tripartilactam (**4**): yellow powder; ${\left[\alpha \right]}_{D}^{25}$ +30.0 (*c* 0.1, DMSO), −75.0 (*c* 0.1, MeOH); ECD (1.04 *m*M, MeOH) λ_max_ 201 (+5.24), 296 (−7.2) nm; ^1^H and ^13^C NMR, see Table S1; HRESIMS *m/z* 482.2544 [M + H]^+^ (calcd for C_28_H_36_NO_6_, 482.2537).

Compound **1a**: orange solid; ^1^H and ^13^C NMR, see Table S2; HRESIMS *m/z* 522.2864 [M + H]^+^ (calcd for C_31_H_40_NO_6_, 522.2850).

Compound **1b**: orange solid; ^1^H and ^13^C NMR, see Table S2; HRESIMS *m/z* 550.3167 [M + H]^+^ (calcd for C_33_H_44_NO_6_, 550.3163).

Compound **3a**: yellow solid; ^1^H NMR (600 MHz, DMSO-*d*_6_): *δ* 7.17 (1H, dd, *J* = 15.5, 11.5 Hz, H-16), 6.77 (1H, d, *J* = 15.5 Hz, H-5), 6.61 (1H, t, *J* = 11.5 Hz, H-15), 6.60 (1H, d, *J* = 15.5 Hz, H-17), 6.49 (1H, dd, *J* = 14.8, 11.5 Hz, H-8), 6.41 (1H, dd, *J* = 14.3, 11.5 Hz, H-20), 6.21 (1H, d, *J* = 11.5 Hz, H-14), 6.14 (1H, d, *J* = 15.5 Hz, H-4), 6.13 (1H, d, *J* = 14.8 Hz, H-7), 6.12 (1H, d, *J* = 11.5 Hz, H-19), 5.52 (1H, dd, *J* = 14.7, 9.0 Hz, H-9), 5.46 (1H, dd, *J* = 15.0, 10.3 Hz, H-21), 4.64 (1H, d, *J* = 1.8 Hz, H-12), 4.37 (1H, d, *J* = 9.0 Hz, H-10), 4.15 (1H, dd, *J* = 9.0, 1.8 Hz, H-11), 3.91 (1H, d, *J* = 18.1 Hz, H-2a), 3.88 (1H, dd, *J* = 10.3, 7.1 Hz, H-22), 3.86 (1H, dd, *J* = 11.2, 7.7 Hz, H-25a), 3.47 (1H, d, *J* = 18.1 Hz, H-2b), 3.44 (1H, dd, *J* = 9.1, 7.1 Hz, H-23), 2.77 (1H, t, *J* = 11.2 Hz, H-25b), 1.85 (1H, m, H-24), 1.81 (3H, s, CH_3_-26), 1.61 (3H, s, CH_3_-27), 1.01 (3H, d, *J* = 6.7 Hz, CH_3_-28), 1.33 (3H, s, CH_3_-29), 1.35 (3H, s, CH_3_-30); HRESIMS *m/z* 538.2809 [M + H]^+^ (calcd for C_31_H_40_NO_7_, 538.2799), 560.2628 [M + Na]^+^ (calcd for C_31_H_39_NO_7_Na, 560.2619).

*S*-MTPA ester (**1ba**) of **1b**: ^1^H NMR (600 MHz, CDCl_3_): *δ* 7.58 (1H, m, NH), 7.31 (1H, dd, *J* = 15.5, 12.0 Hz, H-16), 7.02 (1H, d, *J* = 14.8 Hz, H-4), 6.66 (1H, t, *J* = 11.5 Hz, H-15), 6.61 (1H, d, *J* = 15.1 Hz, H-17), 6.59 (1H, dd, *J* = 14.3, 11.4 Hz, H-8&21), 6.54 (1H, d, *J* = 15.1 Hz, H-5), 6.33 (1H, d, *J* = 12.5 Hz, H-19), 6.06 (1H, overlapped, H-7&14), 6.04 (2H, overlapped, H-20&22), 5.73 (1H, d, *J* = 2.3 Hz, H-12), 5.46 (1H, overlapped, H-9), 5.44 (1H, overlapped, H-23), 4.54 (1H, t, *J* = 8.1 Hz, H-10), 4.16 (1H, dd, *J* = 8.4, 2.3 Hz, H-11), 3.66 (1H, dd, *J* = 8.0, 4.6 Hz, H-25a), 2.60 (1H, ddd, *J* = 13.0, 10.7, 3.9 Hz, H-25b), 2.51 (1H, m, H-24), 1.87 (3H, s, H-26), 1.79 (3H, s, H_3_-27), 1.54 (3H, s), 1.42 (3H, s), 1.34 (3H, s), 1.05 (1H, d, *J* = 6.6 Hz, H_3_-28), 1.02 (3H, s); ESIMS *m/z* 766.5 [M + H]^+^.

*R*-MTPA ester (**1bb**) of **1b**: ^1^H NMR (600 MHz, CDCl_3_): *δ* 7.57 (1H, m, NH), 7.29 (1H, dd, *J* = 14.9, 11.0 Hz, H-16), 7.02 (1H, d, *J* = 15.1 Hz, H-4), 6.65 (1H, t, *J* = 11.2 Hz, H-15), 6.60 (1H, d, *J* = 15.0 Hz, H-17), 6.56 (1H, overlapped, H-8&21), 6.53 (1H, d, *J* = 14.9 Hz, H-5), 6.33 (1H, d, *J* = 11.3 Hz, H-19), 6.05 (1H, overlapped, H-7&14), 6.03 (2H, overlapped, H-20&22), 5.74 (1H, d, *J* = 2.2 Hz, H-12), 5.46 (1H, overlapped, H-9), 5.44 (1H, overlapped, H-23), 4.51 (1H, t, *J* = 8.1 Hz, H-10), 4.22 (1H, dd, *J* = 8.5, 2.2 Hz, H-11), 3.66 (1H, m, H-25a), 2.61 (1H, ddd, *J* = 18.2, 14.4, 4.0 Hz, H-25b), 2.51 (1H, m, H-24), 1.87 (3H, s, H_3_-26), 1.77 (3H, s, H_3_-27), 1.54 (3H, s), 1.43 (3H, s), 1.42 (3H, s), 1.35 (3H, s), 1.05 (1H, d, *J* = 6.6 Hz, H_3_-28); ESIMS *m/z* 766.4 [M + H]^+^.

### 3.9. X-Ray Crystallographic Analysis 

Compound **1a** was obtained as an orange crystal with molecular formula of C_31_H_39_NO_6_ from CH_2_Cl_2_/MeOH. Further, the crystal data were got on a Bruker Smart APEXDUO area detector diffractometer with graphite monochromated Cu-Kα radiation (λ = 1.54178 Å) (Table S4). The structure was solved by direct methods (SHELXS-97) and expanded using Fourier techniques (SHELXL-97). Crystallographic data (excluding structure factors) for structure **1a** in this paper have been deposited in the Cambridge Crystallographic Data Centre as supplementary publication number CCDC 996760. 

### 3.10. Chemical Interconversion of Compounds ***1***,***2*** and ***4***

Streptolactam A (**1**), isolated from an EtOAc extract from *Streptomyces* sp. OUCMDZ-3159 without light, was dissolved in MeOH at a concentration of 2.5 mM and stirred with or without light at rt (30 °C), −15 °C and 50 °C, respectively. Compound **2** was dissolved in MeOH at a concentration of 1 *m*M with or without light at rt (30 °C) and −15 °C, respectively. The reaction mixtures were analyzed every 30 min by use of HPLC on an ODS column eluting with 65% MeOH aqueous solution at a flow rate of 1 mL/min. The isolated compounds **1**, **2** and **4** were used as the standards (Figure 6).

Compound **2** was very fragile at rt. In order to identify its structure, we tried to obtain more materials through chemical transformation. Compound **1** was not stable under light, which could be converted into compounds **2** and **4**. So, we treated compound **1** (35.0 mg, 25mM in MeOH) with sunlight for 1.5 h, and then the mixture was separated by HPLC on an ODS column at 18 °C using 65% MeOH aqueous solution to yield compounds **4** (4.0 mg, *t*_R_ 9.1 min, 11.4% yield) and **2** (2.0 mg, *t*_R_ 10.2 min, 5.7% yield). The NMR spectra of compound **2** were measured at 0 °C in pyridine-*d*_5_.

### 3.11. Cytotoxicity Assay

Cytotoxicity was assayed against A549 and MCF-7 cell lines by the MTT [19], and K562 and HL-60 cell lines CCK-8 [20] methods. Adriamycin was used as the positive control with the IC_50_ values of 1.00, 0.63, 0.73 and 0.58 for the cell lines MCF-7, A549, K562, and HL-60 respectively.

### 3.12. Antimicrobial Assay 

The antimicrobial activities against pathogenic bacteria, *Escherichia coli* ATCC 11775, *Staphylococcus aureus* ATCC 6538, *Pseudomonas aeruginosa* ATCC10145, *Clostridium perfringens* CGMCC 1.0876 and *Bacillus subtilis* CGMCC 1.3376, as well as the pathogenic fungus, *Candida albicans* ATCC 10231 were evaluated by an agar dilution method [21]. The tested strains were cultivated in LB agar plates for bacteria and YPD agar plates for *C*. *albicans* at 37 °C. Compounds **1**, **3** and **4** and positive controls were dissolved in MeOH at different concentrations from 100 to 0.05 μg/mL by the continuous 2-fold dilution methods. A 10 μL quantity of test solution was absorbed by a paper disk (5 mm diameter) and placed on the assay plates. After 12 h incubation, zones of inhibition (mm in diameter) were recorded. The minimum inhibitory concentrations (MICs) were defined as the lowest concentration at which no microbial growth could be observed. Ciprofloxacin lactate (for bacteria) and ketoconazole (for fungus) were used as positive control for *E. coli*, *S. aureus*, *P. aeruginosa*, *C. perfringens*, *B. subtilis*, *C. albicans* with MIC values of 1.9, 1.9, 3.8, 1.9, 0.94, and 0.02 μM, respectively.

## 4. Conclusions

In summary, we identified four polyene macrolactams (**1**–**4**) from a deep-sea sediment-derived *Streptomyces* strain, OUCMDZ-3159. Compounds **1** and **2** with 20-membered or larger ring moieties existed a keto-enol tautomerism in the DMSO solution or pyridine solution. The abiotic formation of **2** and **4** from **1** was clarified through a package of heat and light induced intramolecular pericyclic reactions. This study indicated that light was a key factor that made the polyene macrolactams unstable at rt. The results provide ideas for the research on the non-enzymatic formation of polycyclic macrolactams. 

## Figures and Tables

**Table 1:** ^1^H (600 MHz) and ^13^C (150 MHz) NMR Data for Streptolactams A–C (**1**–**3**).

**No.**	**1 (in DMSO-*d*_6_)**		**2 (in Pyridine-*d*_5_) ^b^**		**3 (in DMSO-*d*_6_)**	
	*δ*_C_	*δ*_H_, mult. (*J* in Hz)	*δ*_C_	*δ*_H_, mult. (*J* in Hz)	*δ_C_*	*δ*_H_, mult. (*J* in Hz)
1	172.4, C		166.3, C		166.8, C	
2	93.7, CH	4.99, s	50.1, CH_2_	3.86, d (14.8); 3.65, d (14.8)	50.4, CH_2_	3.91, overlapped; 3.43, d (17.9)
3	165.8, C		194.8, C		193.3, C	
4	121.6 ^a^, CH	5.84, d (15.3)	123.4 ^a^, CH	6.27, d (15.0)	123.0, CH	6.11, d (15.4)
5	138.2, CH	6.54, d (15.3)	149.4, CH	7.28, d (15.0)	146.3, CH	6.74, d (15.4)
6	133.1, C		134.1, C		133.2, C	
7	135.9, CH	5.86, d (10.3)	146.2, CH	6.07, d (10.6)	141.0, CH	6.04, d (11.2)
8	128.4, CH	6.37, dd (13.8, 12.9)	42.6, CH	4.59, “t” like (10.2)	129.7, CH	6.32, dd (14.5, 11.6)
9	136.6, CH	5.43, dd (13.8, 7.8)	50.5, CH	3.52, “t” like (10.6)	139.0, CH	5.45, dd (14.6, 9.2)
10	70.0, CH	4.04, t, (7.8)	70.4, CH	4.40, m	70.0, CH	4.00, t (9.1)
11	74.3 ^a^, CH	3.79, d, (8.3)	81.8, CH	5.17, m	73.3, CH	3.82, overlapped
12	79.6, CH	4.26, brs	76.6, CH	5.62, m	79.7, CH	4.33, brs
13	199.1, C		213.1, C		198.6 ^a^, C	
14	119.9, CH	6.24, overlapped	51.8, CH	3.22, m	120.8, CH	6.13, d (11.5)
15	143.5, CH	6.68, t (11.2)	40.6, CH	4.31, m	143.8, CH	6.67, t (11.4)
16	125.6, CH	7.39, t (13.4)	129.6, CH	5.64, m	126.6, CH	7.39, dd (15.0, 11.8)
17	146.4, CH	6.64, d (15.0)	137.9, CH	6.21, d (11.4)	146.9, CH	6.61, d (15.3)
18	134.6 ^a^, C		136.3, C		136.2, C	
19	135.8, CH	6.24, overlapped	132.9, CH	6.03, d (11.0)	135.1, CH	6.14, d (11.6)
20	135.3 ^a^, CH	6.18, overlapped	128.0, CH	6.10, dd (14.0, 11.0)	129.2, CH	6.38, dd (14.4, 11.6)
21	127.4, CH	6.20, overlapped	133.7, CH	6.30, dd (14.0, 11.0)	137.6, CH	5.45, dd (14.6, 9.2)
22	131.4, CH	5.81, t (15.2, 9.2)	133.2, CH	5.96, dd (15.0, 11.0)	67.9, CH	3.93, overlapped
23	137.4, CH	5.36, m	137.7, CH	5.42, overlapped	82.4, CH	3.46, dd (8.5, 7.5)
24	39.5 ^a^, CH	2.20, brs	38.7, CH	2.59, m	38.5, CH	1.83, m
25	43.7, CH_2_	3.00, m; 3.06, m	46.3, CH_2_	3.02, m; 3.61, m	50.6, CH_2_	2.78 d (11.2, 10.9); 3.83, overlapped
26	12.0, CH_3_	1.80, s	13.0, CH_3_	1.86, s	12.4, CH_3_	1.77, s
27	12.0, CH_3_	1.60, s	16.0, CH_3_	1.58, s	12.5, CH_3_	1.61, s
28	16.2, CH_3_	1.00, d (6.5)	18.1, CH_3_	0.84, d (5.3)	14.8, CH_3_	0.99, d (6.4)
-NH		7.73, dd (5.4, 6.1)		8.34, brs		
3-OH		13.69, s				

^a^ Assigned from HMBC and HSQC spectra. ^b^ Measured at 0 °C.

## Supplementary Materials

The following are available online at https://www.mdpi.com/1660-3397/19/1/13/s1. Tables S1–S3: NMR data for compounds **4**, **1a**, **1b**, **1c** and **2a** and X-ray crystallographic analysis. Figure S1–S48: NMR and HRESIMS spectra.
