# Supplementary material for: Novel Macrolactams from a Deep-Sea-Derived Streptomyces Species
Source: Mar Drugs. 2020 Dec 29;19(1):13. doi: 10.3390/md19010013 (PMC7824713; doi:10.3390/md19010013)

Supplementary Information

Novel Macrolactams from a Deepsea-derived *Streptomyces* Species

Pei Wang [1,2†], Dongyang Wang [1†], Rongxin Zhang [1], Yi Wang [1], Fandong Kong [1,2], Peng Fu [1,3,*], and Weiming Zhu [1,3,*]

[1] Key Laboratory of Marine Drugs, Ministry of Education of China, School of Medicine and Pharmacy, Ocean University of China, Qingdao 266003, China; wangpei@itbb.org.cn (P.W.); wangdongyang@stu.ouc.edu.cn (D.W.); zrx1924@stu.ouc.edu.cn (R.Z.); wangyi0213@ouc.edu.cn (Y.W.); kongfandong@itbb.org.cn (F.K.);

[2] Hainan Key Laboratory of Research and Development of Natural Product from Li Folk Medicine, Institute of Tropical Bioscience and Biotechnology, Chinese Academy of Tropical Agricultural Sciences, Haikou 571101, China;

[3] Laboratory for Marine Drugs and Bioproducts, Pilot National Laboratory for Marine Science and Technology (Qingdao), Qingdao 266003, China;

* Correspondence: weimingzhu@ouc.edu.cn (W.Z.); fupeng@ouc.edu.cn (P.F.); Tel.: +86-532- 82031268 (W.Z.).

† These authors contributed equally to this work.

Table of Contents

Table S1. ^{1}H (600 MHz) and ^{13}C (150 MHz) NMR data for compounds **1a**,**1b** and **4** in DMSO-d_6………S4
Table S2. ^{1}H (600 MHz) and ^{13}C (150 MHz) NMR data of compounds **1c** and **2a**…………………………..S5
Table S3. Crystal data and structure refinement for compound **1a**………………………….…………..S5
Cytotoxicity Assay Protocol……………………….……………………………………………………S6
Figure S1 ^{1}H-NMR spectrum (600 MHz) of streptolactam A (**1**) in DMSO-d_6…………………………….S7
Figure S2. ^{13}C-NMR spectrum (150 MHz) of streptolactam A (**1**) in DMSO-d_6…………………………….S8
Figure S3. HSQC spectrum (600 × 150 MHz) of streptolactam A (**1**) in DMSO-d_6…………………….…S9
Figure S4. ^{1}H-^{1}H COSY spectrum (600 MHz) of streptolactam A (**1**) in DMSO-d_6………………….……S10
Figure S5. HMBC spectrum (600 × 150 MHz) of streptolactam A (**1**) in DMSO-d_6…………………….….S11
Figure S6. HRESIMS spectrum of streptolactam A (**1**)……………………………………………………S12
Figure S7. ^{1}H-NMR spectrum (600 MHz) of streptolactam B (**2**) in pyridine-d_5 at 0 °C………………S13
Figure S8. DEPTQ-NMR spectrum (150 MHz) of streptolactam B (**2**) in pyridine-d_5 at 0 °C………..S14
Figure S9. HSQC spectrum (600 × 150 MHz) of streptolactam B (**2**) in pyridine-d_5 at 0 °C…………..S15
Figure S10. ^{1}H-^{1}H COSY spectrum (600 MHz) of streptolactam B (**2**) in pyridine-d_5 at 0 °C…………..S16
Figure S11. HMBC spectrum (600 × 150 MHz) of streptolactam B (**2**) in pyridine-d_5 at 0 °C…………..S17
Figure S12. NOESY spectrum (600 MHz) of streptolactam B (**2**) in pyridine-d_5 at 0 °C…………………S18
Figure S13. HRESIMS spectrum of streptolactam B (**2**)……………………………………………………S19
Figure S14. ^{1}H-NMR spectrum (600 MHz) of streptolactam C (**3**) in DMSO-d_6…………………………S20
Figure S15. DEPTQ-NMR spectrum (150 MHz) of streptolactam C (**3**) in DMSO-d_6……………………S21
Figure S16. HSQC spectrum (600 × 150 MHz) of streptolactam C (**3**) in DMSO-d_6………………………S22
Figure S17. ^{1}H-^{1}H COSY spectrum (600 MHz) of streptolactam C (**3**) in DMSO-d_6………………………S23
Figure S18. HMBC spectrum (600 × 150 MHz) of streptolactam C (**3**) in DMSO-d_6………………………S24
Figure S19. Partial enlarged HMBC spectrum of streptolactam C (**3**)……………..………………..…S25
Figure S20. HRESIMS spectrum of streptolactam C (**3**)……………………………………………………S26
Figure S21. ^{1}H-NMR spectrum (600 MHz) of niizalactam C (**4**) in DMSO-d_6…………………………….S27
Figure S22. DEPTQ-NMR spectrum (150 MHz) of niizalactam C (**4**) in DMSO-d_6………………………S28
Figure S23. HSQC spectrum (600 × 150 MHz) of niizalactam C (**4**) in DMSO-d_6…………………………S29
Figure S24. ^{1}H-^{1}H COSY spectrum (600 MHz) of niizalactam C (**4**) in DMSO-d_6…………………………S30
Figure S25. HMBC spectrum (600 × 150 MHz) of niizalactam C (**4**) in DMSO-d_6…………………………S31
Figure S26. NOESY spectrum (600 MHz) of niizalactam C (**4**) in DMSO-d_6………………………………S32
Figure S27. HRESIMS spectrum of niizalactam C (**4**)………………………………………………………S33
Figure S28. ^{1}H-NMR spectrum (600 MHz) of compound **1a** in acetone-d_6………………………………S34
Figure S29. DEPTQ-NMR spectrum (150 MHz) of compound **1a** in acetone-d_6…………………………S35
Figure S30. HSQC spectrum (600 × 150 MHz) of compound **1a** in acetone-d_6…………………………….S36

Figure S31. ^{1}H-^{1}H COSY spectrum (600 MHz) of compound **1a** in acetone-d_6................................S37
Figure S32. HMBC spectrum (600 × 150 MHz) of compound **1a** in acetone-d_6................................S38
Figure S33. NOESY spectrum (600 MHz) of compound **1a** in acetone-d_6......................................S39
Figure S34. HRESIMS spectrum of compound **1a**..S40
Figure S35. ^{1}H-NMR spectrum (600 MHz) of compound **1b** in $CDCl_3$..S41
Figure S36. DEPTQ-NMR spectrum (150 MHz) of compound **1b** in $CDCl_3$..................................S42
Figure S37. HSQC spectrum (600 × 150 MHz) of compound **1b** in $CDCl_3$......................................S43
Figure S38. ^{1}H-^{1}H COSY spectrum (600 MHz) of compound **1b** in $CDCl_3$...................................S44
Figure S39. HMBC spectrum (600 × 150 MHz) of compound **1b** in $CDCl_3$...................................S45
Figure S40. HRESIMS spectrum of compound **1b**..S46
Figure S41. ^{1}H-NMR spectrum (600 MHz) of (*S*)-MTPA ester (**1ba**) of **1b** in $CDCl_3$........................S47
Figure S42. ^{1}H-^{1}H COSY spectrum (600 MHz) of (*S*)-MTPA ester (**1ba**) of **1b** in $CDCl_3$.....................S48
Figure S43. ^{1}H-NMR spectrum (600 MHz) of (*R*)-MTPA ester (**1bb**) of **1b** in $CDCl_3$........................S49
Figure S44. ^{1}H-^{1}H COSY spectrum (600 MHz) of (*R*)-MTPA ester (**1bb**) of **1b** in $CDCl_3$.................S50
Figure S45. ^{1}H-NMR spectrum (600 MHz) of compound **3a** in DMSO-d_6.....................................S51
Figure S46. ^{1}H-^{1}H COSY spectrum (600 MHz) of compound **3a** in DMSO-d_6................................S52
Figure S47. NOESY spectrum (600 MHz) of compound **3a** in DMSO-d_6......................................S53
Figure S48. HRESIMS spectrum of compound **3a**..S54

Table S1. ^{1}H (600 MHz) and ^{13}C (150 MHz) NMR data of compounds **1a**, **1b** and **4**

No.	**1a** (in acetone-d_6)		**1b** (in $CDCl_3$)		**4** (in DMSO-d_6)	
	δ_C	δ_H, mult. (J in Hz)	δ_C	δ_H, mult. (J in Hz)	δ_C	δ_H, mult. (J in Hz)
1	174.1[a], C		172.8[a], C		164.5, C	
2	94.8, CH	5.09, s	53.0[a], C		51.4, CH_2	3.47, d (11.5); 2.95, d (11.5)
3	166.6[a], C		202.1[a], C		191.3, C	
4	123.1, CH	5.92, d (15.4)	149.0, CH	7.00, d (15.0)	121.8, CH	6.03, d (15.4)
5	138.7, CH	6.69, d (15.4)	120.3, CH	6.54, d (15.3)	147.1, CH	7.09, d (15.4)
6	135.6, C		134.9, C		135.4, C	
7	135.3, CH	5.98, d (11.3)	138.8, CH	5.86, d (10.3)	145.6, CH	5.68, overlapped
8	133.0, CH	6.51, dd (14.8, 11.3)	131.9, CH	6.37, dd (13.8, 12.9)	38.1, CH	2.58, ddd (12.4, 10.5, 1.3)
9	132.6, CH	5.48, dd (14.8, 9.3)	132.9, CH	5.43, dd (13.8, 7.8)	48.6, CH	2.38, ddd (12.3, 9.3, 1.7)
10	76.3, CH	4.41, t (9.0)	75.1, CH	4.72, dd (3.7, 1.7)	70.3, CH	3.82, brs
11	81.0, CH	4.15, dd (8.7, 2.2)	81.1, CH	3.79, d (8.3)	78.7, CH	4.02, brs
12	75.7, CH	4.65, d (2.0)	75.0, CH	4.26, s	74.3, CH	4.52, brs
13	197.8, C		197.4, C		210.1, C	
14	119.6, CH	6.16, d (10.9)	118.9, CH	6.24, overlapped	44.4, CH	3.54, t (5.4)
15	146.3, CH	6.71, t (11.4)	146.1, CH	6.65, t (11.3)	130.2, CH	5.75, overlapped
16	126.1, CH	7.38, dd (15.1, 11.8)	124.4, CH	7.21, dd (14.9, 11.7)	132.5, CH	5.69, overlapped
17	149.2, CH	6.64, d (15.2)	149.6, CH	6.64, d (15.0)	52.2, CH	3.13, d (18.0)
18	135.9, C		134.8, C		134.3, C	
19	137.8, CH	6.22, overlapped	137.4, CH	6.24, overlapped	130.1, CH	5.75, overlapped
20	128.6, CH	6.26, m	128.2, CH	6.18, overlapped	125.8, CH	6.38, dd (14.4, 11.6)
21	136.6, CH	6.22, overlapped	135.8, CH	6.20, overlapped	130.4, CH	5.45, dd (14.6, 9.2)
22	133.4, CH	5.96, dd (15.4, 10.2)	133.8, CH	5.81, t (15.2, 9.2)	125.1, CH	3.93, overlapped
23	138.7, CH	5.29, dd (15.1, 9.5)	138.5, CH	5.36, m	137.9, CH	5.24, dd (15.0, 9.7)
24	42.0, CH	2.15, m	37.7, CH	2.53, dd, (3.1, 4.7)	38.3, CH	2.33, m
25	45.3, CH_2	3.42, t (11.6);	45.6, CH_2	3.71, t (8.2);	44.2, CH_2	3.26, dd (9.1, 3.7);
		2.79, dd (13.1, 4.1)		2.53, dd (10.7, 4.0)		2.88, td (13.0, 4.2)
26	12.5, CH_3	1.88, s	12.9, CH_3	1.87, s	13.2, CH_3	1.25, s
27	12.4, CH_3	1.68, s	12.7, CH_3	1.71, s	13.0, CH_3	1.49, s
28	17.2, CH_3	1.03, d (6.7)	17.7, CH_3	1.04, d (6.1)	19.0, CH_3	0.93, d (6.7)
29	109.1, C		109.0, C			
30	27.5, CH_3	1.364, s	26.7, CH_3	1.49, s		
31	26.9, CH_3	1.358, s	27.2, CH_3	1.44, s		
32			23.4, CH_3	1.55, s		
33			26.9, CH_3	1.40, s		
-NH		7.72, t (5.5)		7.62, d (5.2)		8.03, dd (8.1, 4.0)
3-OH		13.75, s				

[a] Assigned from HMBC and HSQC spectra.

Table S2. ^{1}H (600 MHz) and ^{13}C (150 MHz) NMR Data for **1c** and **2a**

No.	**1c** (in DMSO-d_6)		**2a** (in pyridine-d_5)[a]	
	δ_C	δ_H, mult. (J in Hz)	δ_C	δ_H, mult. (J in Hz)
1	165.0, C		174.2, C	
2	50.8, CH_2	3.44, d (14.0), 3.17, d (14.0)	95.3, CH	5.53 s
3	192.3[b], C		168.4[b], C	
4	124.0, CH	6.33, d (15.3)	121.4[b],CH	5.93, d (15.0)
5	145.2, CH	6.80, d (15.3)	140.6, CH	7.13, d (15.0)
6	132.5, C		134.7, C	
7	138.7, CH	6.10, d (11.3)	140.0, CH	5.78, d (10.8)
8	125.8, CH	6.55, overlapped	41.8, CH	4.50, m
9	138.8, CH	5.62, dd (15.0, 4.2)	50.6, CH	3.55, "t" like (10.1)
10	70.6, CH	4.15, brs,	70.6, CH	4.42, brs
11	75.8, CH	3.66, brd (8.0)	81.8, CH	5.15, overlapped
12	80.2, CH	4.37, brs	76.6, CH	5.59, overlapped
13	200.4 [b], C		213.0[b], C	
14	121.6, CH	6.18, overlapped	51.8, CH	3.55, m
15	142.4, CH	6.63, t (11.0)	41.2, CH	4.33, m
16	124.1, CH	7.30, dd (14.5, 12.0)	129.4[b],CH	5.51, overlapped
17	146.6, CH	6.64, overlapped	138.0, CH	6.16, d (11.4)
18	133.9, C		136.5 C	
19	136.0, CH	6.20, overlapped	132.9, CH	6.03, d, (11.0)
20	127.9, CH	6.39, overlapped	127.9, CH	6.27, m
21	135.1, CH	6.16, overlapped	133.6, CH	6.30, m
22	131.7, CH	6.00, dd (15.0,10.9)	133.6, CH	6.09,m
23	137.5, CH	5.38, m	138.2, CH	5.58, overlapped
24	37.8, CH	2.30, brs	43.1, CH	2.37, m
25	44.1, CH_2	2.89, m; 2.91, m	45.8, CH_2	2.86, m; 3.96, m
26	12.2, CH_3	1.73, s	13.1, CH_3	1.72, s
27	12.8, CH_3	1.89, s	15.3, CH_3	1.50, s
28	17.6, CH_3	0.97, d (6.7)	17.9, CH_3	0.89, d (5.3)
-NH		7.94, dd (6.6, 2.8)		8.54, brs
3-OH				14.7, s

[a] Measured at 0 °C; [b] Assigned from HMBC and HSQC spectra.

Table S3. Crystal data and structure refinement for compound **1a**

Identification code	121228e
Empirical formula	$C_{31}H_{39}NO_6$
Formula weight	521.63
Temperature	293(2) K
Wavelength	1.54178 Å
Crystal system, space group	Orthorhombic, P2 (1) 2 (1) 2 (1)

Unit cell dimensions	a = 7.6506(5) Å alpha = 90 deg.
	b = 10.0847(7) Å beta = 90 deg.
	c = 36.725(3) Å gamma a = 90 deg.
Volume	2833.5(3) $Å^3$
Z, Calculated density	4, 1.223 mg/m^3
Absorption coefficient	0.679 mm^{-1}
F(000)	1120
Crystal size	0.38 x 0.35 x 0.18 mm
Theta range for data collection	4.55 to 66.02 deg.
Limiting indices	-9<=h<=7, -11<=k<=H<=11, -43<=l<=33
Reflections collected / unique	8918 / 4621 [R(int) = 0.0255]
Completeness to theta = 66.02	99.80%
Absorption correction	Semi-empirical from equivalents
Max. and min. transmission	0.8875 and 0.7824
Refinement method	Full-matrix least-squares on F^2
Data / restraints / parameters	4621 / 0 / 349
Goodness-of-fit on F^2	1.107
Final R indices [I>2sigma(I)]	R1 = 0.0512, wR2 = 0.1318
R indices (all data)	R1 = 0.0604, wR2 = 0.1366
Absolute structure parameter	-0.1(4)
Extinction coefficient	0.00076(10)
Largest diff. peak and hole	0.217 and -0.307 e. $Å^{-3}$

Cytotoxicity Assay Protocol:

Cytotoxicity was assayed by the MTT [19] and CCK-8 [20] methods. In the MTT assay, A549 or MCF-7 cell line was grown in RPMI-1640 supplemented with 10% FBS under a humidified atmosphere of 5% CO_2 and 95% air at 37 °C, respectively. Cell suspension, 100 μL, at a density of 3 × 10^4 cell/mL was plated in 96-well microtiter plates, allowed to attach overnight, and then exposed to varying concentrations (10-5–10-12 M) of compounds **1**, **2** and **3** for 72 h. The MTT solution (20 μL, 5 mg/mL in IPMI-1640 medium) was then added to each well and incubated for 4 h. Old medium containing MTT was then gently replaced by DMSO and pipetted to dissolve any formazan crystals formed. Absorbance was then determined on a Spectra Max Plus plate reader at 570 nm. In the CCK-8 assay, K562 or HL-60 cell line was grown in RPMI-1640 supplemented with 10% FBS under a humidified atmosphere of 5% CO_2 and 95% air at 37 °C. Cell suspension, 100 μL, at a density of 5 × 10^4 cell/mL was plated in 96-well microtiter plates and then exposed to varying concentrations (10^{-5}–10^{-12} M) of compounds after cultivation for 24 h. Three days later, 10 μL of CCK-8 solution was added 4 h before detection. Then the absorbency (A450 value) was measured, and the growth rates of cells were computed. Adriamycin was used as the positive control with the IC_{50} values of 1.00, 0.63, 0.73 and 0.58 for the cell lines MCF-7, A549, K562, and HL-60 respectively.

Figure S1. ^{1}H-NMR spectrum (600 MHz) of streptolactam A (**1**) in DMSO-*d*$_6$

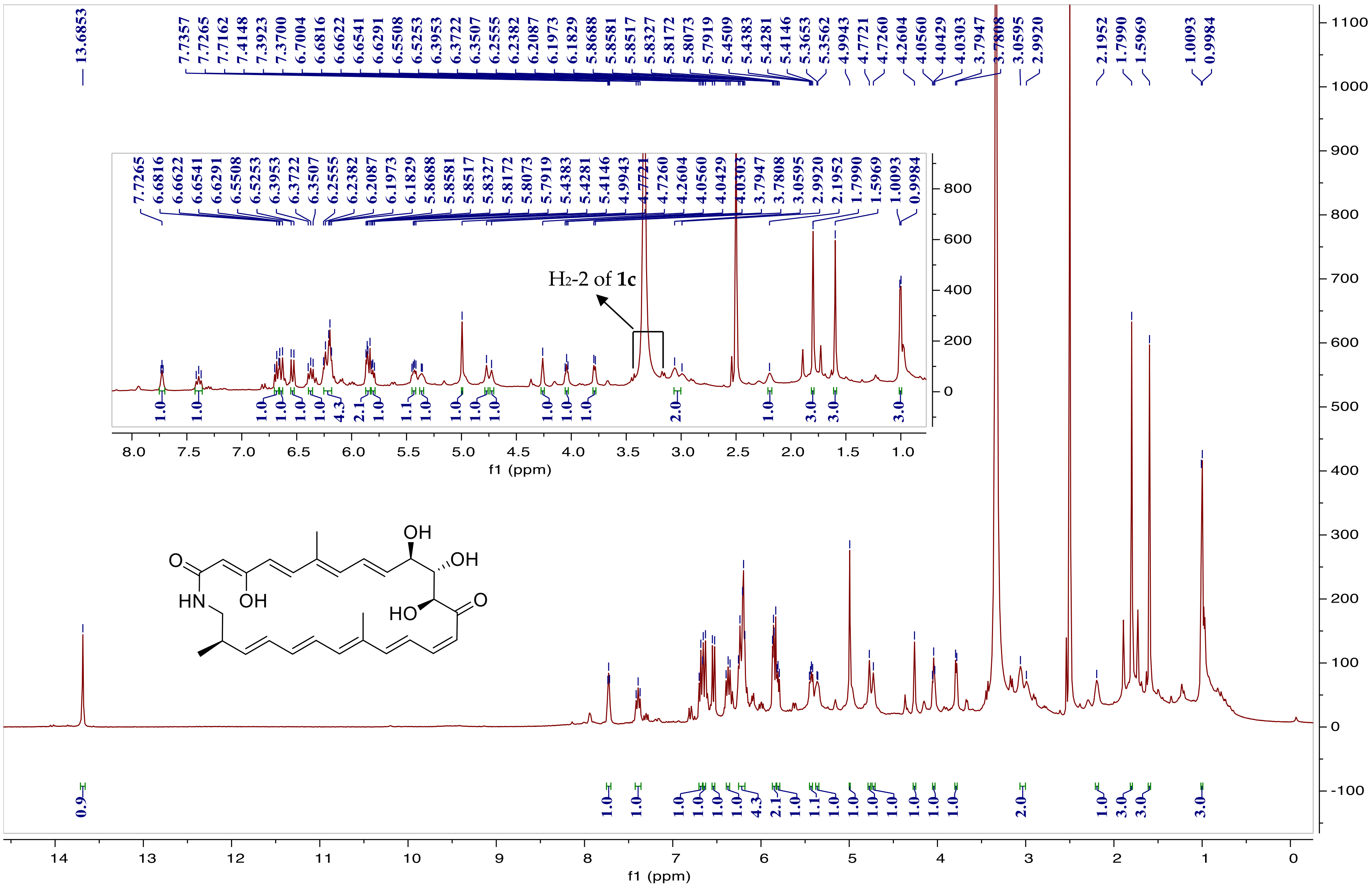

Figure S2. ^{13}C-NMR spectrum (150 MHz) of streptolactam A (**1**) in DMSO-d_6

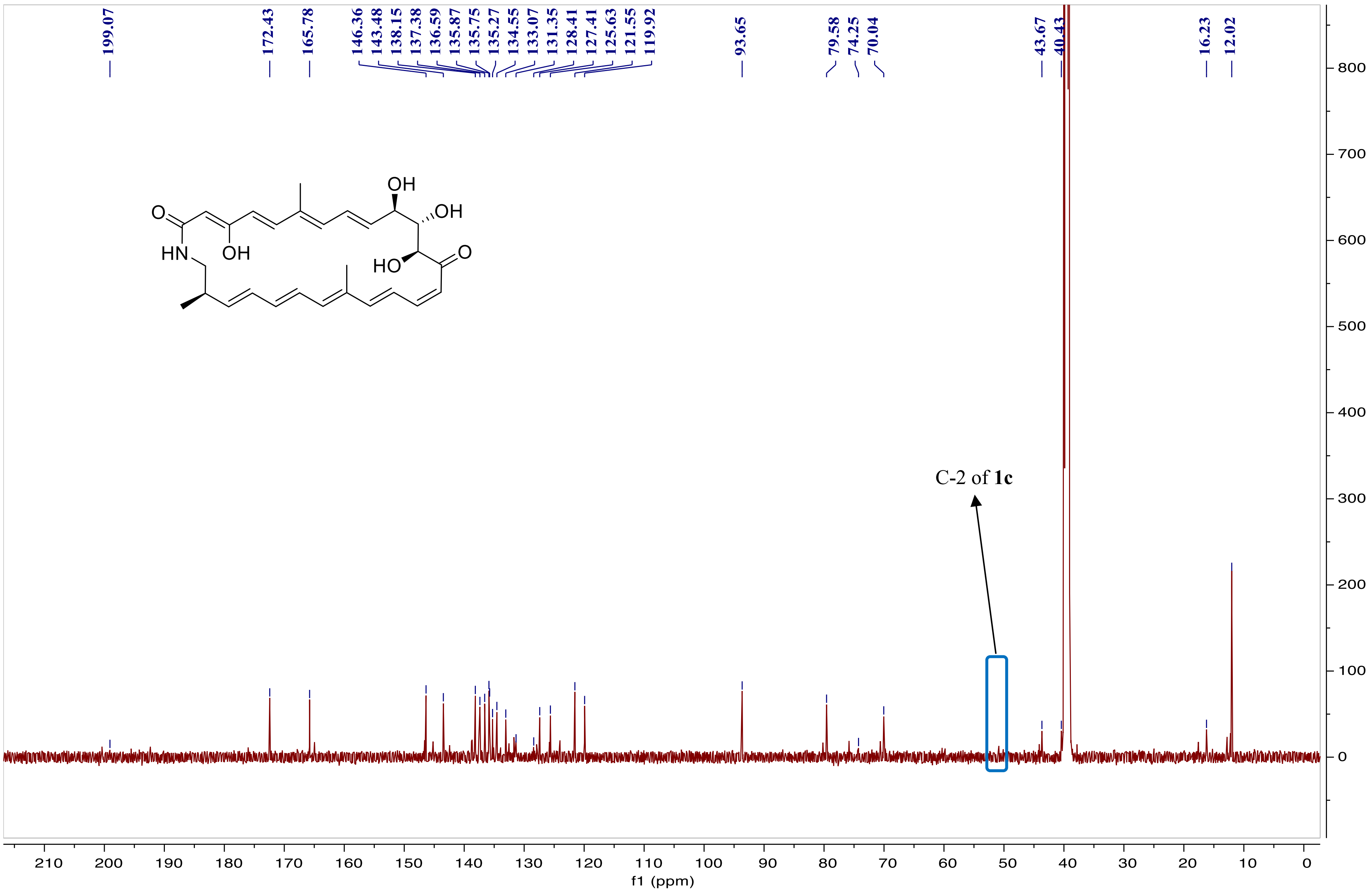

Figure S3. HSQC spectrum (600 × 150 MHz) of streptolactam A (**1**) in DMSO-d_6

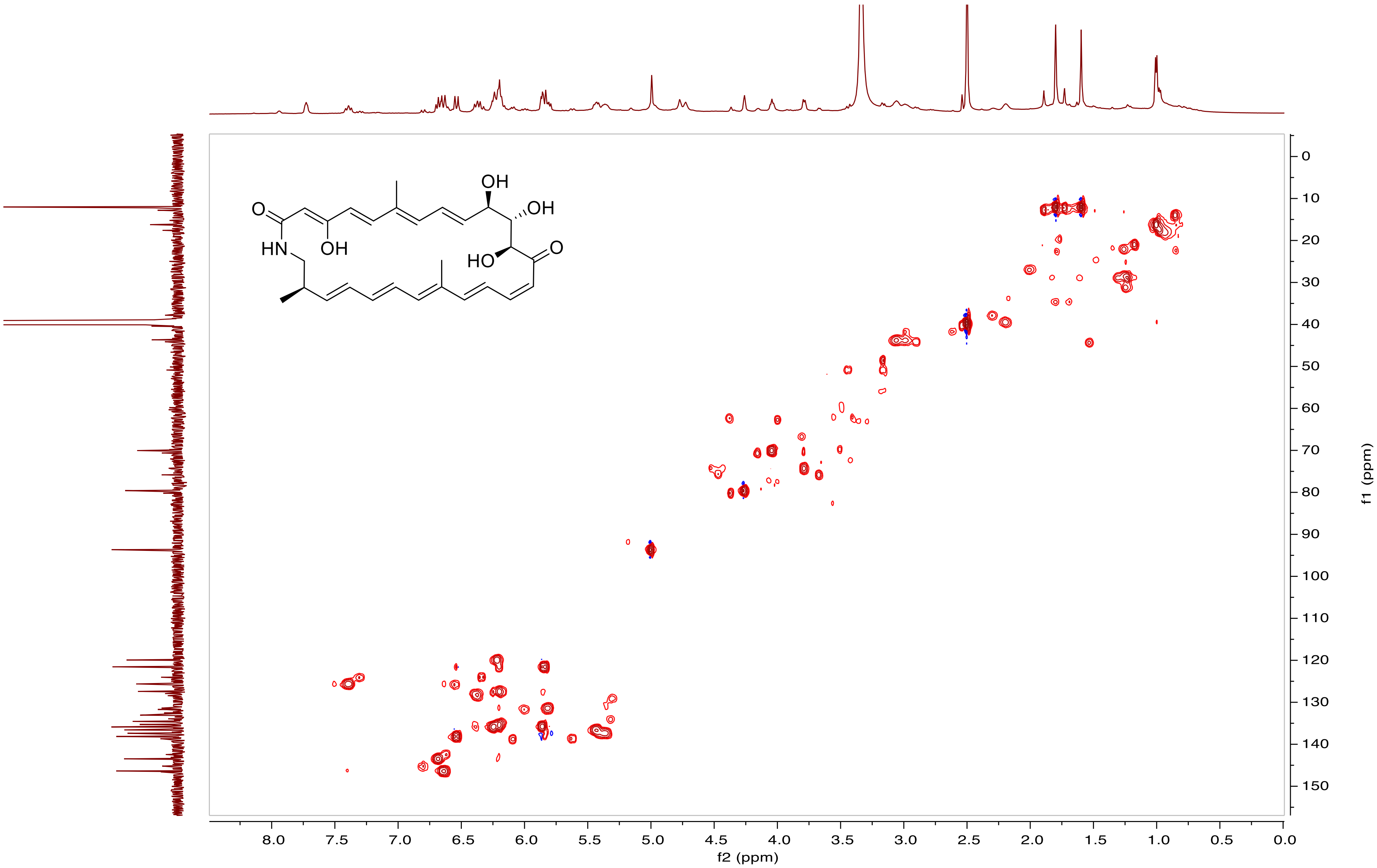

Figure S4. ^{1}H-^{1}H COSY spectrum (600 MHz) of streptolactam A (**1**) in DMSO-d_6

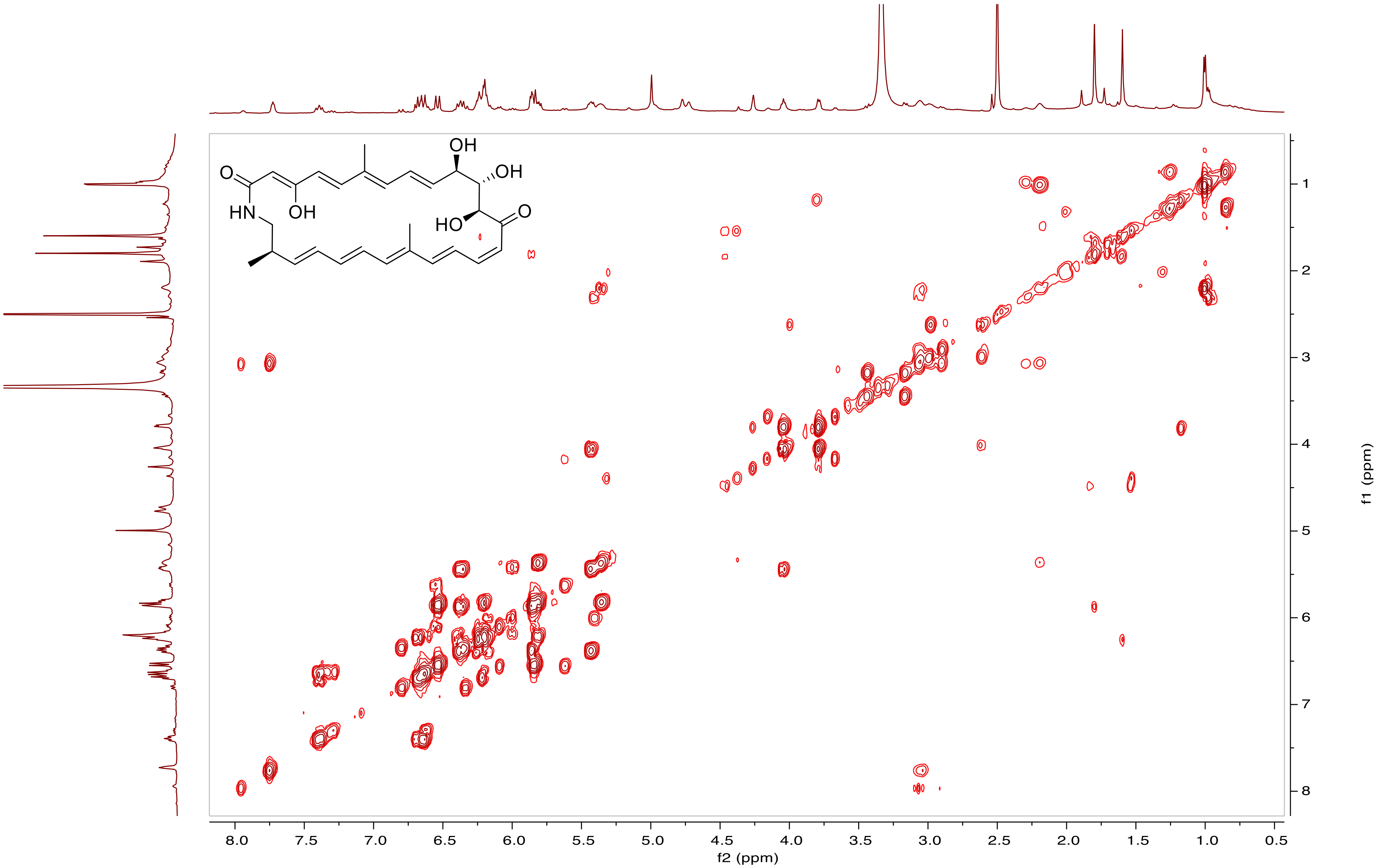

Figure S5. HMBC spectrum (600 × 150 MHz) of streptolactam A (**1**) in DMSO-d_6

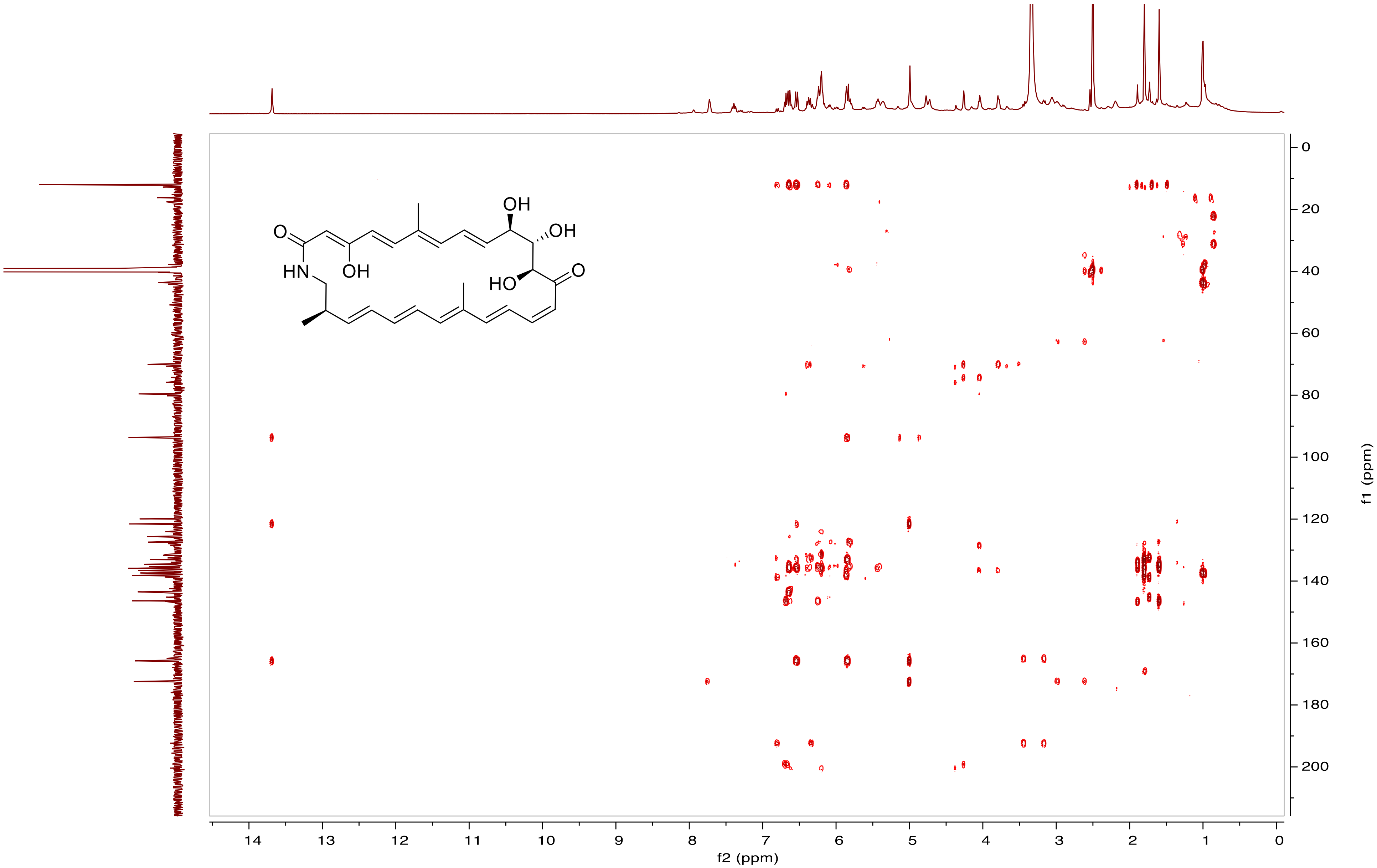

Figure S6. HRESIMS spectrum of streptolactam A (**1**)

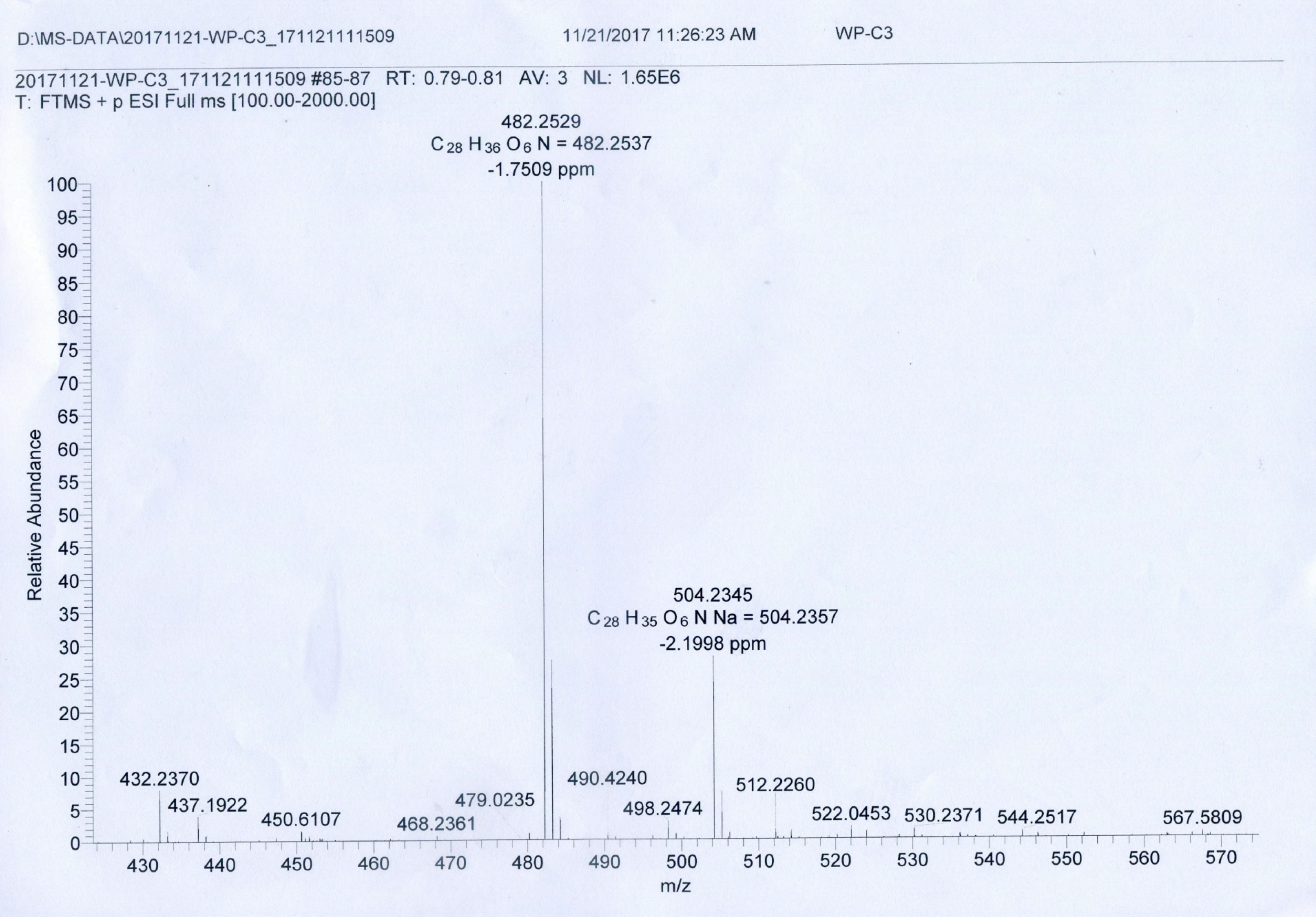

Figure S7. ^{1}H-NMR spectrum (600 MHz) of streptolactam B (**2**) in pyridine-d_5 at 0 °C

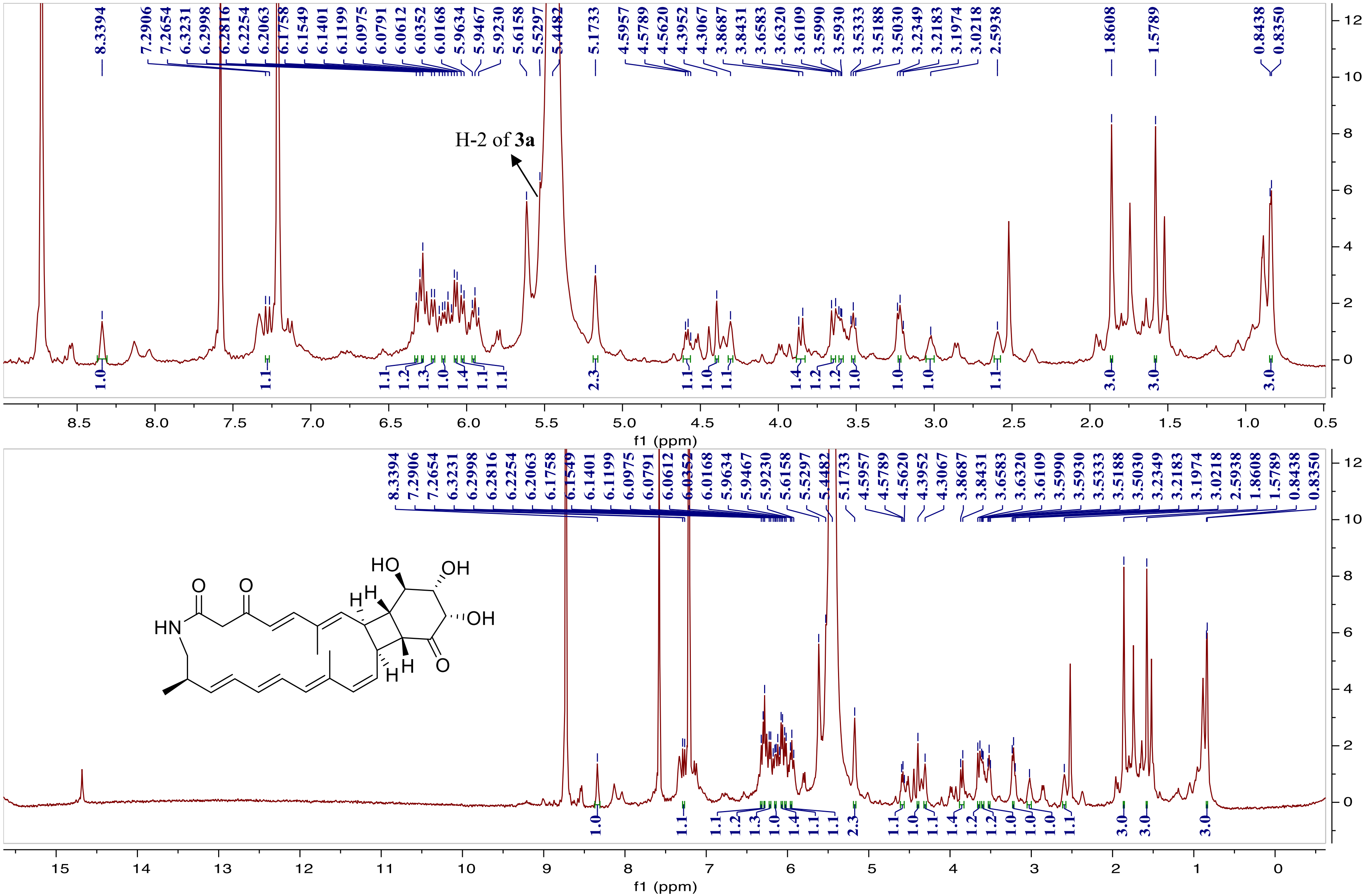

Figure S8. DEPTQ-NMR spectrum (150 MHz) of streptolactam B (**2**) in pyridine-d_5 at 0 °C

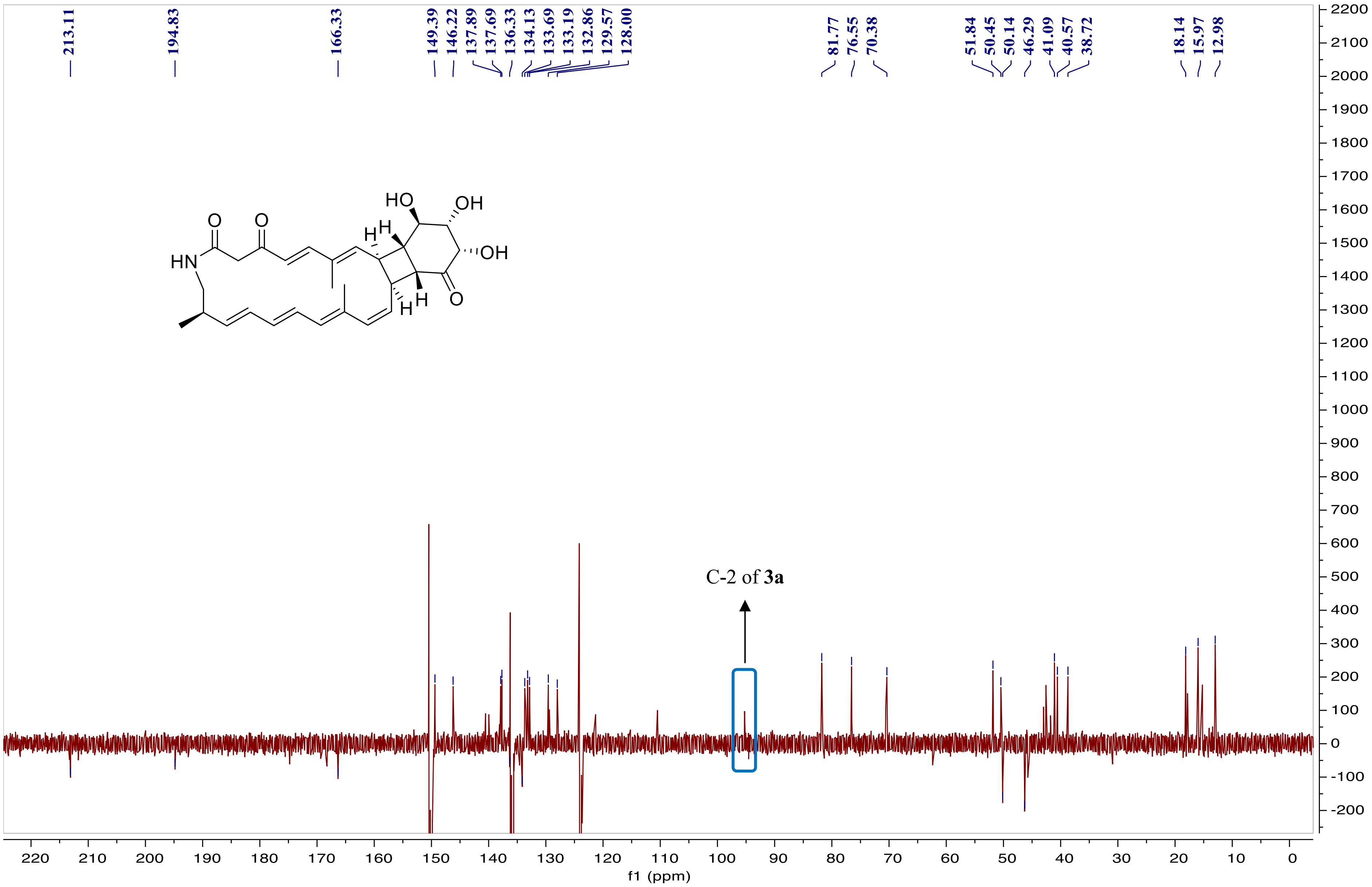

Figure S9. HSQC spectrum (600 × 150 MHz) of streptolactam B (**2**) in pyridine-d_5 at 0 °C

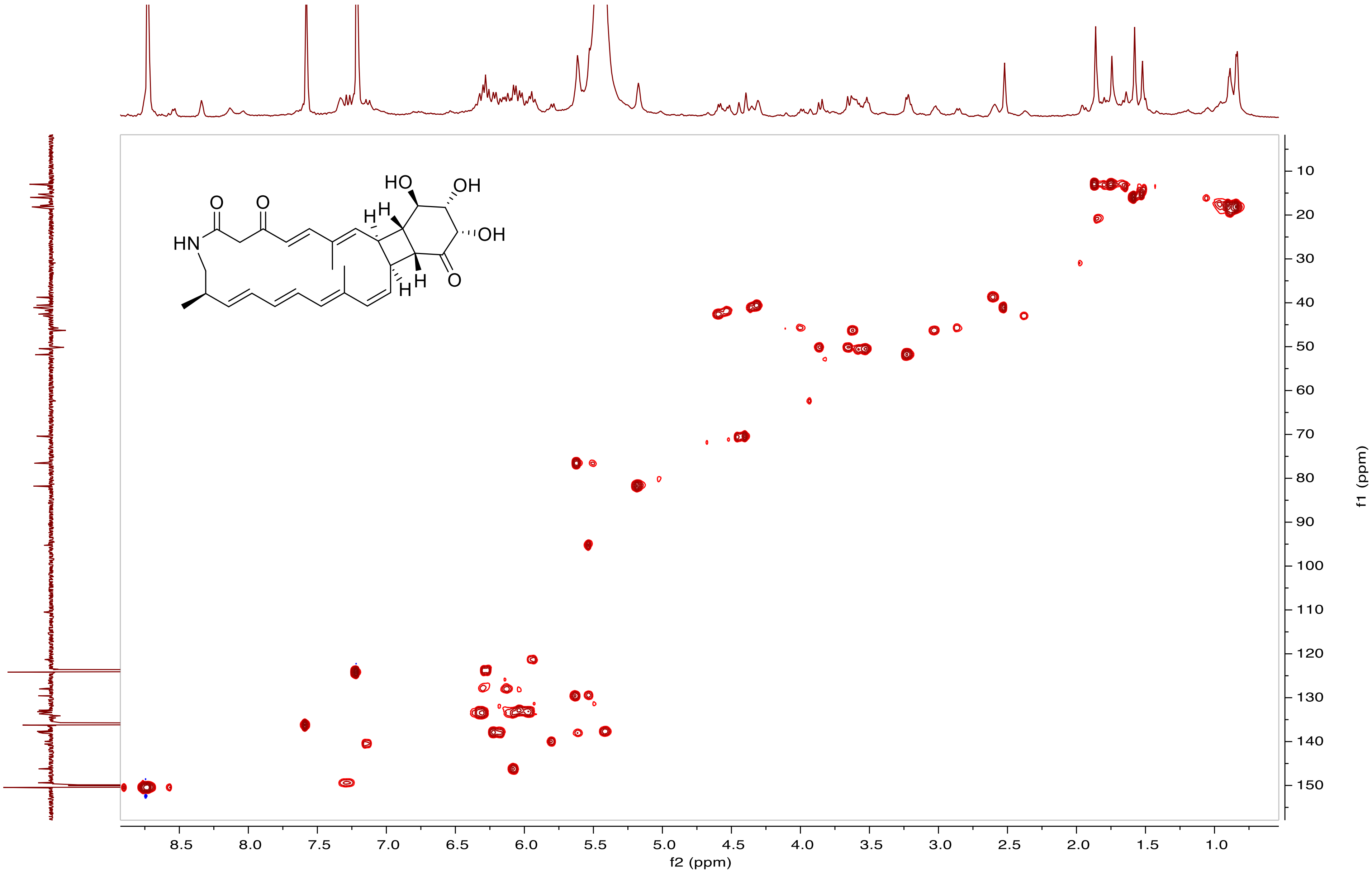

Figure S10. ¹H-¹H COSY spectrum (600 MHz) of streptolactam B (**2**) in pyridine-*d*5 at 0 °C

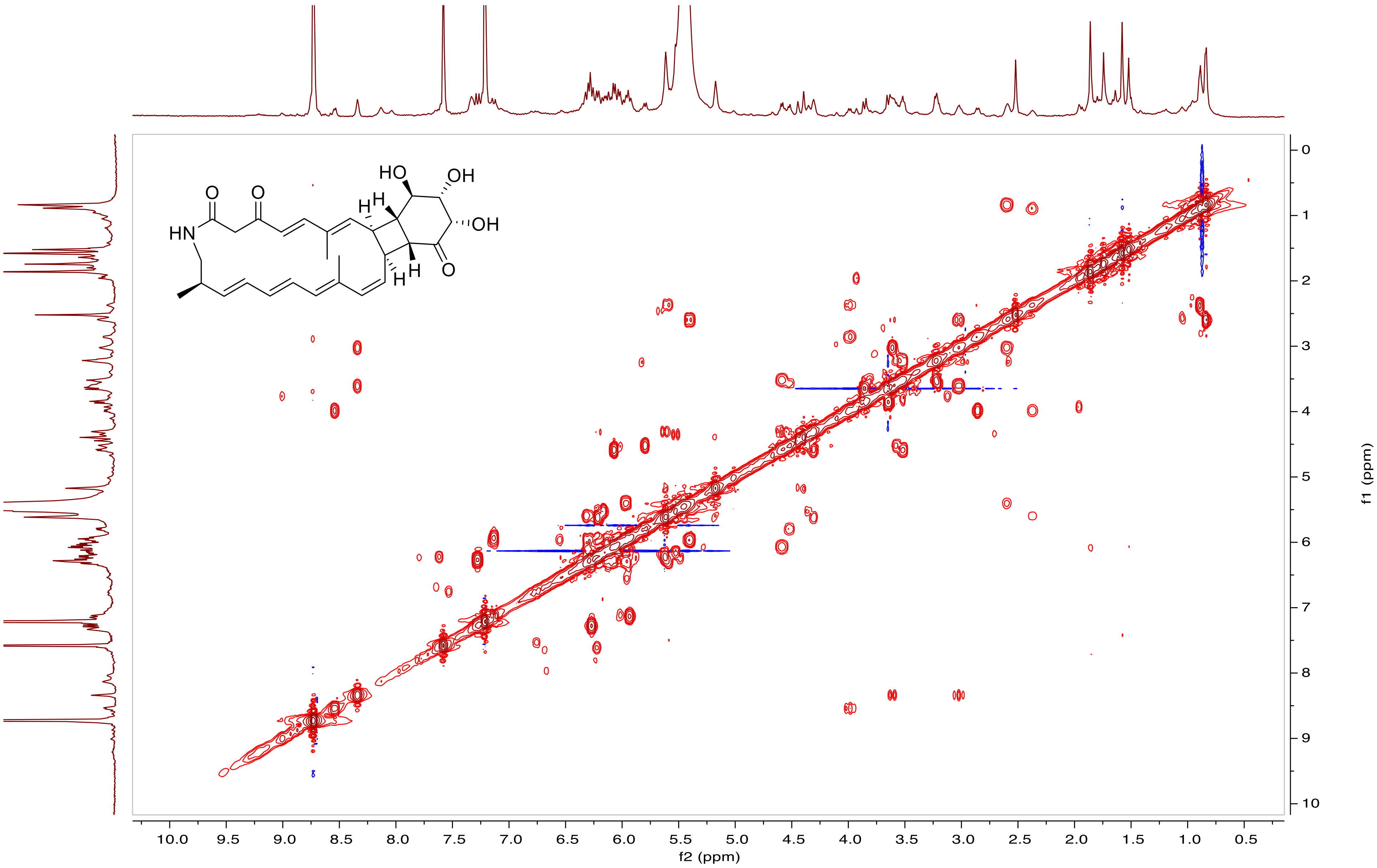

Figure S11. HMBC spectrum (600 × 150 MHz) of streptolactam B (**2**) in pyridine-*d*5 at 0 °C

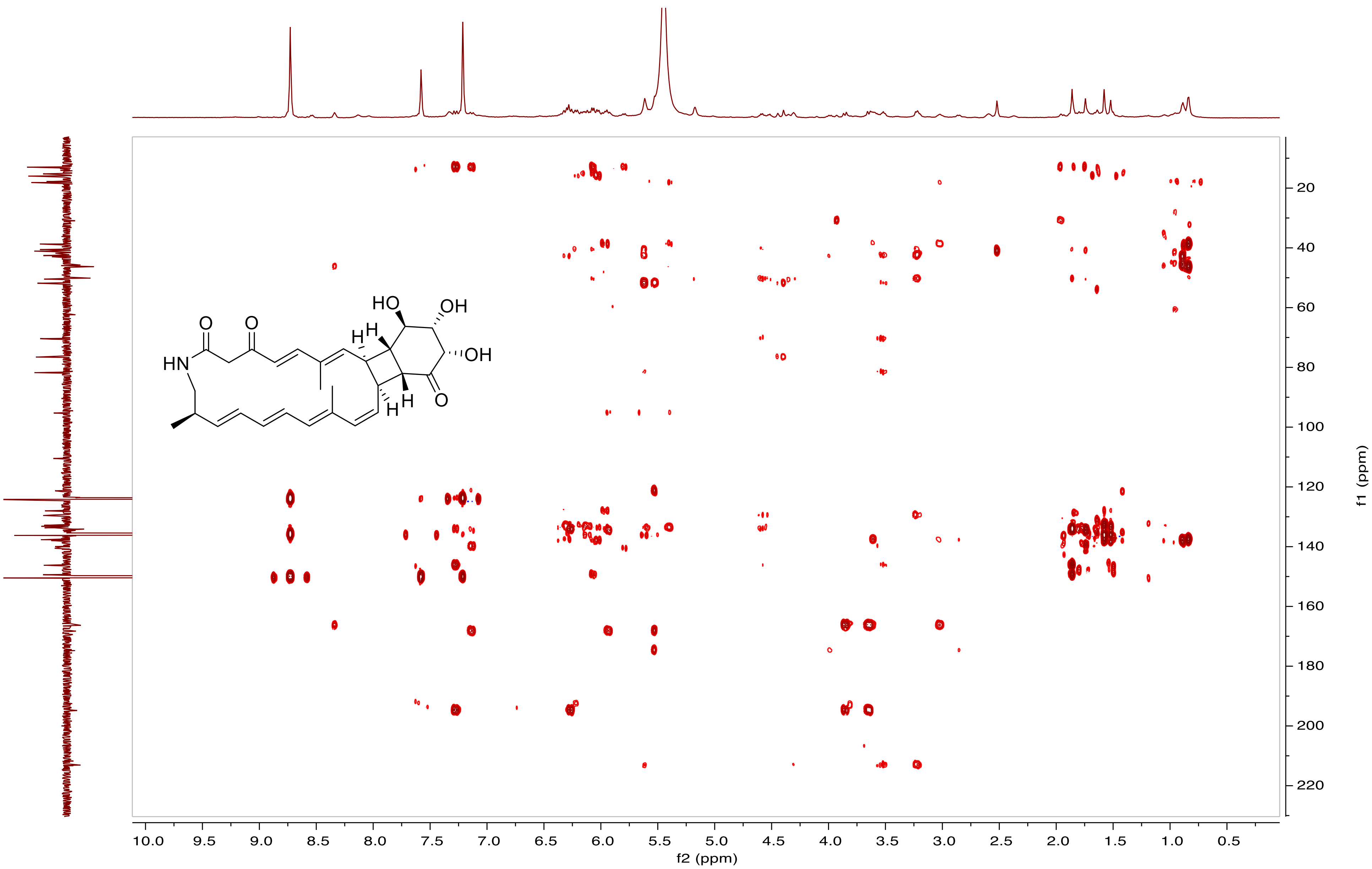

Figure S12. NOESY spectrum (600 MHz) of streptolactam B (**2**) in pyridine-d_5 at 0 °C

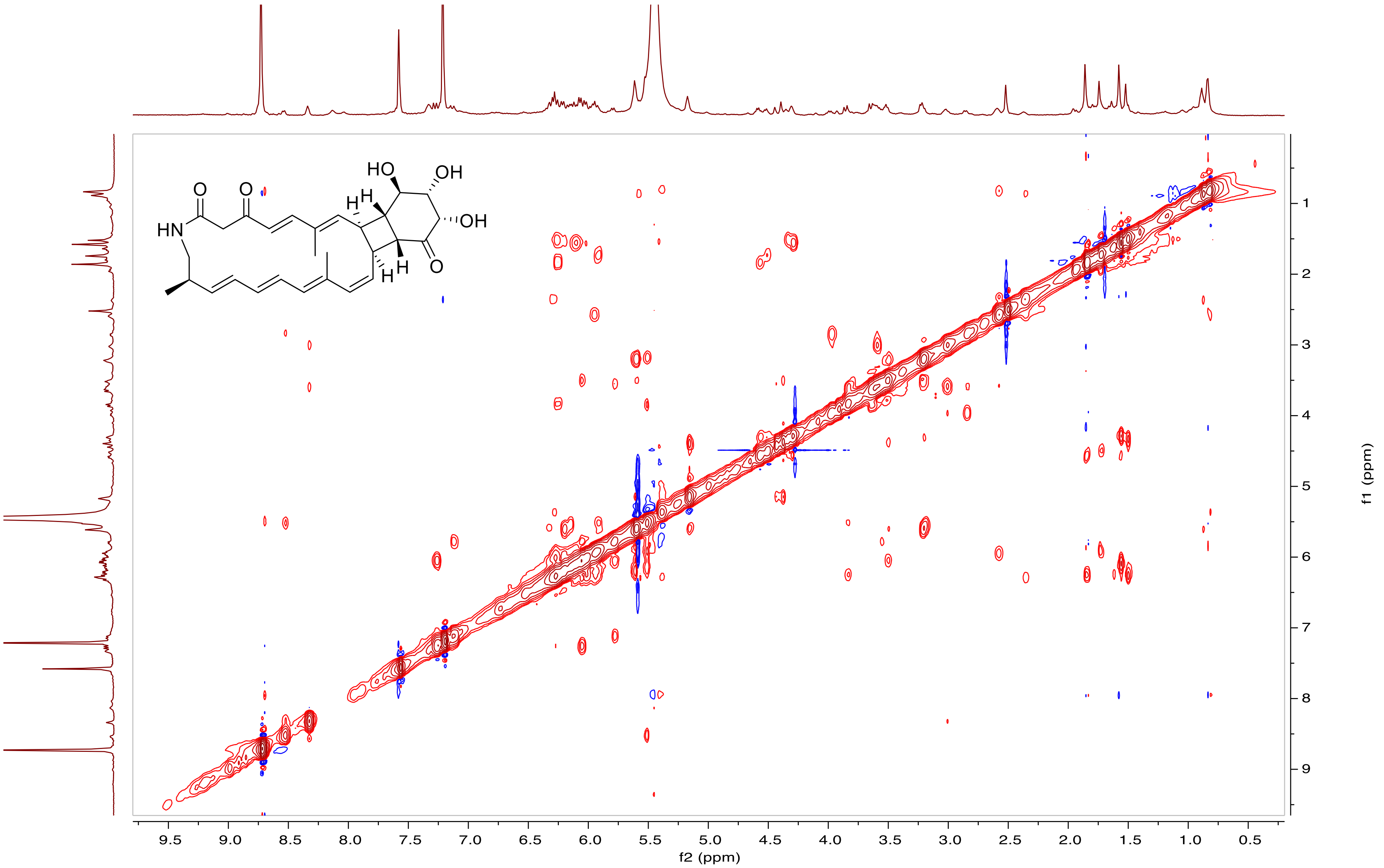

Figure S13. HRESIMS spectrum of streptolactam B (**2**)

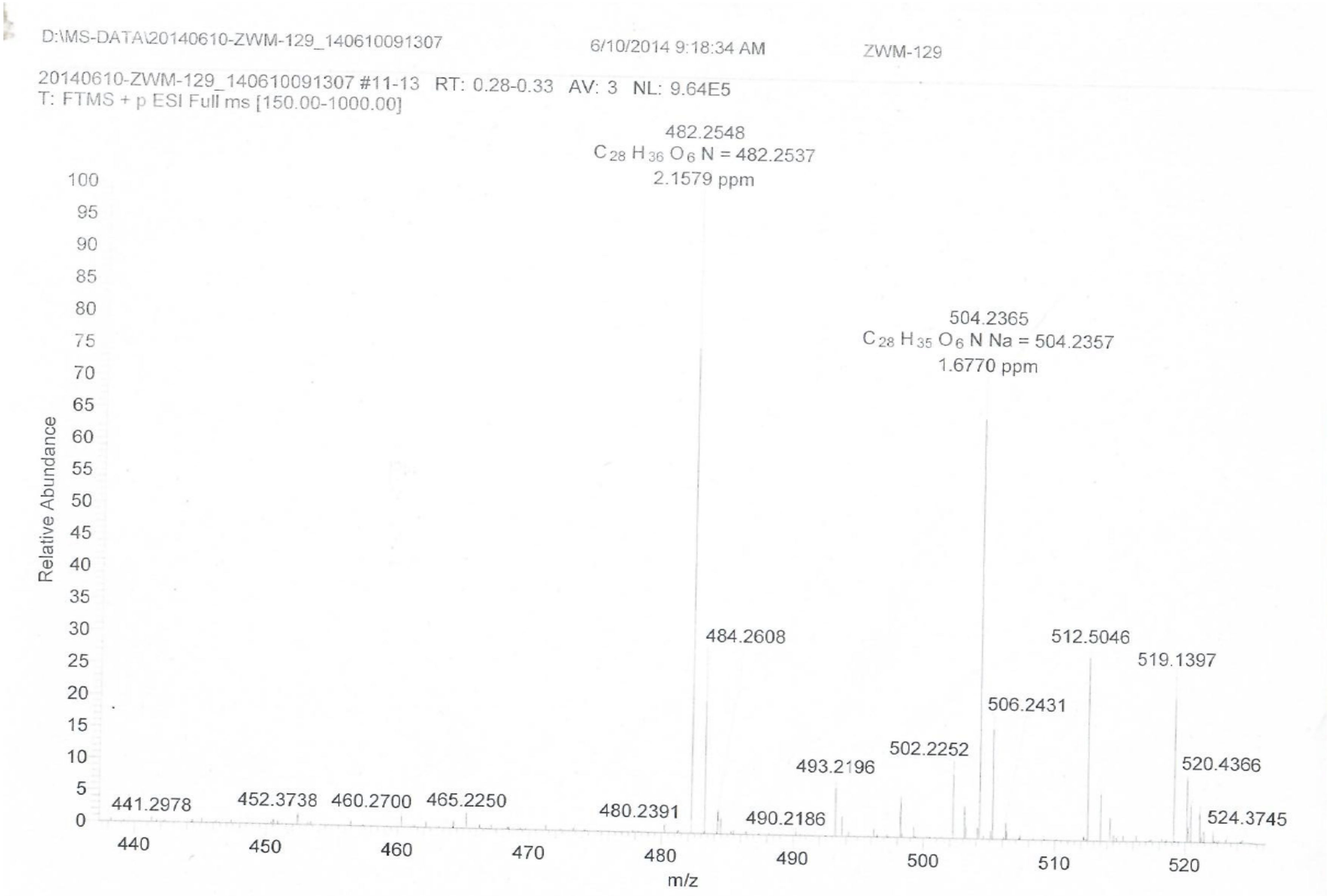

Figure S14. ^{1}H-NMR spectrum (600 MHz) of streptolactam C (**3**) in DMSO-d_6

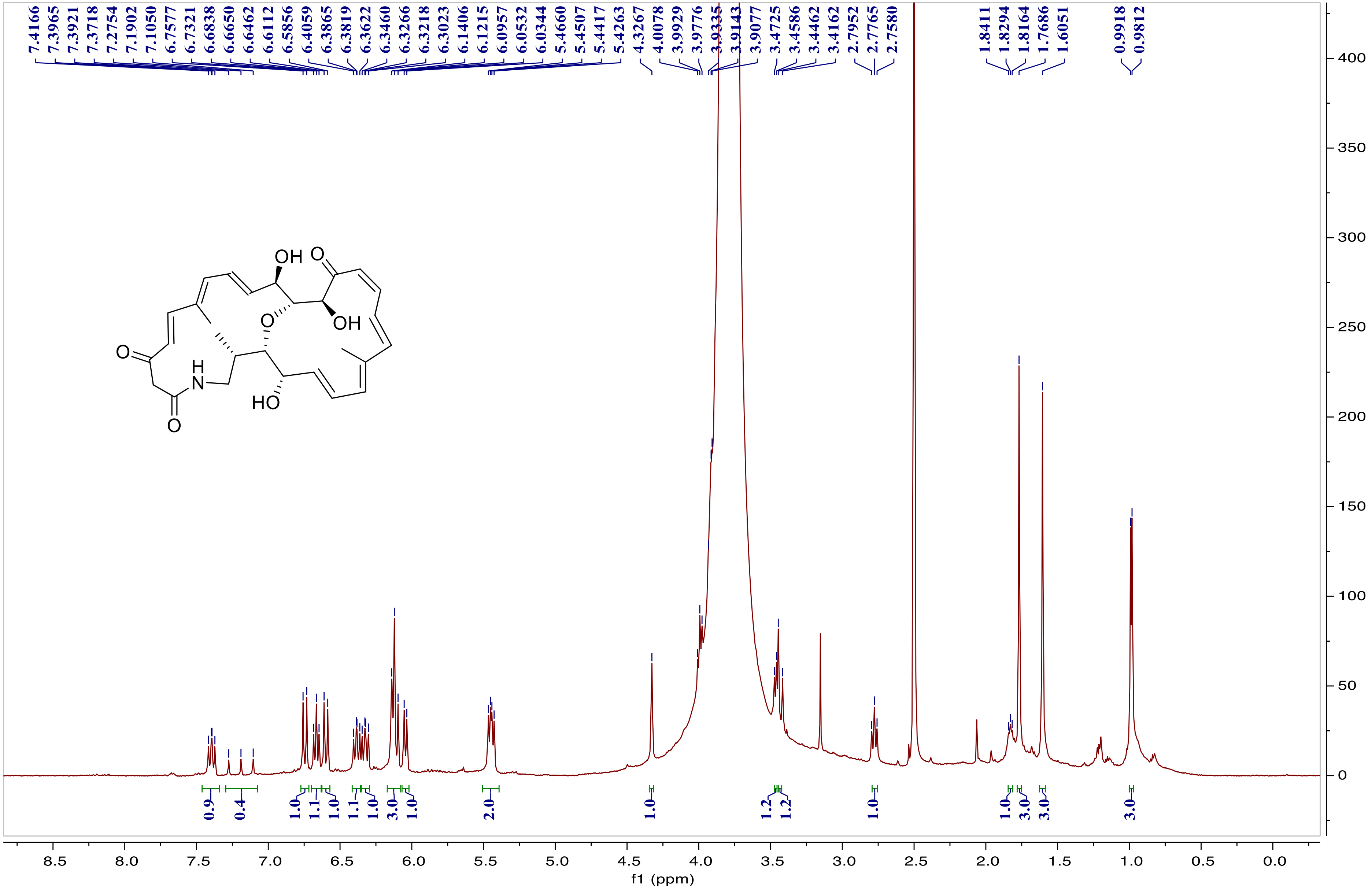

Figure S15. DEPTQ-NMR spectrum (150 MHz) of streptolactam C (**3**) in DMSO-d_6

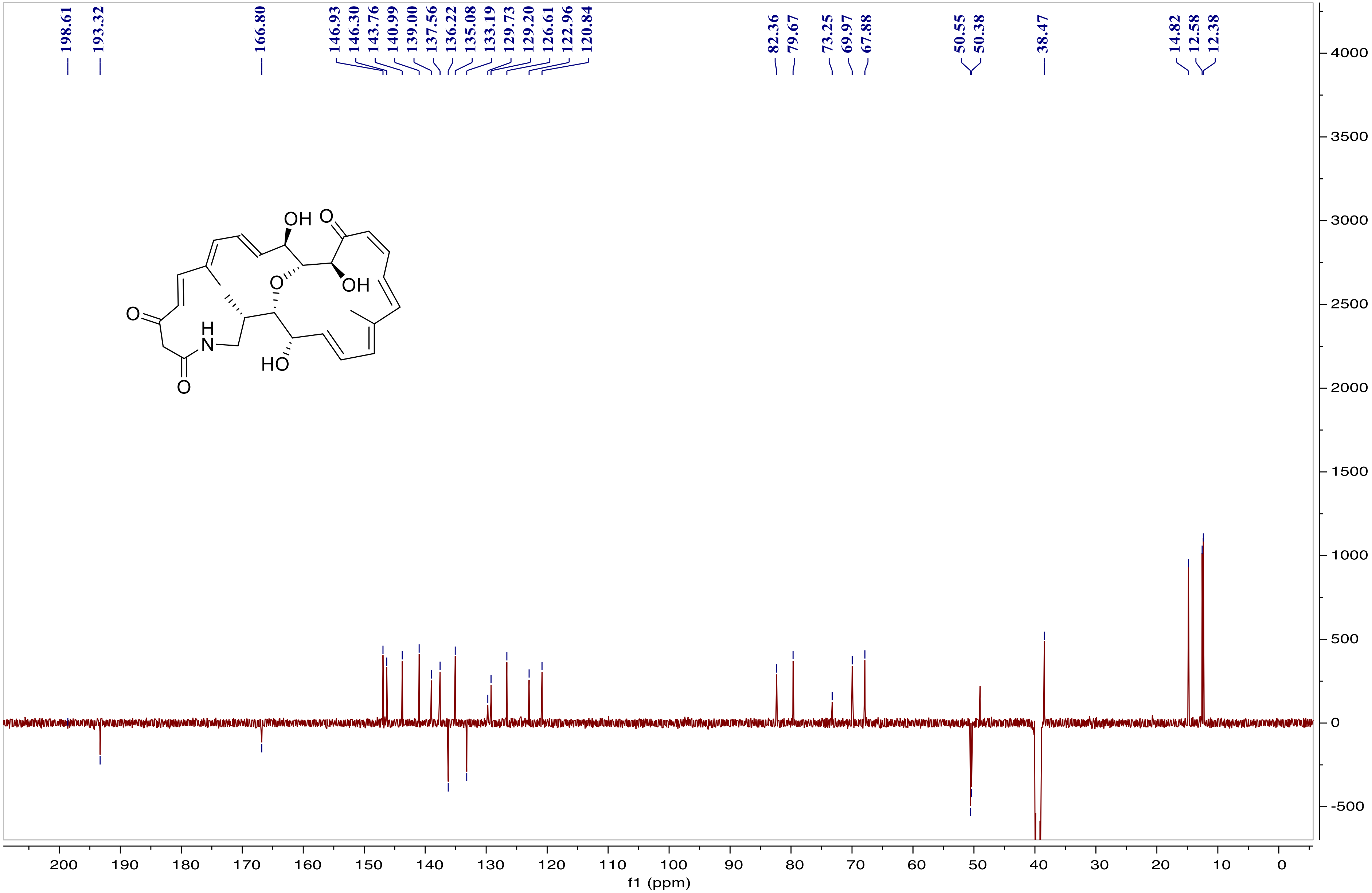

Figure S16. HSQC spectrum (600 × 150 MHz) of streptolactam C (**3**) in DMSO-*d*6

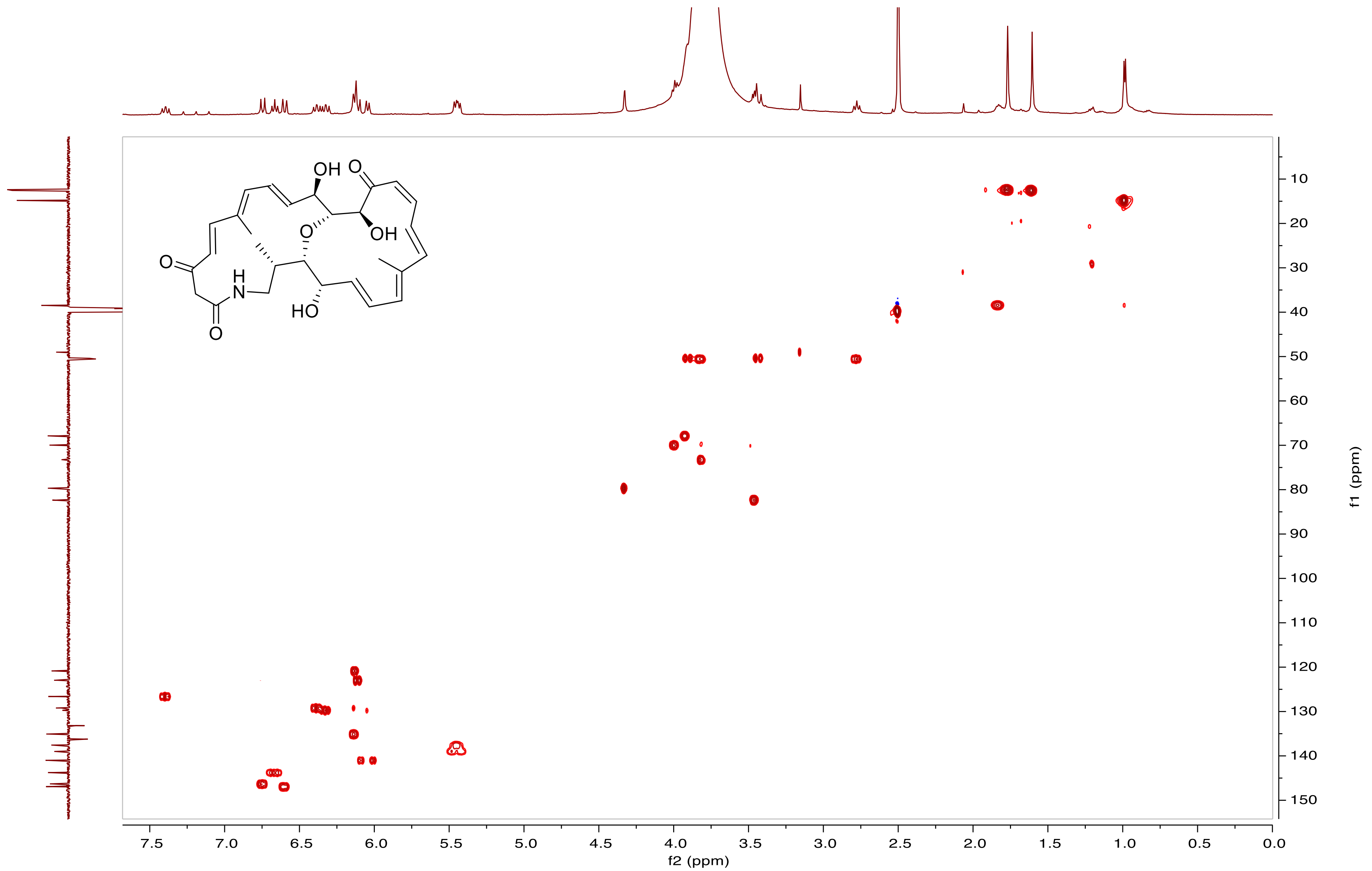

Figure S17. ^{1}H-^{1}H COSY spectrum (600 MHz) of streptolactam C (**3**) in DMSO-*d*6

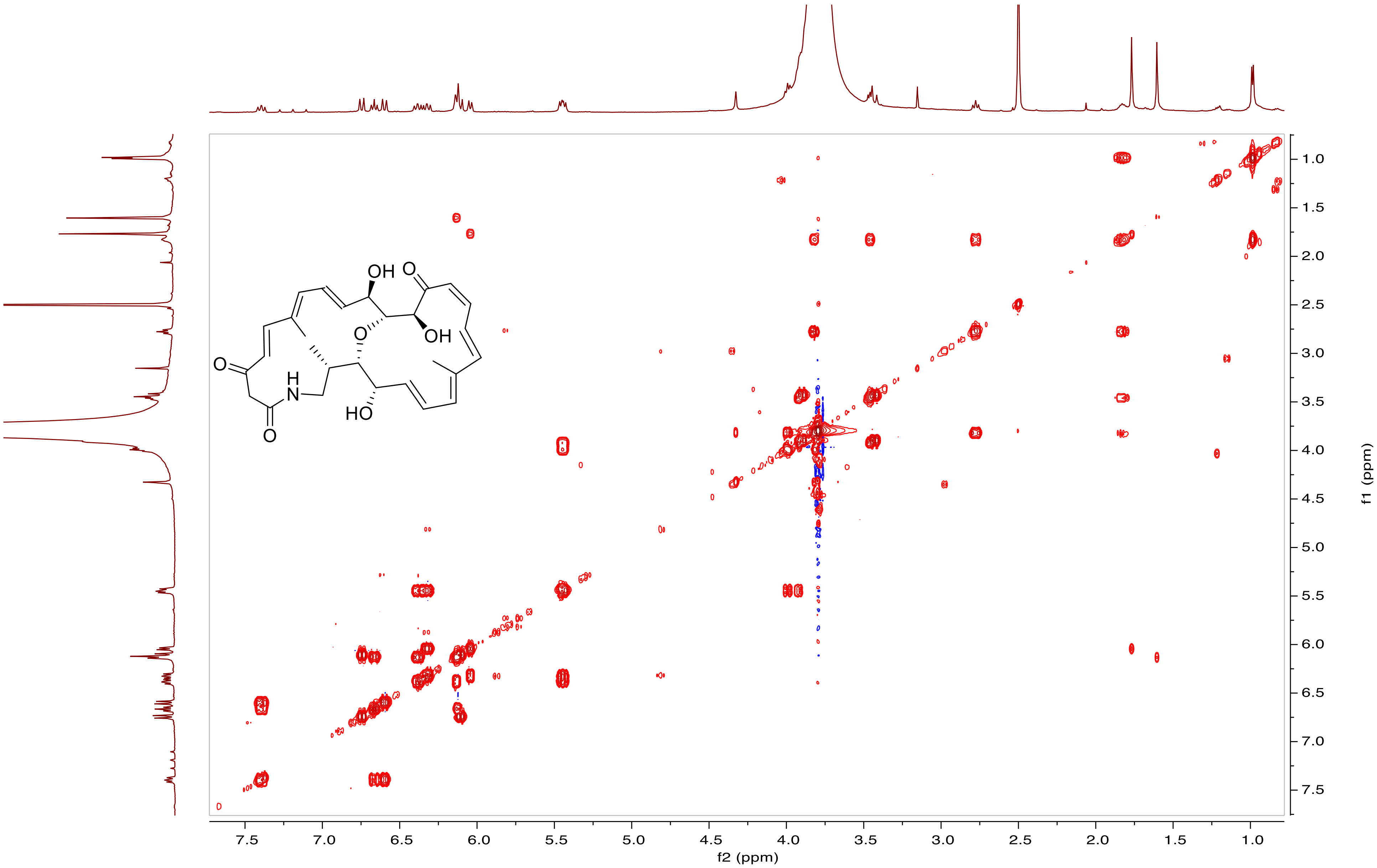

Figure S18. HMBC spectrum (600 × 150 MHz) of streptolactam C (**3**) in DMSO-d_6

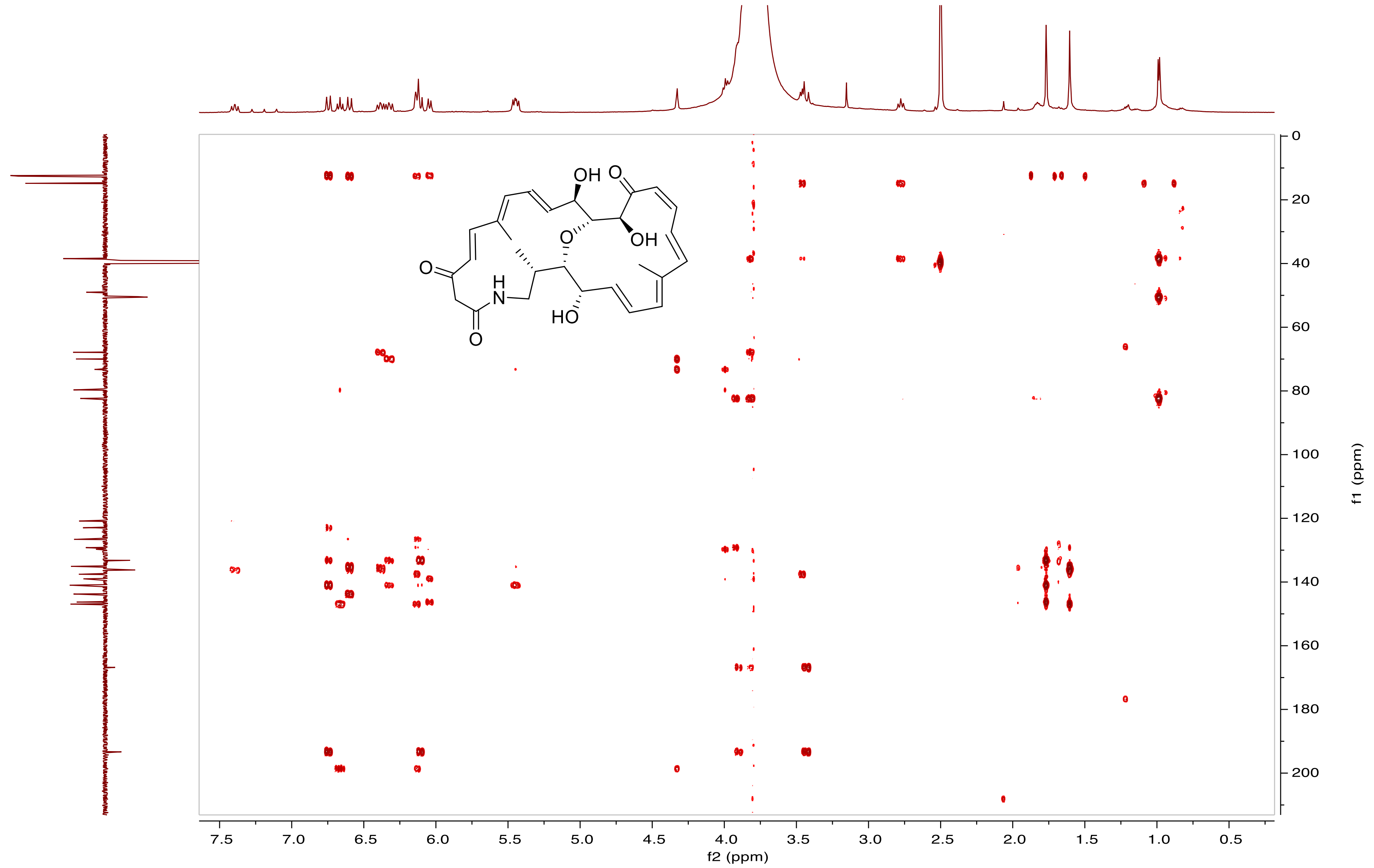

Figure S19. Partial enlarged HMBC spectrum of streptolactam C (**3**)

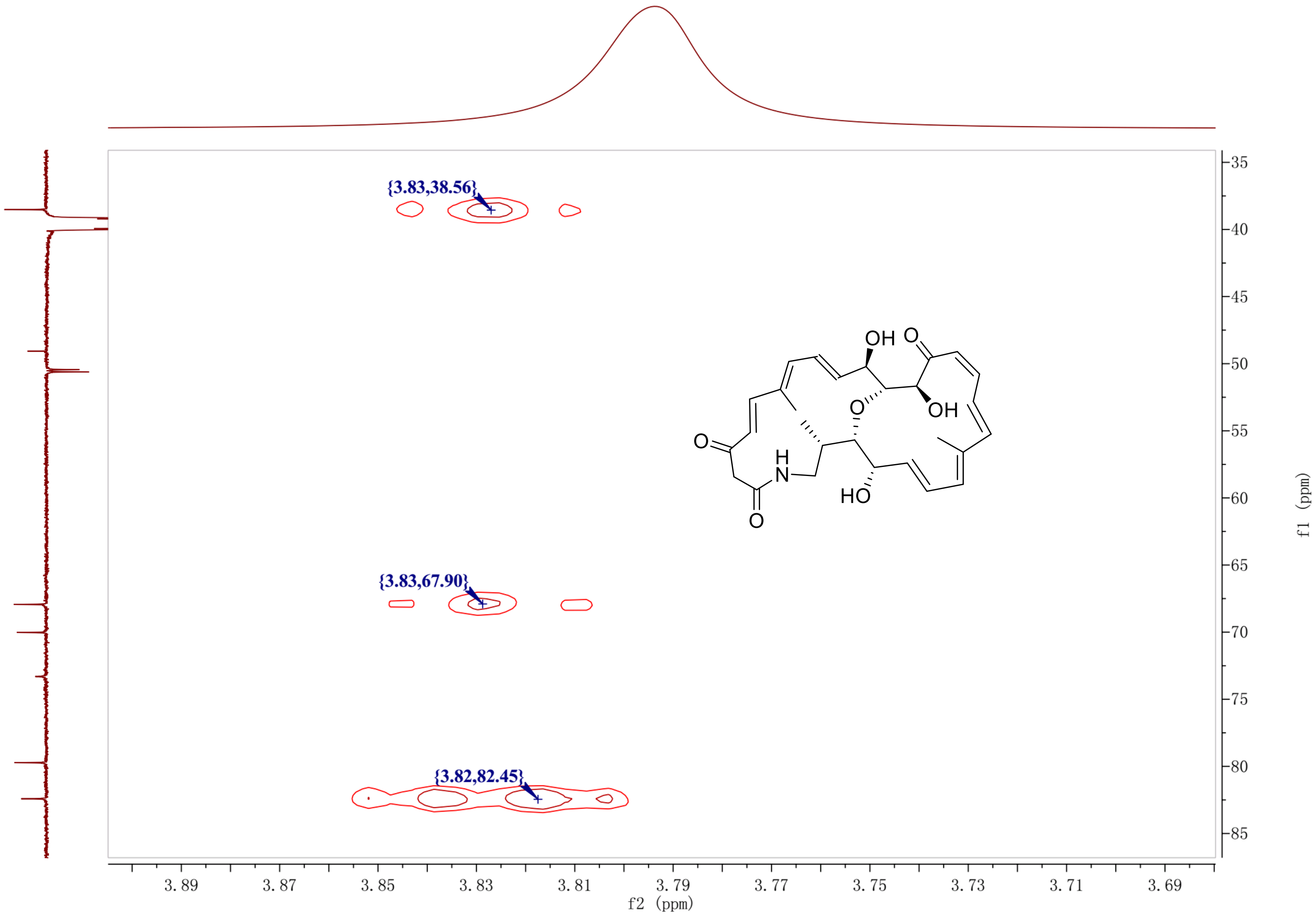

Figure S20. HRESIMS spectrum of streptolactam C (**3**)

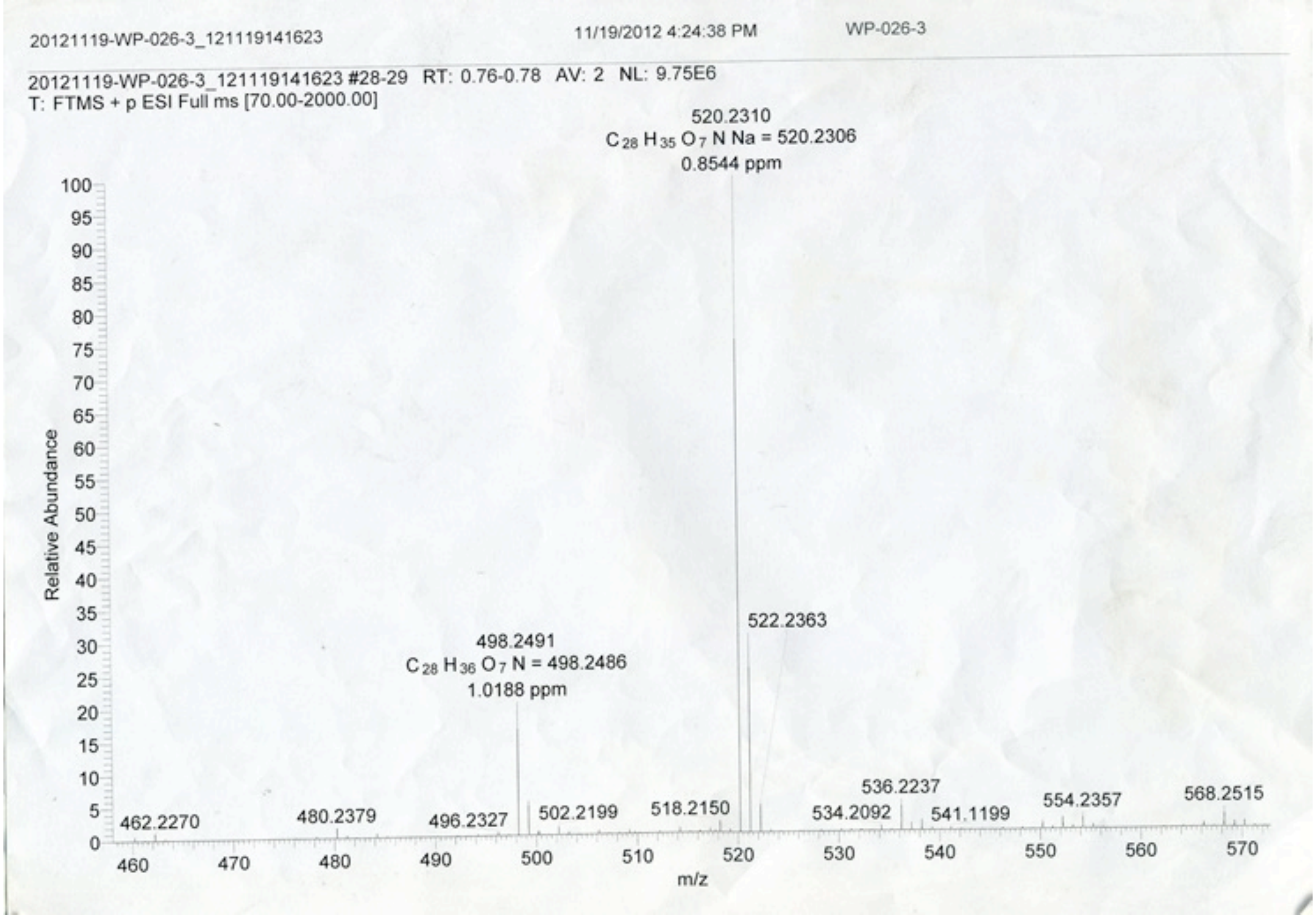

Figure S21. ^{1}H-NMR spectrum (600 MHz) of niizalactam C (**4**) in DMSO-d_6

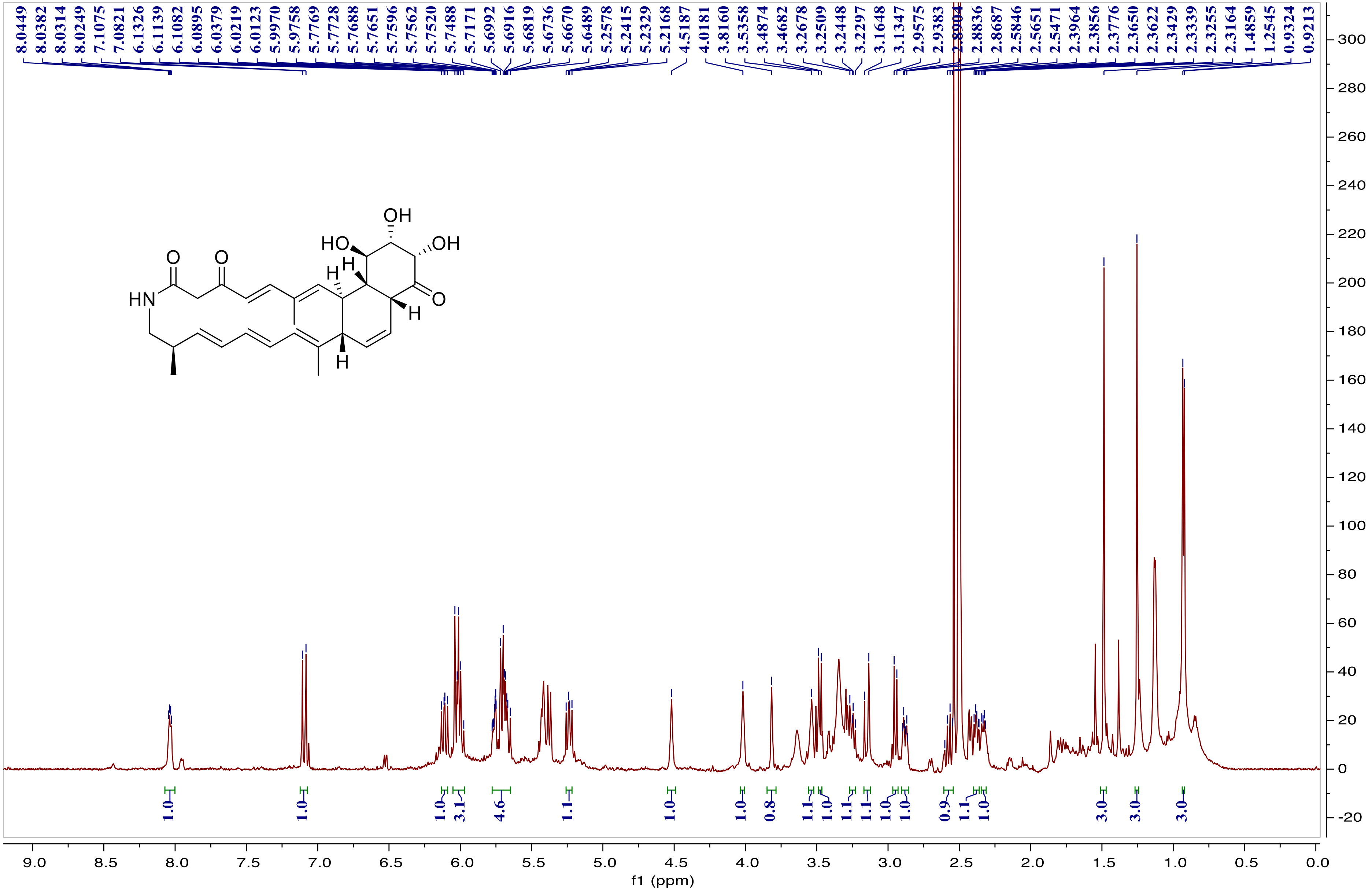

Figure S22. DEPTQ-NMR spectrum (150 MHz) of niizalactam C (**4**) in DMSO-*d*₆

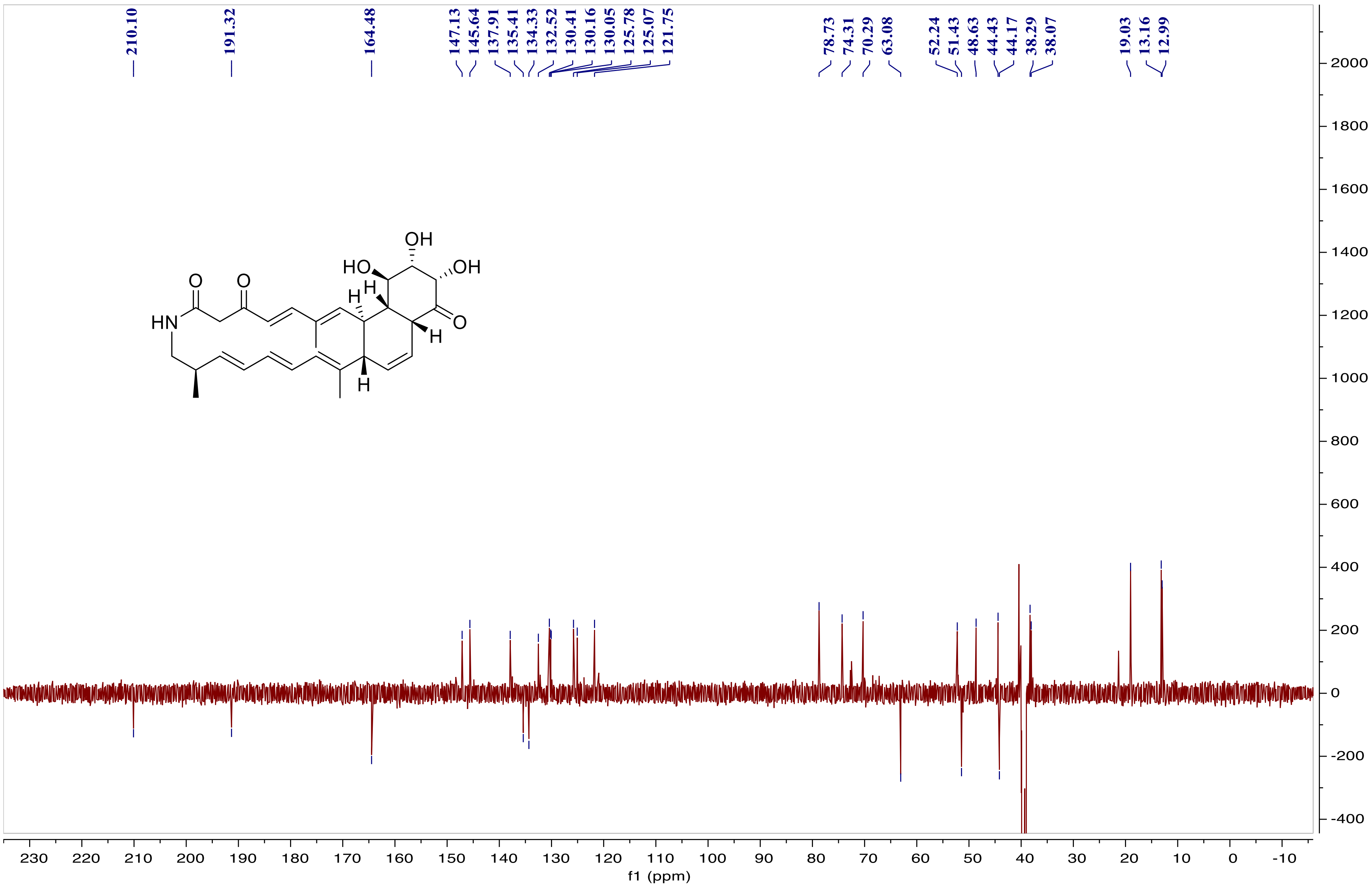

Figure S23. HSQC spectrum (600 × 150 MHz) of niizalactam C (**4**) in DMSO-d_6

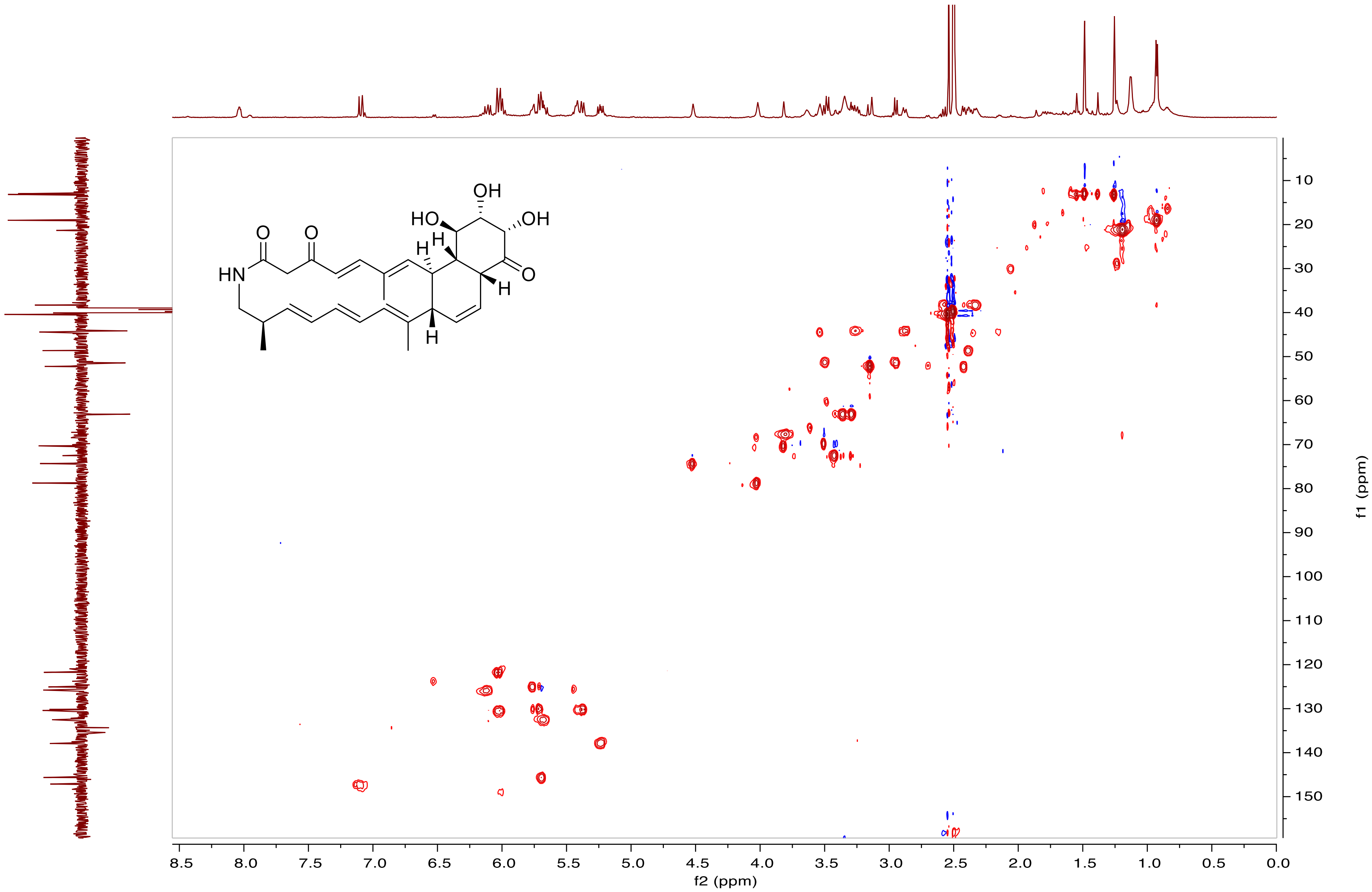

Figure S24. ^{1}H-^{1}H COSY spectrum (600 MHz) of niizalactam C (**4**) in DMSO-d_6

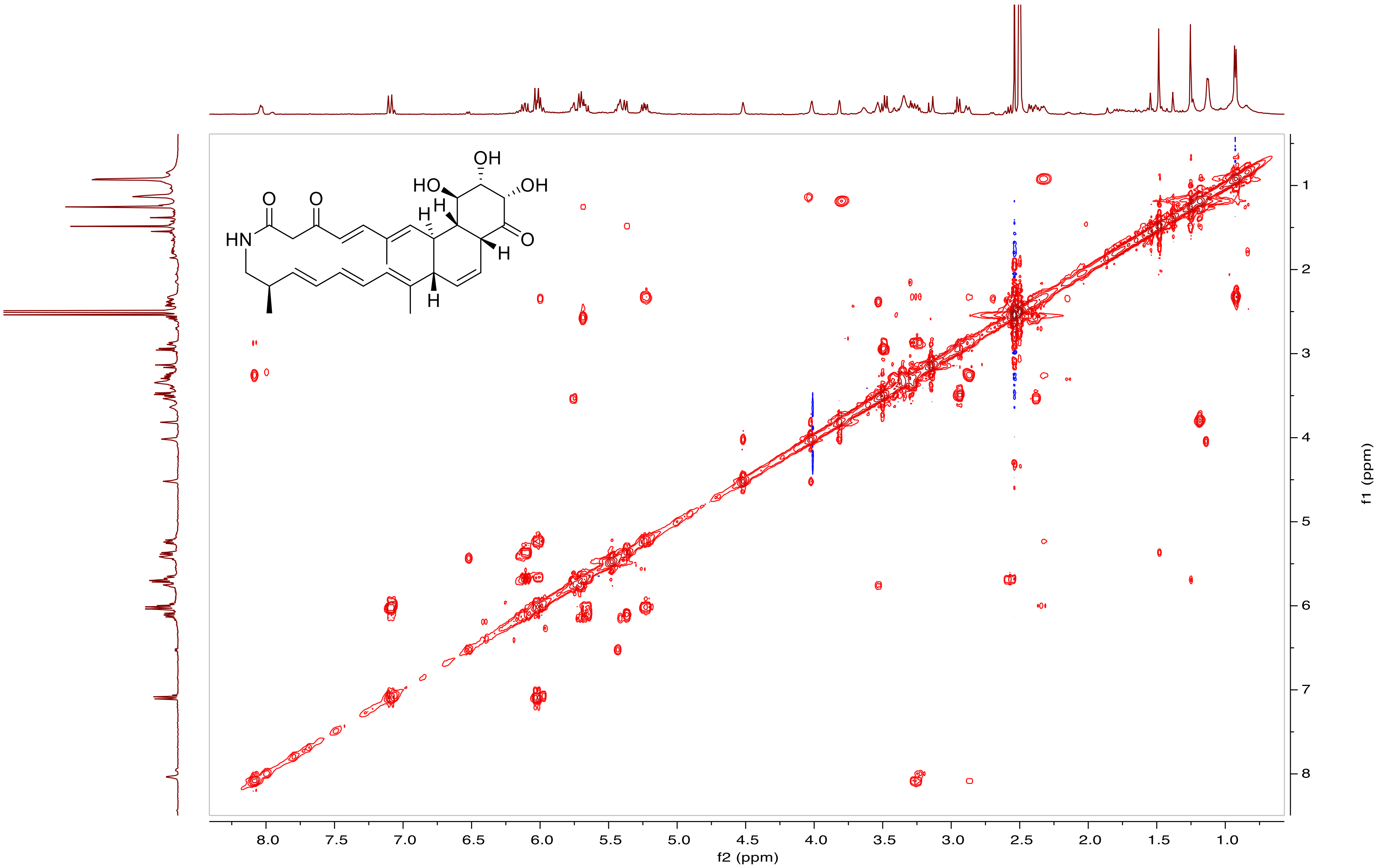

Figure S25. HMBC spectrum (600 × 150 MHz) of niizalactam C (**4**) in DMSO-*d*₆

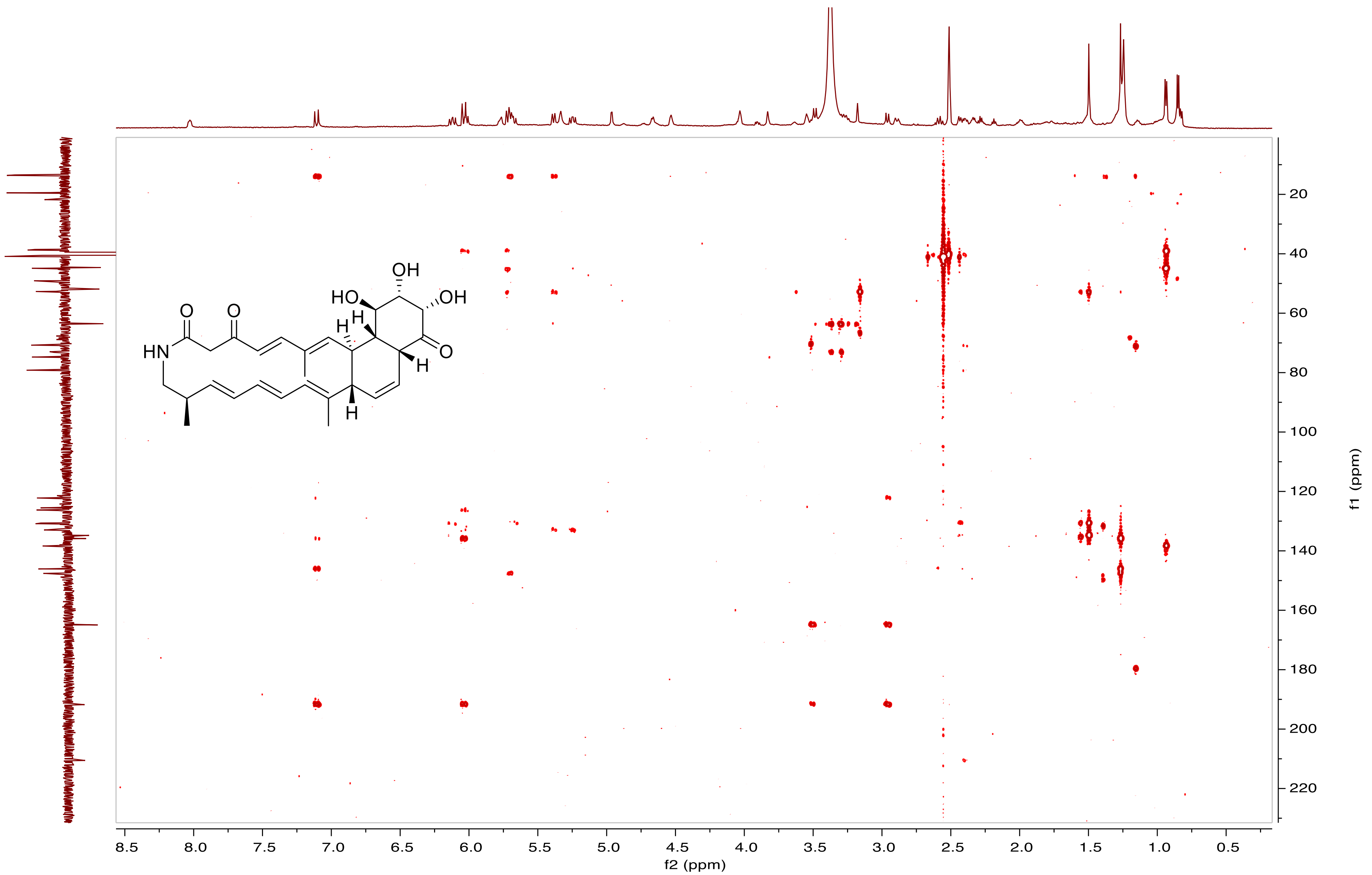

Figure S26. NOESY spectrum (600 MHz) of niizalactam C (**4**) in DMSO-d_6

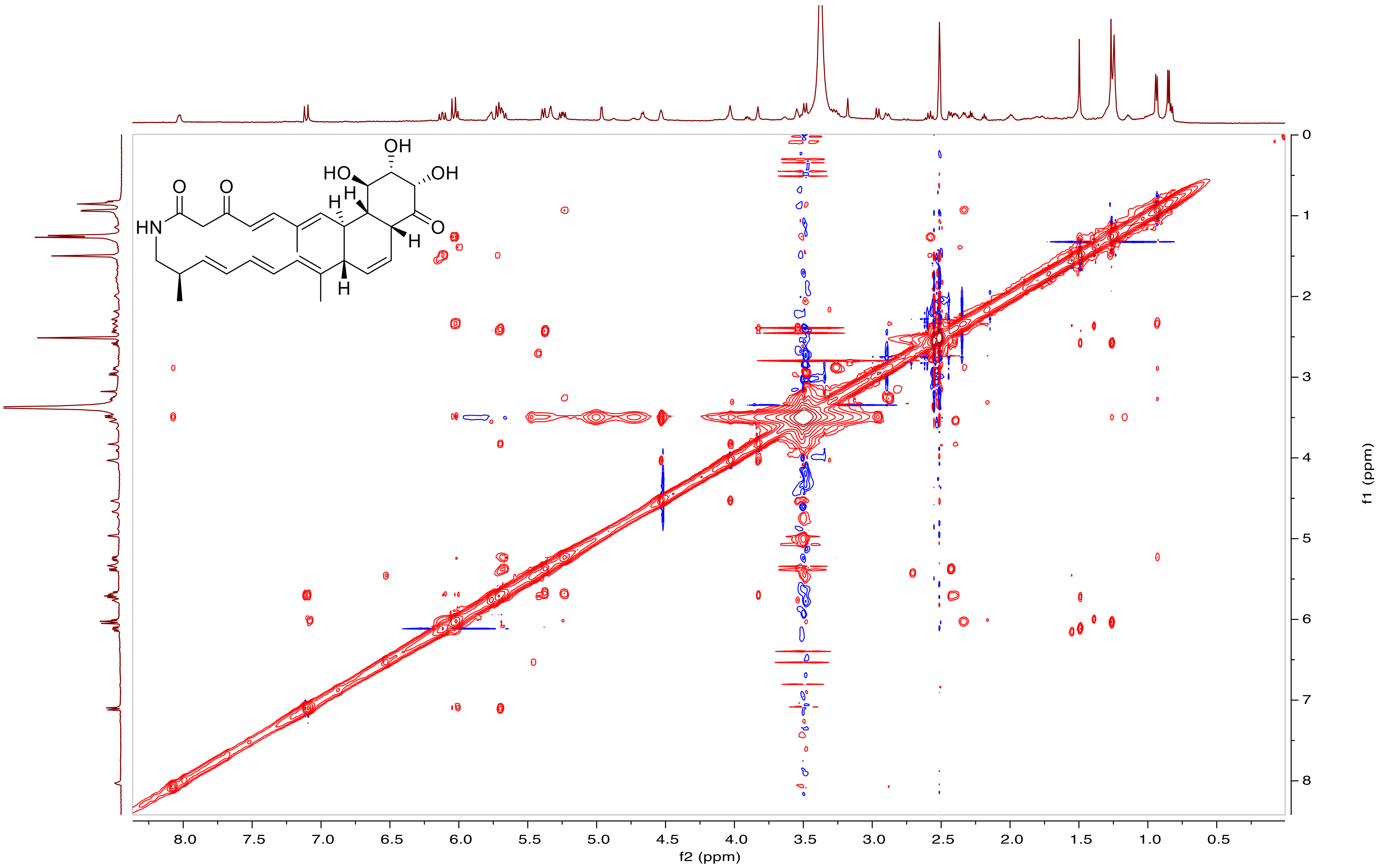

Figure S27. HRESIMS spectrum of niizalactam C (**4**)

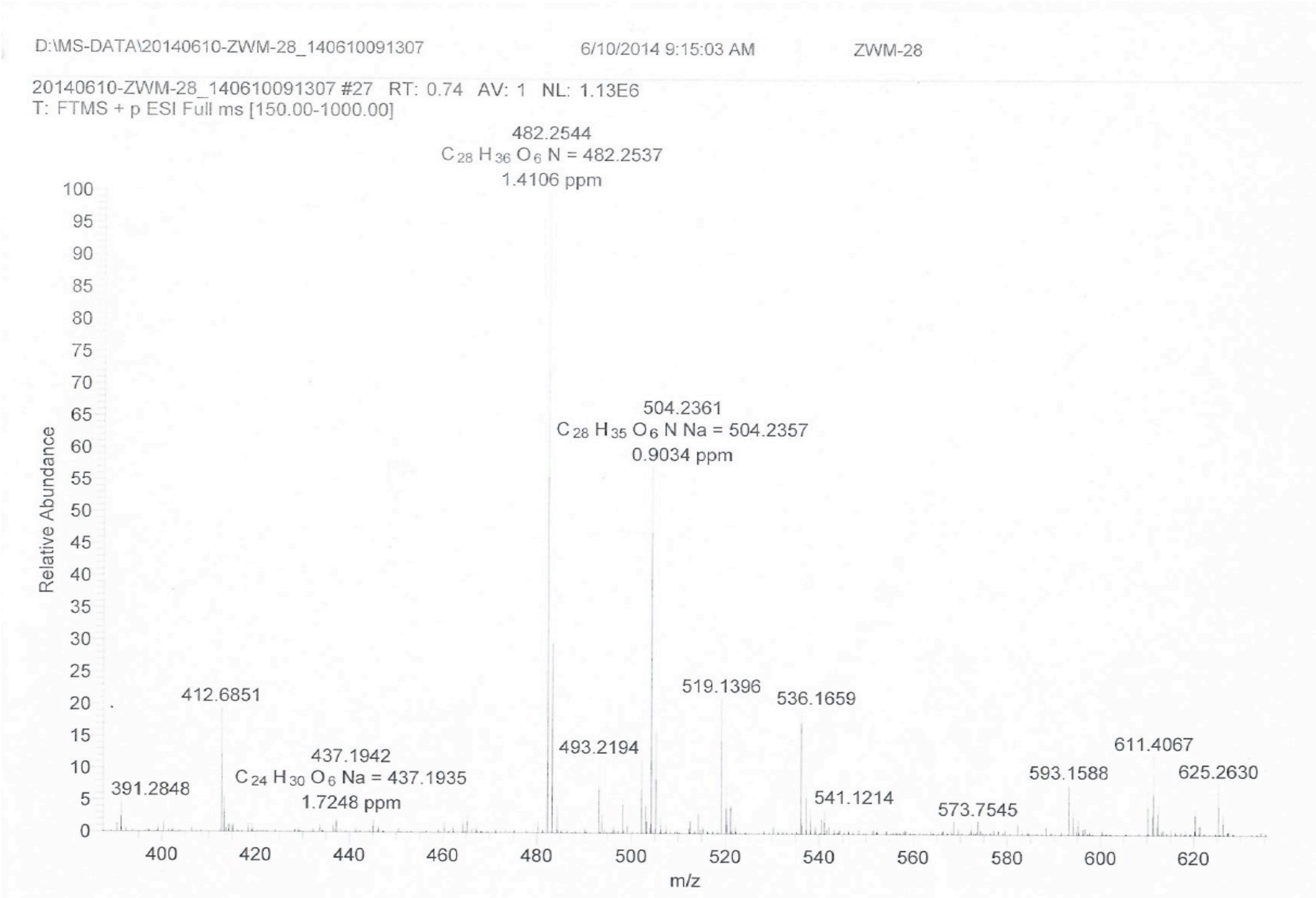

Figure S28. ^{1}H-NMR spectrum (600 MHz) of compound **1a** in acetone-d_6

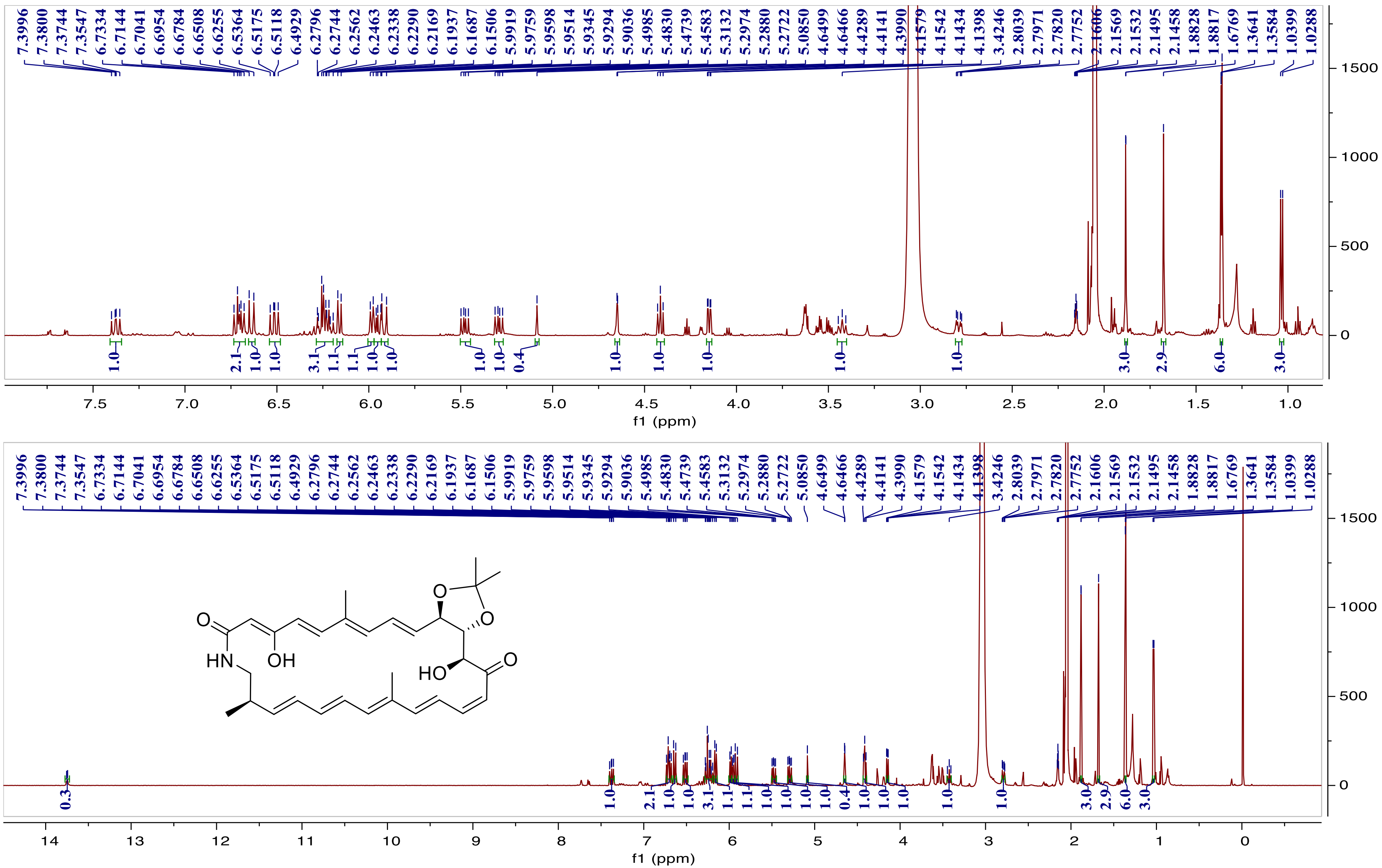

Figure S29. DEPTQ-NMR spectrum (150 MHz) of compound **1a** in acetone-d_6

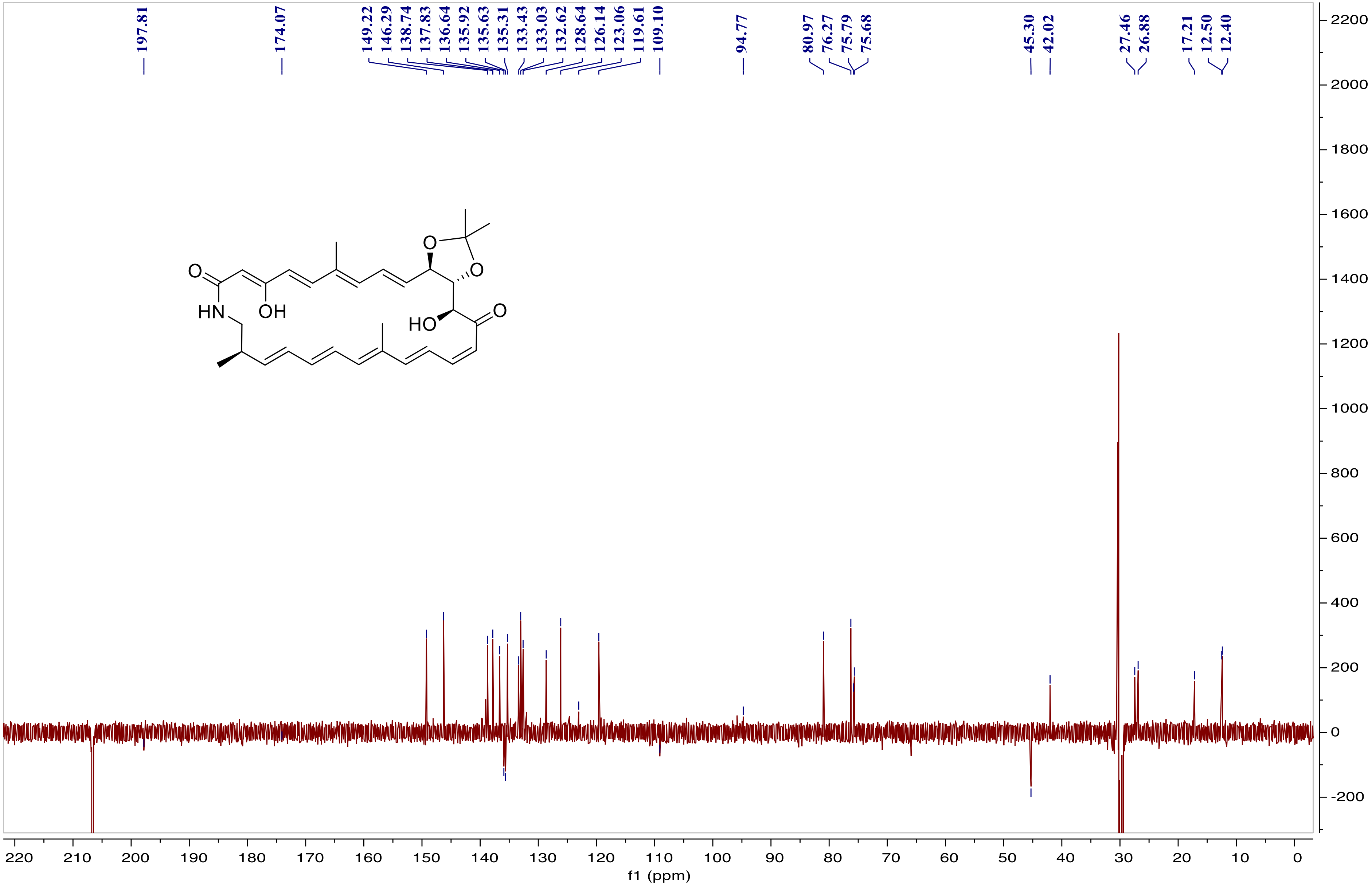

Figure S30. HSQC spectrum (600 × 150 MHz) of compound **1a** in acetone-d_6

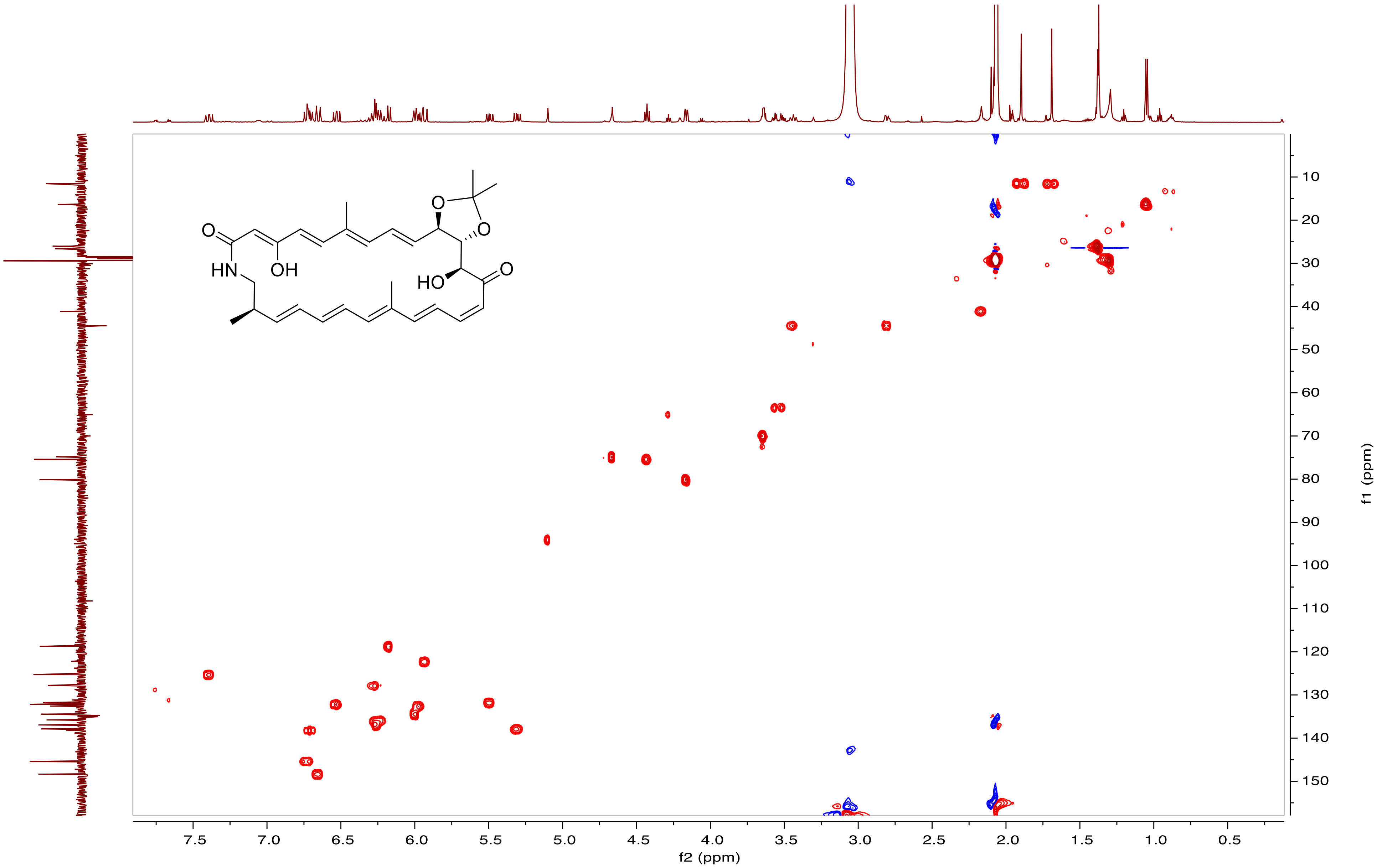

Figure S31. ^{1}H-^{1}H COSY spectrum (600 MHz) of compound **1a** in acetone-d_6

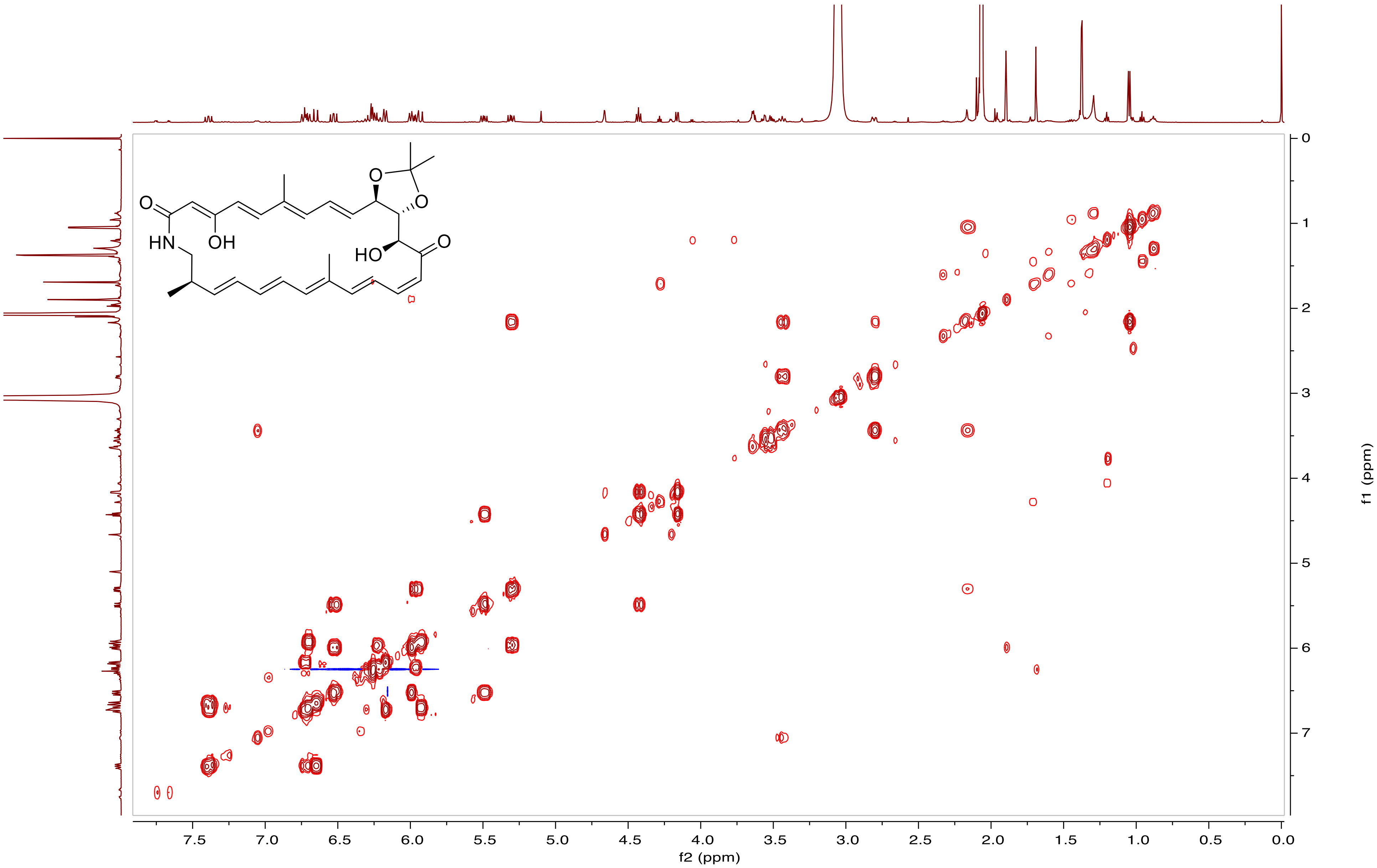

Figure S32. HMBC spectrum (600 × 150 MHz) of compound **1a** in acetone-d_6

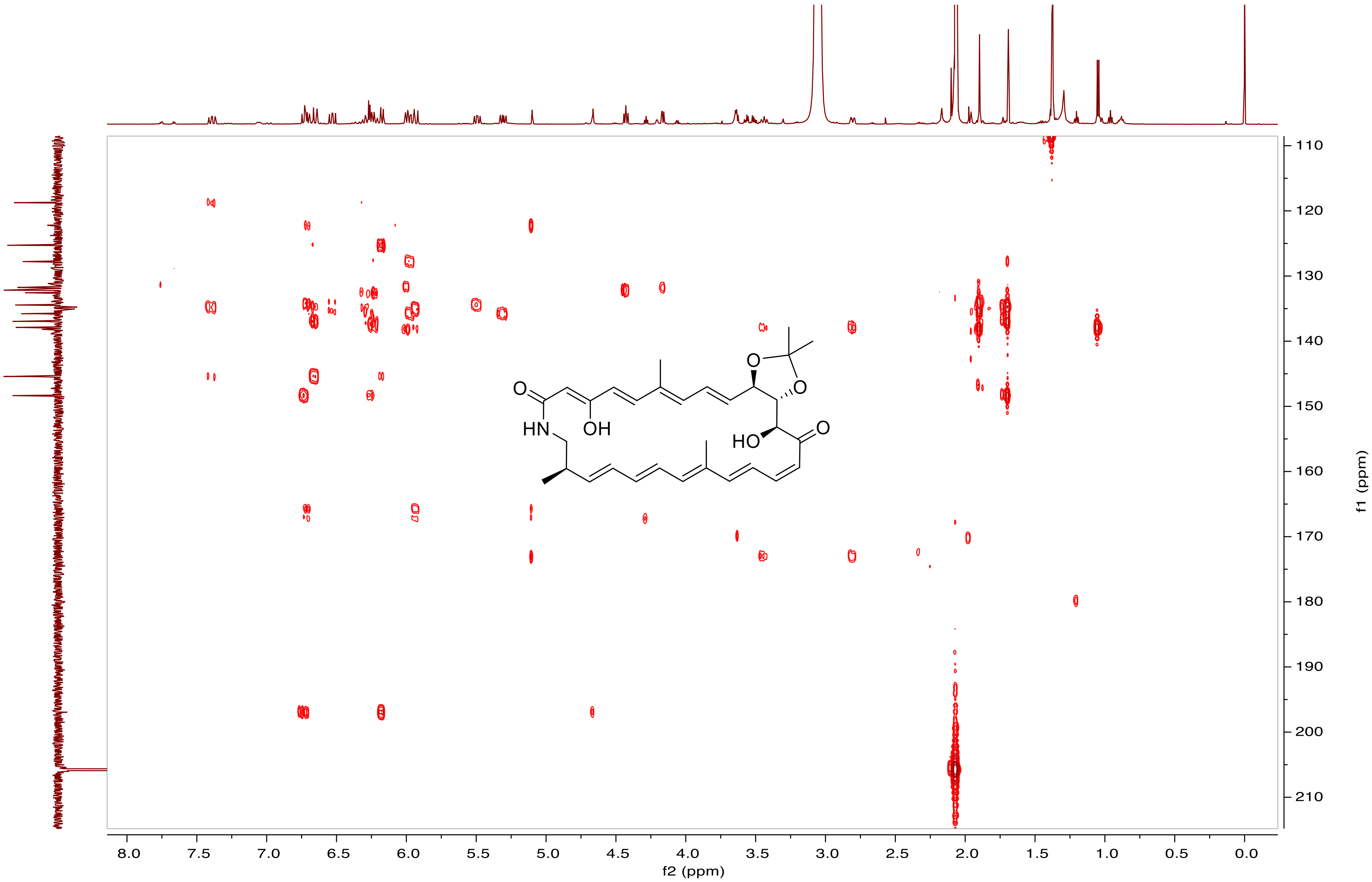

Figure S33. NOESY spectrum (600 MHz) of compound **1a** in acetone-d_6

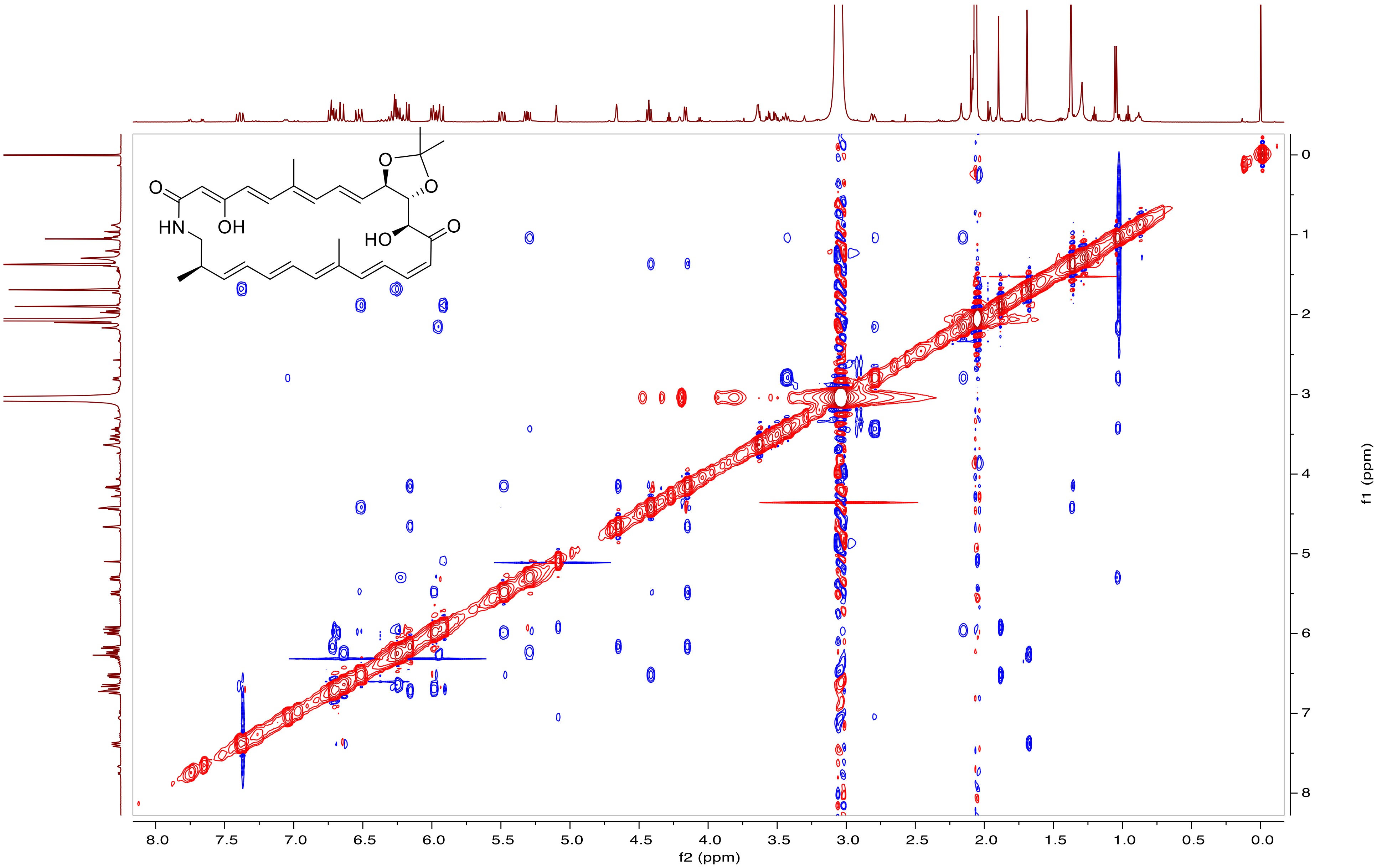

Figure S34. HRESIMS spectrum of compound **1a**

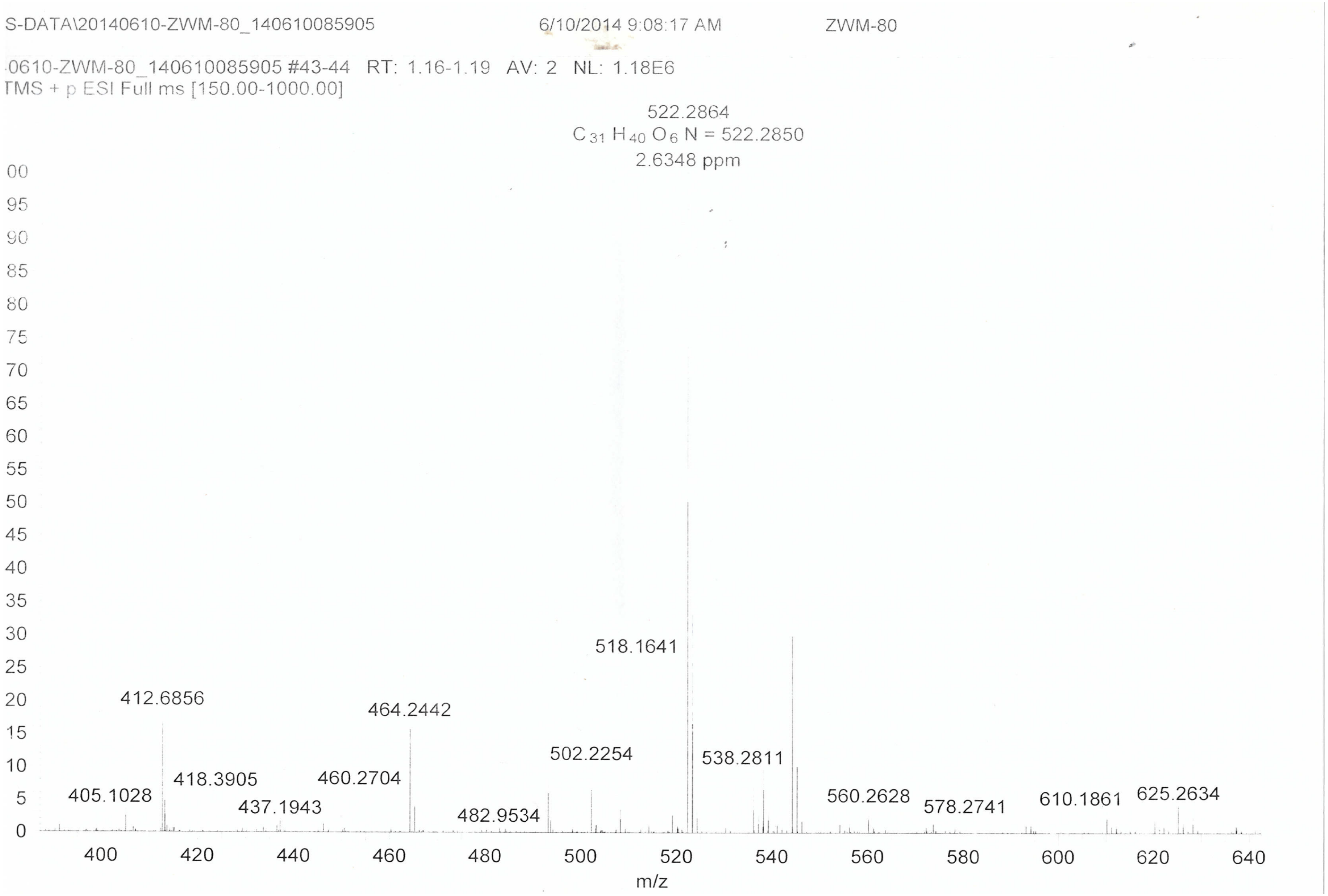

Figure S35. ^{1}H-NMR spectrum (600 MHz) of compound **1b** in $CDCl_3$

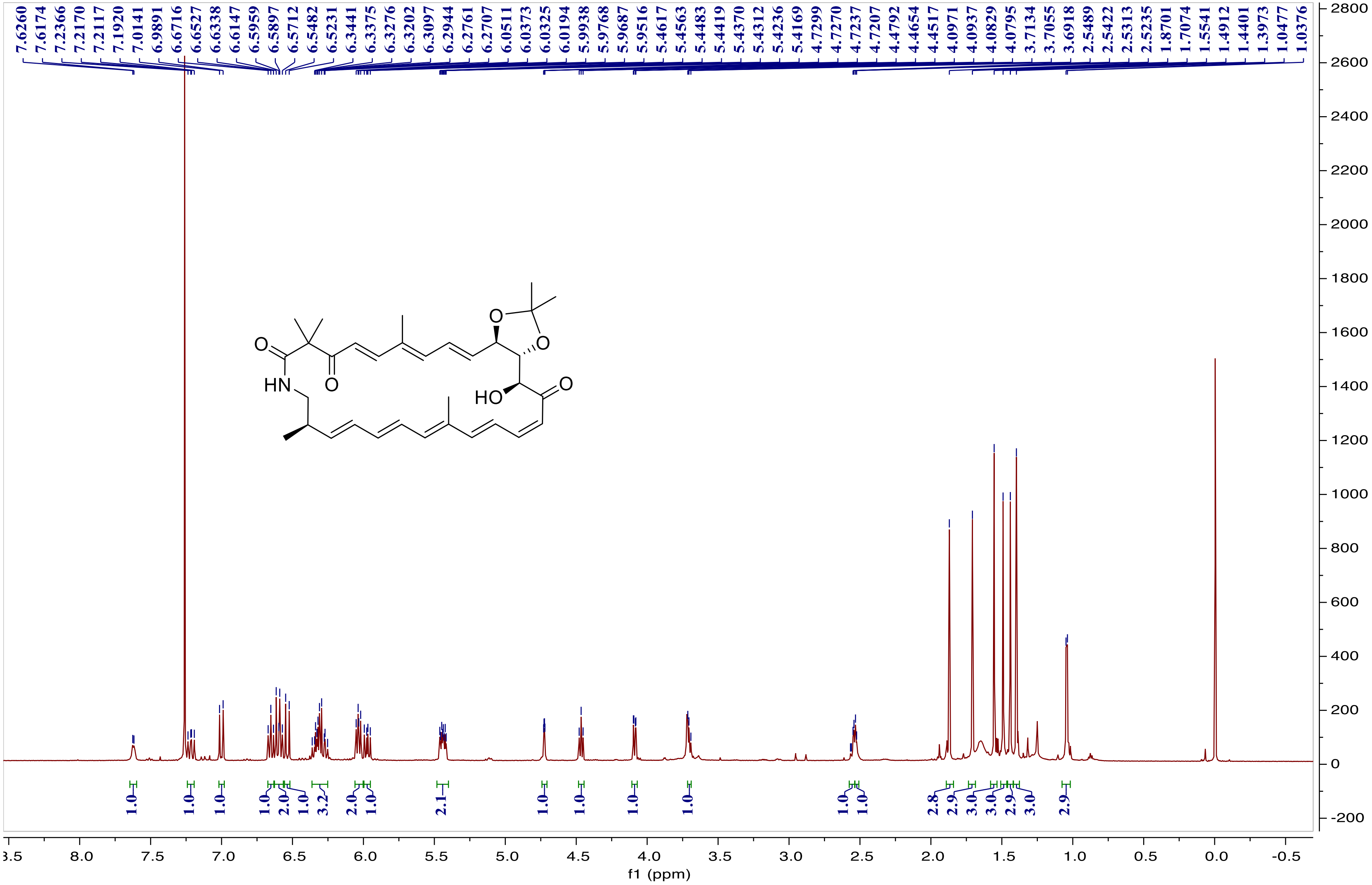

Figure S36. DEPTQ-NMR spectrum (150 MHz) of compound **1b** in $CDCl_3$

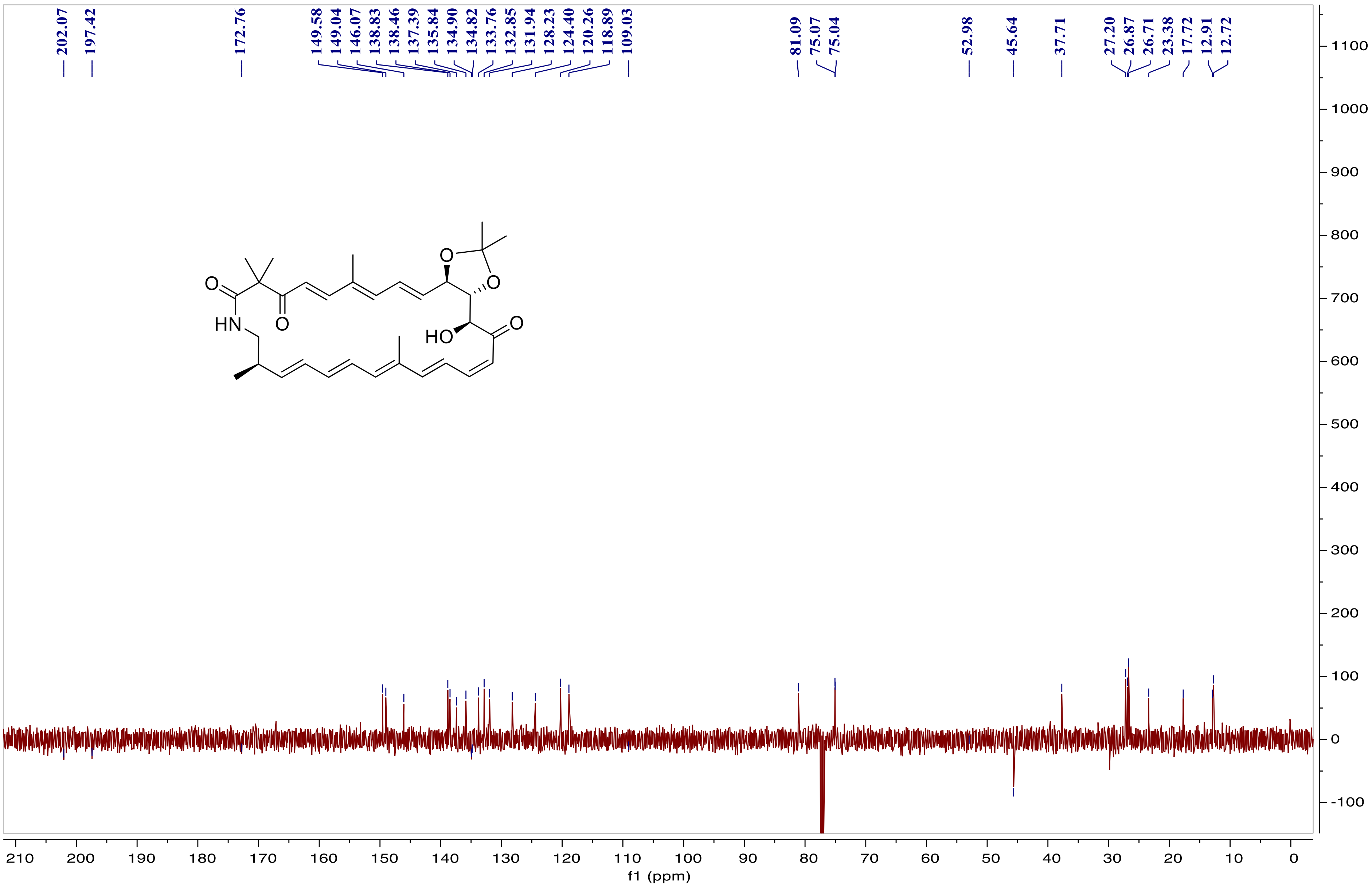

Figure S37. HSQC spectrum (600 × 150 MHz) of compound **1b** in $CDCl_3$

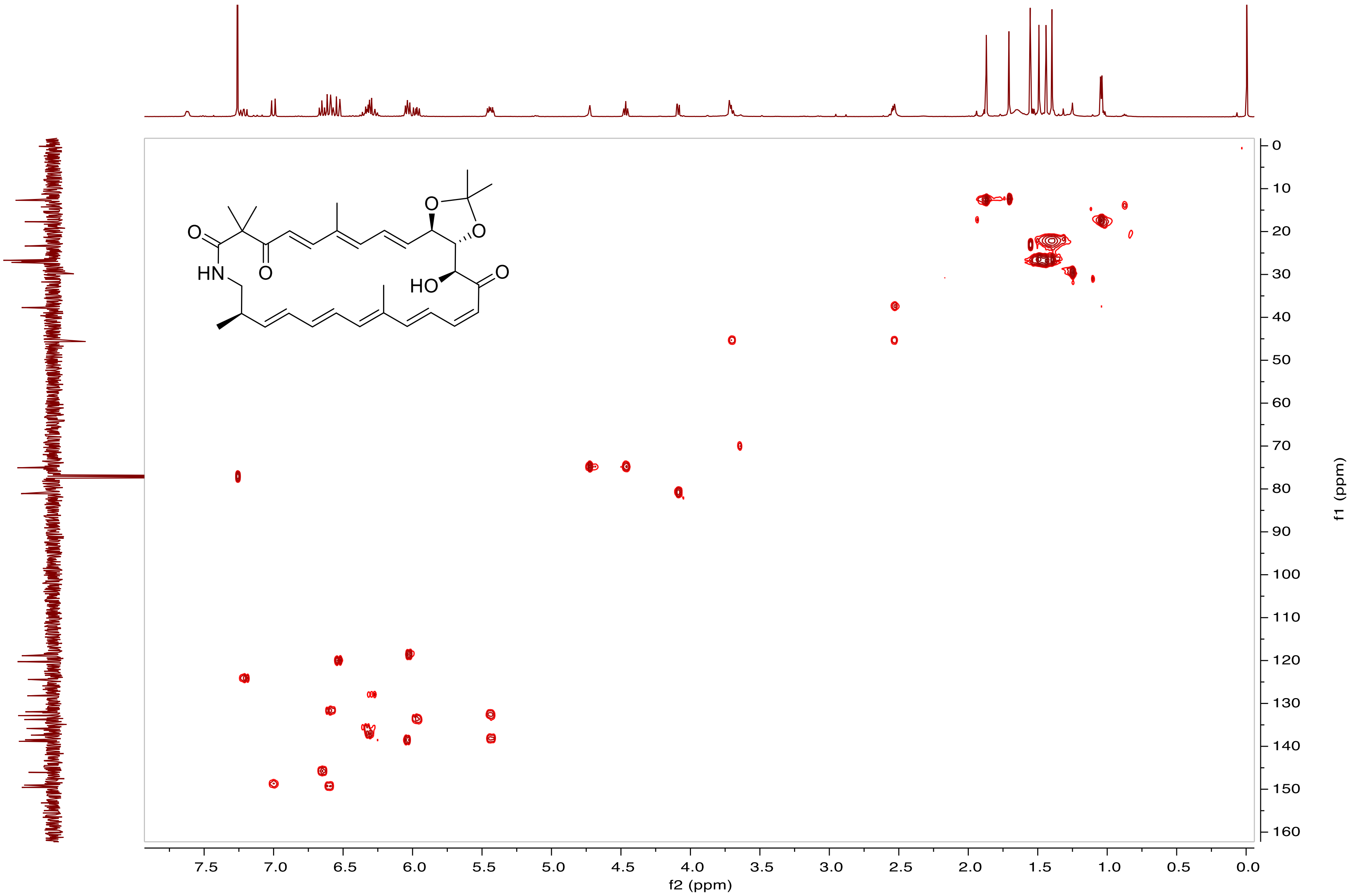

Figure S38. ^1H-^1H COSY spectrum (600 MHz) of compound **1b** in $CDCl_3$

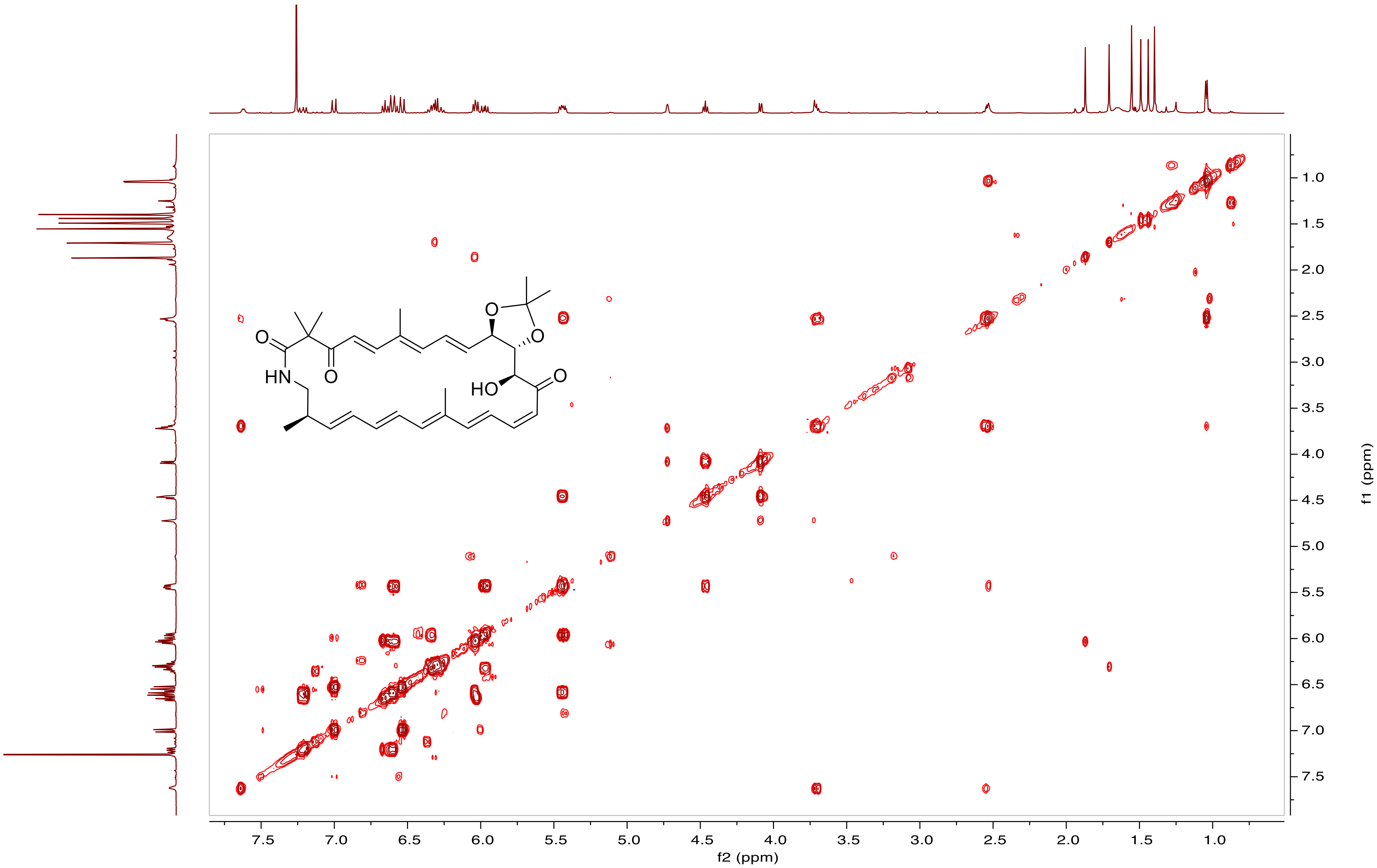

Figure S39. HMBC spectrum (600 × 150 MHz) of compound **1b** in $CDCl_3$

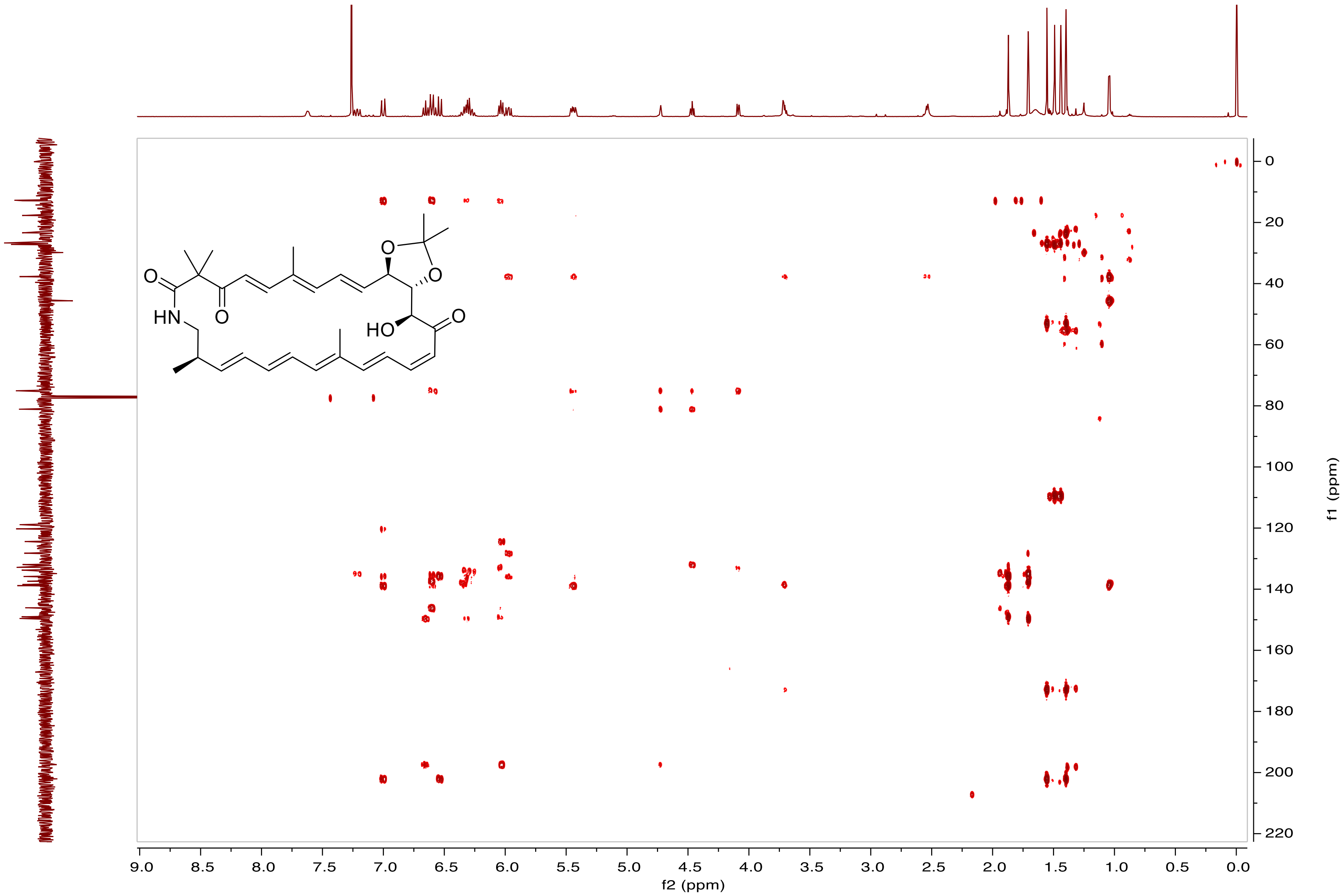

Figure S40. HRESIMS spectrum of compound **1b**

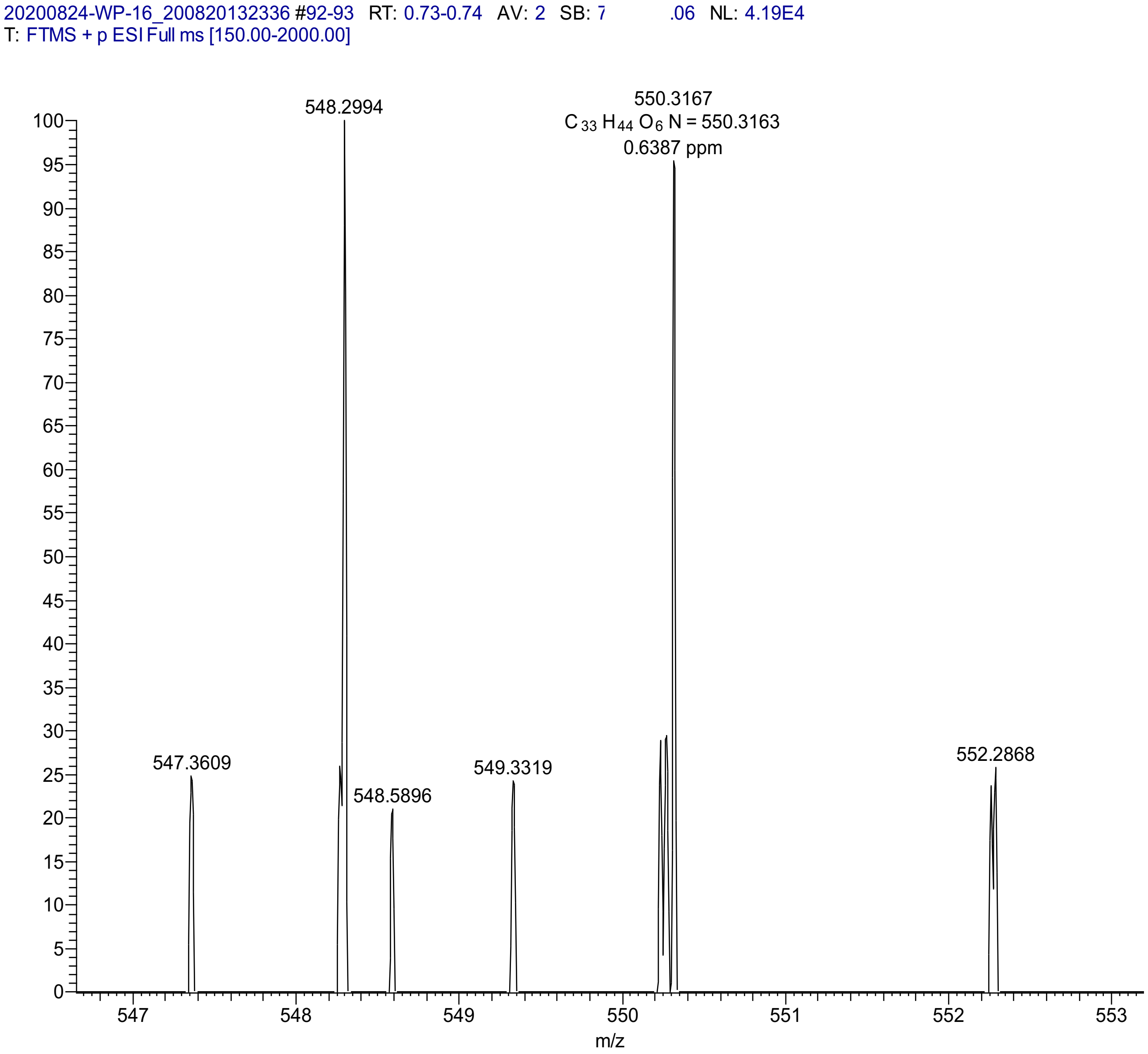

Figure S41. ^{1}H-NMR spectrum (600 MHz) of (*S*)-MTPA ester (**1ba**) of **1b** in $CDCl_3$

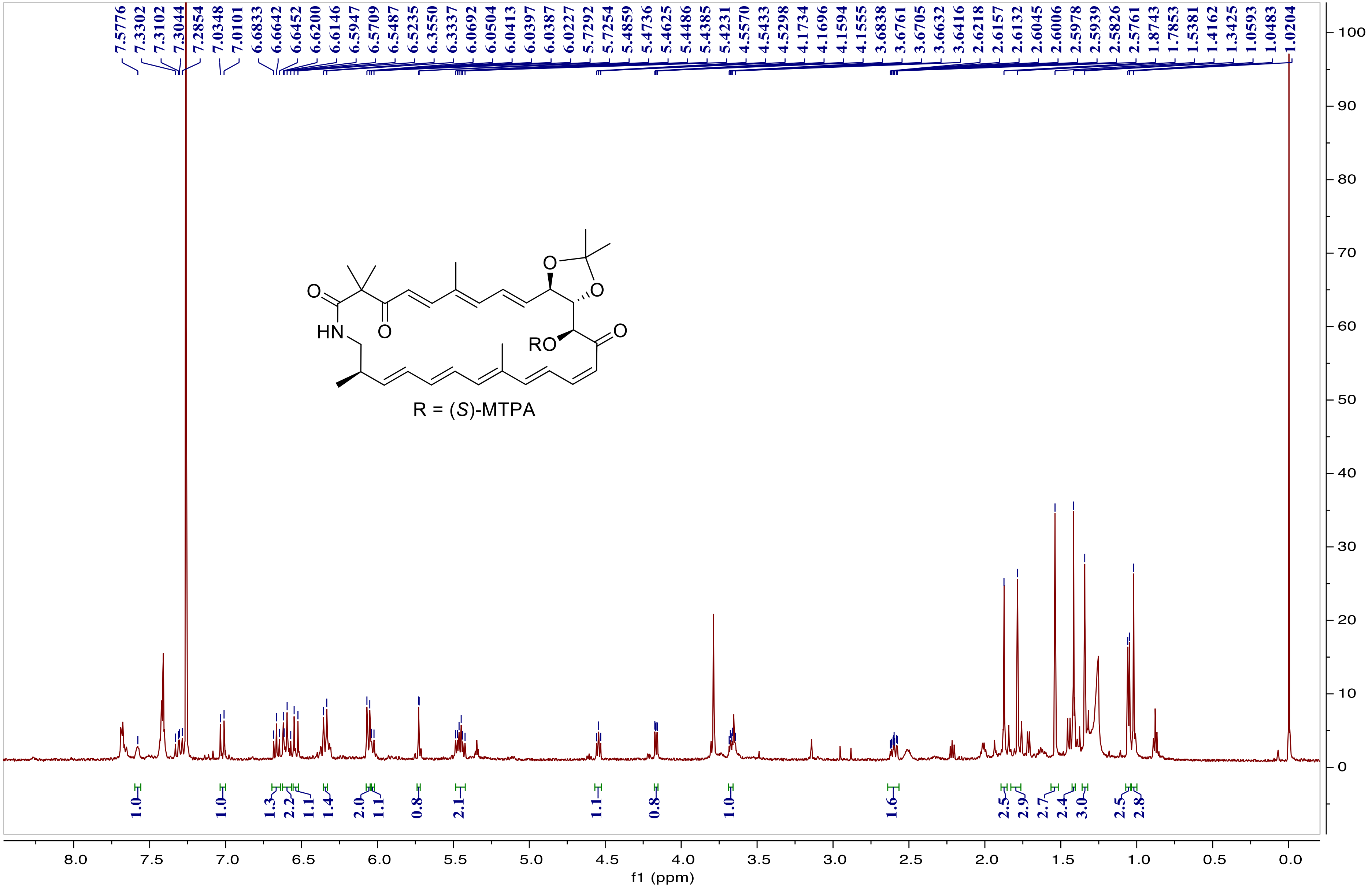

Figure S42. ^{1}H-^{1}H COSY spectrum (600 MHz) of (*S*)-MTPA ester (**1ba**) of **1b** in $CDCl_3$

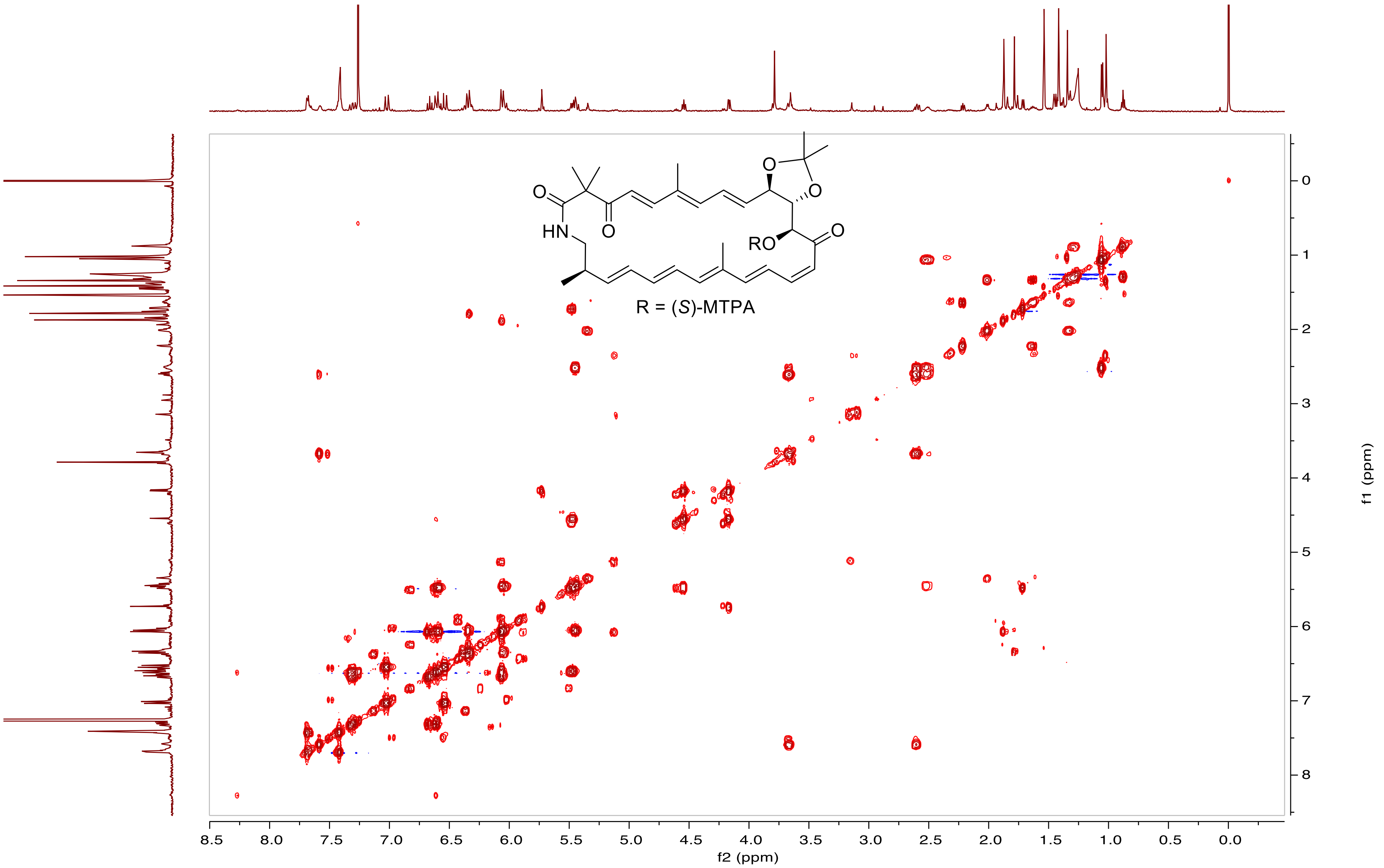

Figure S43. ^{1}H-NMR spectrum (600 MHz) of (*R*)-MTPA ester (**1bb**) of **1b** in $CDCl_3$

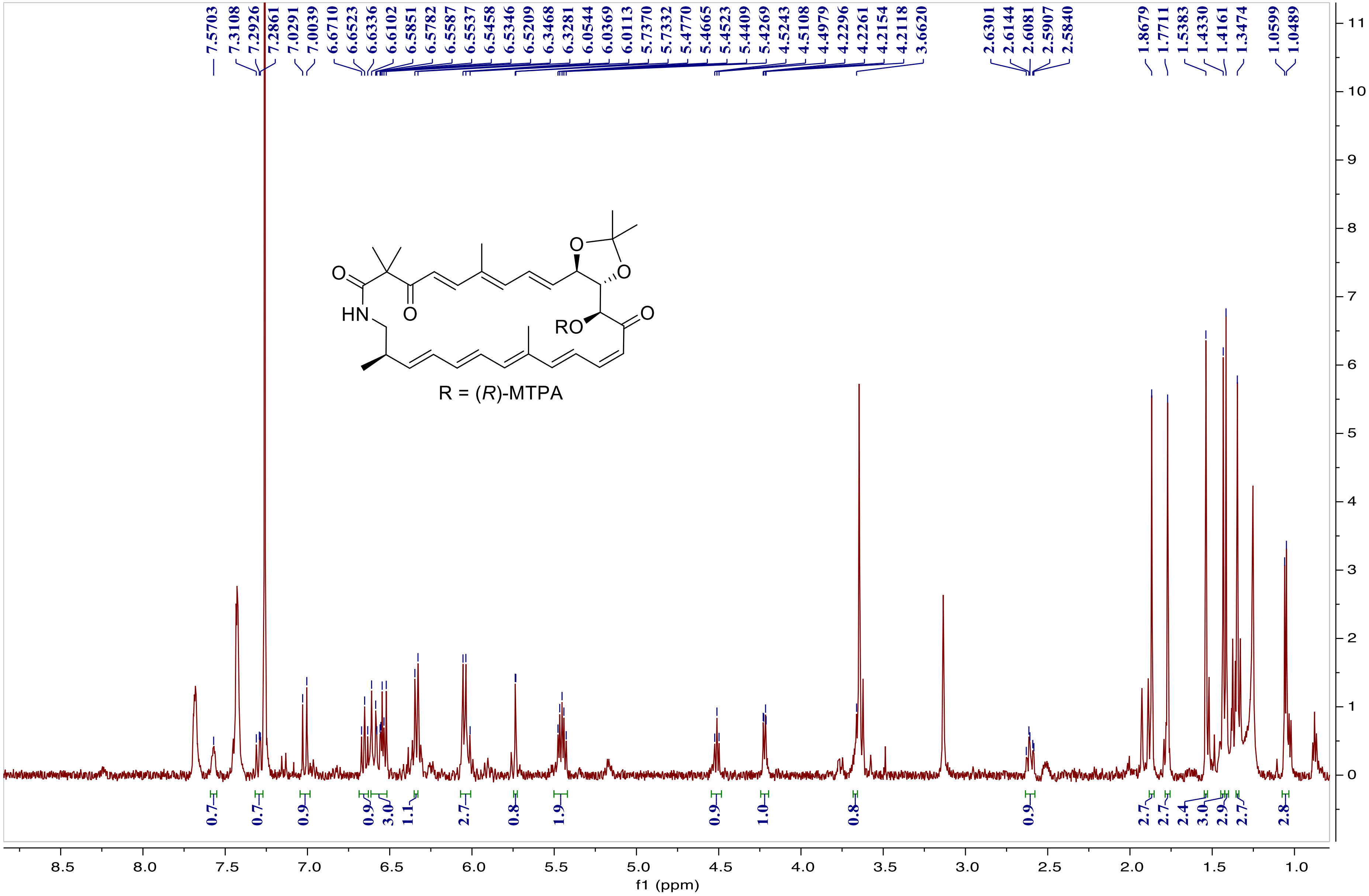

Figure S44. ^{1}H-^{1}H COSY spectrum (600 MHz) of (*R*)-MTPA ester (**1bb**) of **1b** in $CDCl_3$

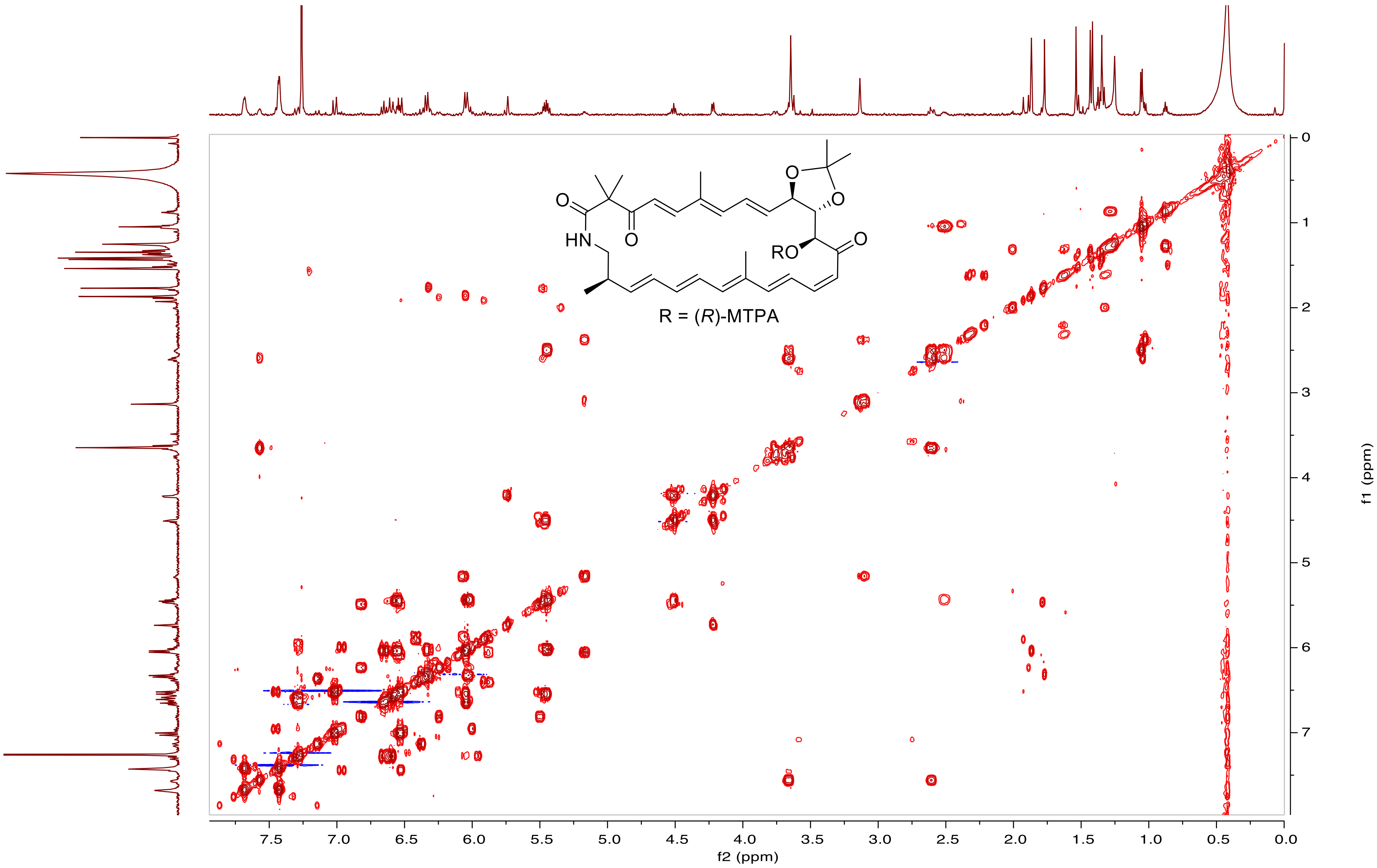

Figure S45. ^{1}H-NMR spectrum (600 MHz) of compound **4a** in DMSO-d_6

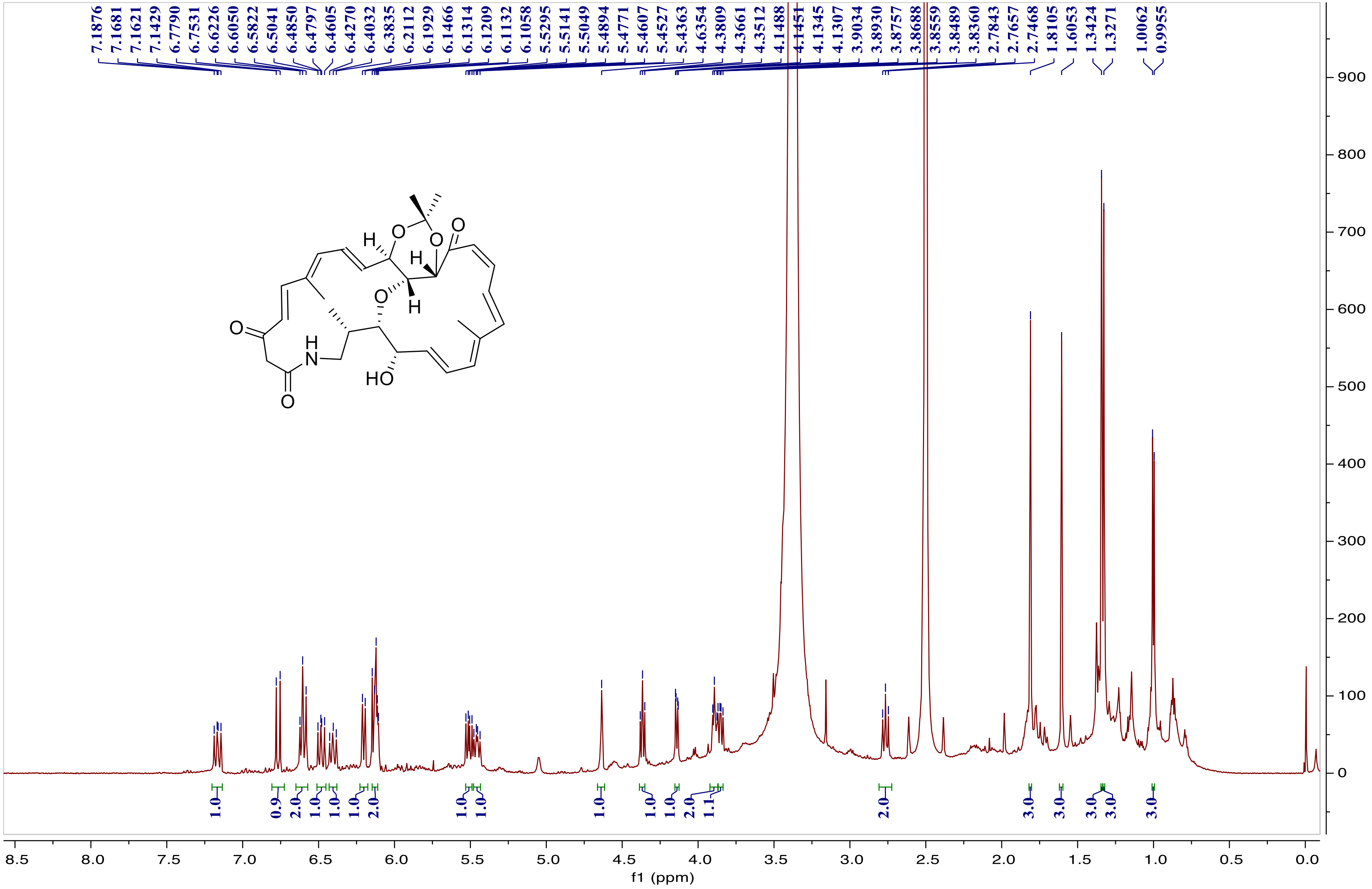

Figure S46. ^{1}H-^{1}H COSY spectrum (600 MHz) of compound **4a** in DMSO-d_6

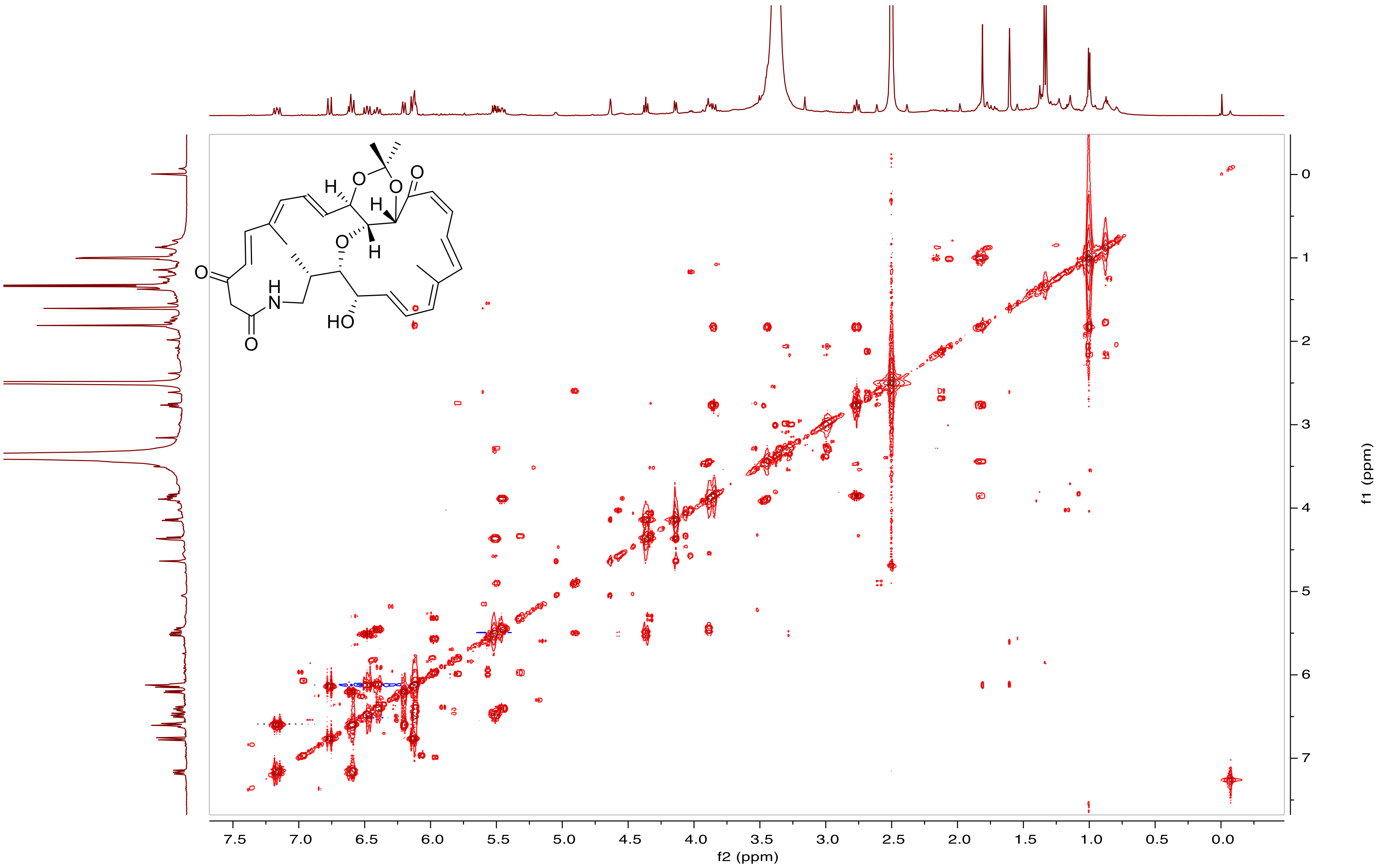

Figure S47. NOESY spectrum (600 MHz) of compound **4a** in DMSO-d_6

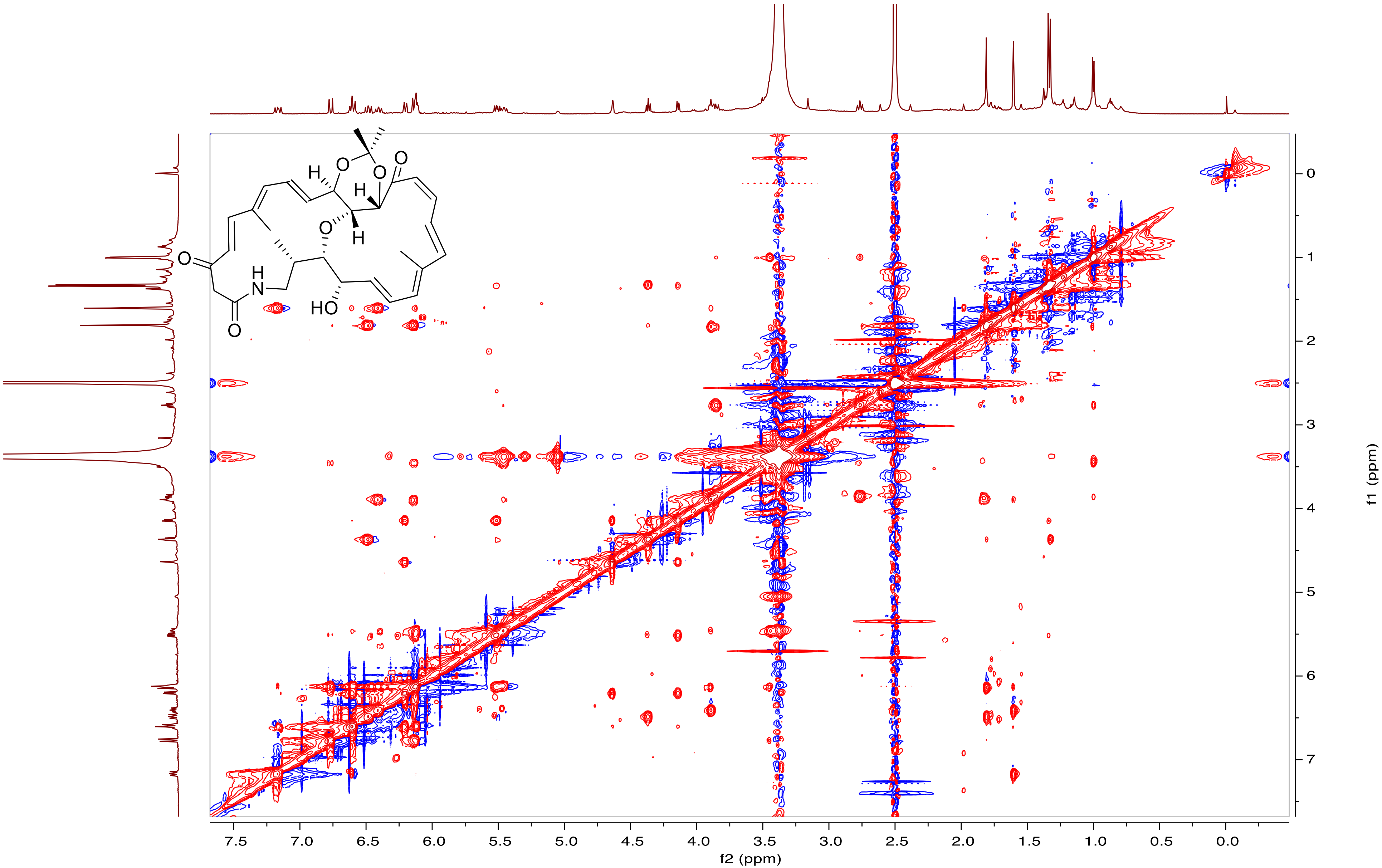

Figure S48. HRESIMS spectrum of **4a**

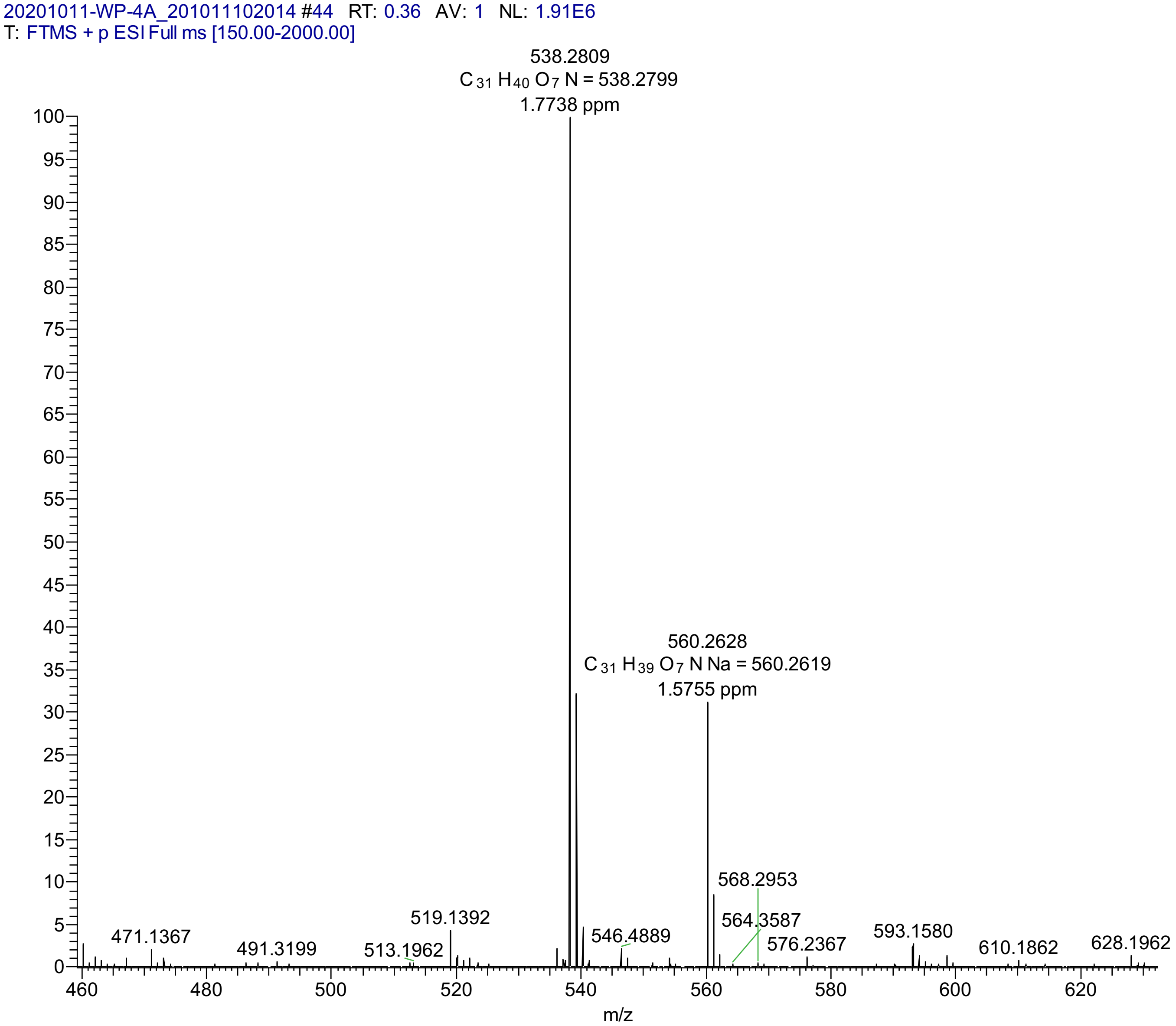

Supplement: Supplementary file 1 [file marinedrugs-19-00013-s001.pdf]
